# The Hippo pathway drives the cellular response to hydrostatic pressure

**DOI:** 10.15252/embj.2021108719

**Published:** 2022-06-15

**Authors:** Jiwon Park, Siyang Jia, Donald Salter, Pierre Bagnaninchi, Carsten G Hansen

**Affiliations:** ^1^ Centre for Inflammation Research Institute for Regeneration and Repair, Edinburgh bioQuarter The University of Edinburgh Edinburgh UK; ^2^ Centre for Regenerative Medicine Institute for Regeneration and Repair, Edinburgh bioQuarter The University of Edinburgh Edinburgh UK; ^3^ Centre for Genomic & Experimental Medicine MRC Institute of Genetics & Molecular Medicine The University of Edinburgh, Western General Hospital Edinburgh UK

**Keywords:** cell volume, endocytosis, holographic imaging, membrane tension, YAP/TAZ, Cell Adhesion, Polarity & Cytoskeleton, Membranes & Trafficking, Signal Transduction

## Abstract

Cells need to rapidly and precisely react to multiple mechanical and chemical stimuli in order to ensure precise context‐dependent responses. This requires dynamic cellular signalling events that ensure homeostasis and plasticity when needed. A less well‐understood process is cellular response to elevated interstitial fluid pressure, where the cell senses and responds to changes in extracellular hydrostatic pressure. Here, using quantitative label‐free digital holographic imaging, combined with genome editing, biochemical assays and confocal imaging, we analyse the temporal cellular response to hydrostatic pressure. Upon elevated cyclic hydrostatic pressure, the cell responds by rapid, dramatic and reversible changes in cellular volume. We show that YAP and TAZ, the co‐transcriptional regulators of the Hippo signalling pathway, control cell volume and that cells without YAP and TAZ have lower plasma membrane tension. We present direct evidence that YAP/TAZ drive the cellular response to hydrostatic pressure, a process that is at least partly mediated via clathrin‐dependent endocytosis. Additionally, upon elevated oscillating hydrostatic pressure, YAP/TAZ are activated and induce TEAD‐mediated transcription and expression of cellular components involved in dynamic regulation of cell volume and extracellular matrix. This cellular response confers a feedback loop that allows the cell to robustly respond to changes in interstitial fluid pressure.

## Graphical abstract

Oscillating hydrostatic pressure induces changes in cell volume by activating transcriptional co‐activators YAP/TAZ via a process involving clathrin‐dependent endocytosis and actin cytoskeleton.

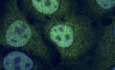

## Introduction

Cells constantly need to sense and dynamically integrate multiple chemical and mechanical stimuli within the cellular microenvironment (Hansen *et al*, 2015a; Vining & Mooney, 2017; Cadart *et al*, 2019; Collinet & Lecuit, 2021). Physical cues in the cellular niche are critical mediators of context‐dependent cellular regulation and responses (Hansen *et al*, 2015a; Vining & Mooney, 2017; Cadart *et al*, 2019; Collinet & Lecuit, 2021). Consequently, in‐depth understanding of how cells respond to these distinct forces are of major interest. These fundamental responses modulate central cellular processes, such as metabolic adaptation, differentiation, proliferation, cellular migration and cell size, and thereby ultimately define cell identity (Hansen *et al*, 2015a; Vining & Mooney, 2017; Cadart *et al*, 2019; Collinet & Lecuit, 2021). A less well‐understood cellular process is the ability to precisely and dynamically respond to changes in interstitial fluid pressure (IFP). This pressure is distinct from other types of cellular mechanical stimuli, as IFP is isotropic and affects the thermodynamics of the cellular environment without applying a vector force (Heldin *et al*, 2004; Myers *et al*, 2007). Consequently, fluid pressure is fundamentally different from other mechanical stresses. IFP oscillates in places almost in phase with arterial pressure (Myers *et al*, 2007). IFP is of particular importance not only during development (Stewart *et al*, 2011; Mirra *et al*, 2019; Teng & Engler, 2019; Chan & Hiiragi, 2020) but also during pathophysiological processes, such as inflammation, oedema and in solid tumours (Heldin *et al*, 2004; Stewart *et al*, 2011; Wiig & Swartz, 2012; Teng & Engler, 2019). Tumours with elevated IFP facilitate the migration of cancer cells from the tumour into the tissue and correlate with poor prognosis (Heldin *et al*, 2004; Northcott *et al*, 2018). Various factors may contribute to elevated IFP in solid tumours, including blood vessel leakiness, contraction mediated by stromal fibroblasts, overall changes to the interstitial matrix composition, and lymph vessel abnormalities (Heldin *et al*, 2004; Myers *et al*, 2007; Swartz & Lund, 2012; Wiig & Swartz, 2012).

The Hippo pathway controls development and facilitates regenerative processes through regulating its transcriptional co‐activators YAP and TAZ, and can cause cancer if the pathway is not tightly regulated (Moroishi *et al*, 2015a; Fulford *et al*, 2018; Davis & Tapon, 2019; Rognoni & Walko, 2019; Salem & Hansen, 2019; Zanconato *et al*, 2019; Thompson, 2020). The Hippo pathway contains an upstream serine/threonine kinase module and a downstream transcriptional effector module, consisting of YAP and TAZ (encoded, respectively, by *YAP1* and *WWTR1)* and their cognate transcription factors (Hansen *et al*, 2015a; Fulford *et al*, 2018). YAP/TAZ are regulated by LATS1/2‐mediated inhibitory phosphorylation on five (YAP) or four (TAZ) serine residues (Huang *et al*, 2005; Zhao *et al*, 2007; Liu *et al*, 2010). Upon relief from this inhibitory phosphorylation, YAP and TAZ localize to the nucleus to exert their co‐transcriptional activity (Huang *et al*, 2005; Zhao *et al*, 2007; Liu *et al*, 2010). In solid tumours, high YAP/TAZ activity in general increase the risk of metastasis (Steinhardt *et al*, 2008; Lamar *et al*, 2012), impede cancer treatment and confer poor prognosis (Moroishi *et al*, 2015a; Rognoni & Walko, 2019; Salem & Hansen, 2019; Zanconato *et al*, 2019; Thompson, 2020). However, distinct core Hippo pathway components are mutated only in a subset of cancers, and the underlying reasons as to why YAP/TAZ are predominantly nuclear in solid tumours are not fully understood (Moroishi *et al*, 2015a; Fulford *et al*, 2018; Rognoni & Walko, 2019; Salem & Hansen, 2019; Zanconato *et al*, 2019; Thompson, 2020). The Hippo pathway is a transducer of physical stimuli in the microenvironment and a nexus for cellular signalling (Hansen *et al*, 2015a; Rausch & Hansen, 2020). The Hippo pathway is linked to cellular responses to extracellular matrix (ECM) stiffness (Dupont *et al*, 2011; Liu *et al*, 2015; Bertero *et al*, 2016; Meng *et al*, 2018), shear stress (Wang *et al*, 2016; Lee *et al*, 2017; Nakajima *et al*, 2017; Rausch *et al*, 2019) and osmotic pressure (Hong *et al*, 2017; Meng *et al*, 2018). Importantly, the role of the pathway in cellular response to elevated hydrostatic pressure has so far been unexplored. As the Hippo pathway is a mechanotransductive pathway, we sought to establish if the Hippo pathway is a mediator of cellular response to interstitial fluid pressure. We took advantage of a panel of isogenic genome‐edited cells (Hansen *et al*, 2015b; Meng *et al*, 2015; Lin *et al*, 2017; Rausch *et al*, 2019), where core components of the pathway have been deleted. This isogenic platform allows us to directly evaluate the impact that components within the Hippo pathway has on fundamental cellular functions. We here provide evidence that changes in hydrostatic pressure are sensed at the plasma membrane via regulation of clathrin‐mediated endocytosis, which is transduced to the core Hippo pathway kinase module and initiates downstream YAP/TAZ‐TEAD‐dependent cellular effects. This highlights how hydrostatic forces, plasma membrane tension and plasma membrane dynamics stimulate intracellular signals to regulate cell functions.

## Results

### Oscillating hydrostatic pressure activates YAP/TAZ

**Figure 1:**
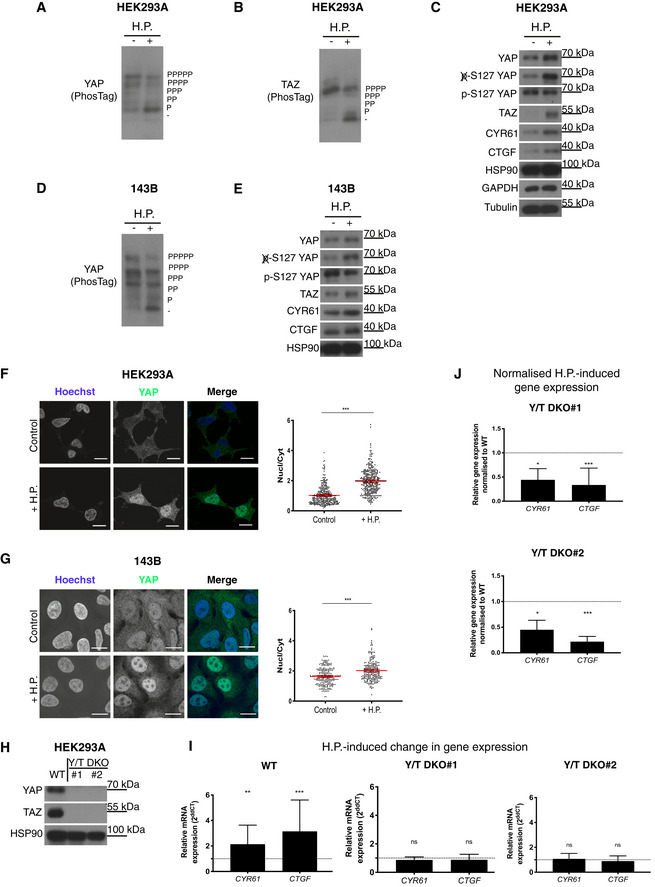
Hydrostatic pressure activates YAP/TAZ PhosTag‐based Western blot probed for YAP reveals increased levels of unphosphorylated (downshift) YAP in HEK293A cells in response to cyclic 0.1 Hz 200 mbar hydrostatic pressure (+) compared to control (−).PhosTag‐based Western blot probed for TAZ reveals increased levels of unphosphorylated (downshift) TAZ in HEK293A cells in response to cyclic 0.1 Hz 200 mbar hydrostatic pressure (+) compared to control (−).Western blots from cell lysates as in (A) and (B) show total protein levels in control (−) and in cell lysates obtained from cells exposed to hydrostatic pressure (+) stimulation in HEK293A cells. Note the decrease in the inhibitory p127 YAP signal as well as increase in YAP/TAZ targets CTGF and CYR61 levels upon hydrostatic pressure.PhosTag‐based Western blot probed for YAP reveals increased levels of unphosphorylated (downshift) YAP in 143B cells in response to hydrostatic pressure (+) compared to control (−).Western blot shows total protein levels in control (−) and with hydrostatic pressure (+) stimulation in 143B cells from cell lysates as in D.HEK293A cells at steady state (upper) or upon cyclic 0.1 Hz 200 mbar hydrostatic pressure (lower) were analysed by immunofluorescence. Cells are labelled for Hoechst (blue) and YAP (green). Scale bar = 20 μm. Graph on the right depict quantification of nuclear‐to‐cytoplasmic (Nucl/Cyt) ratio of YAP in cells as shown on the left. Each dot represents a single cell and data are pooled from three independent experiments. Error bars represent mean ± 95% CI. Mann–Whitney U test. ***P < 0.001.143B cells at steady state (upper) or upon cyclic 0.1 Hz 200 mbar hydrostatic pressure (lower). Graph on the right shows quantification of nuclear‐to‐cytoplasmic (Nucl/Cyt) ratio of YAP in 143B from images as those shown on the left. Each dot represents a single cell and data are pooled from three independent experiments. Error bars represent mean ± 95% CI. Mann‐Whitney U test. ***P < 0.001.Western blot confirming no YAP and TAZ expression in two independent (#1 and #2) YAP/TAZ double‐knockout (Y/T DKO) clones.Relative expression levels of YAP/TAZ target genes *CYR61* and *CTGF* in HEK293A WT and Y/T DKO clones in response to cyclic 0.1 Hz 200 mbar hydrostatic pressure. Data from six independent experiments. Error bars represent mean ± SD. Kruskal–Wallis test with Dunn’s *post‐hoc*. **P = 0.00661 (WT *CYR61)*, ***P = 0.0001 (WT *CTGF*), *P* = 0.3924 (Y/T DKO#1 *CYR61*), *P* = 0.4616 (Y/T DKO#1 *CTGF*), *P* > 0.999 (Y/T DKO#2 *CYR61*) and *P* = 0.0782 (Y/T DKO#2 *CTGF*).
*CYR61* and *CTGF* gene expression levels induced by hydrostatic pressure in (I) of Y/T DKO #1 and #2 normalized to WT. Kruskal‐Wallis test with Dunn’s *post‐hoc*. *P = 0.0259 (Y/T DKO#1 *CYR61*), ***P < 0.001 (Y/T DKO#1 *CTGF*), *P = 0.0155 (Y/T DKO#2 *CYR61*) and ***P < 0.001 (Y/T DKO#2 *CTGF*). Source data are available online for this figure.

**Figure EV1:**
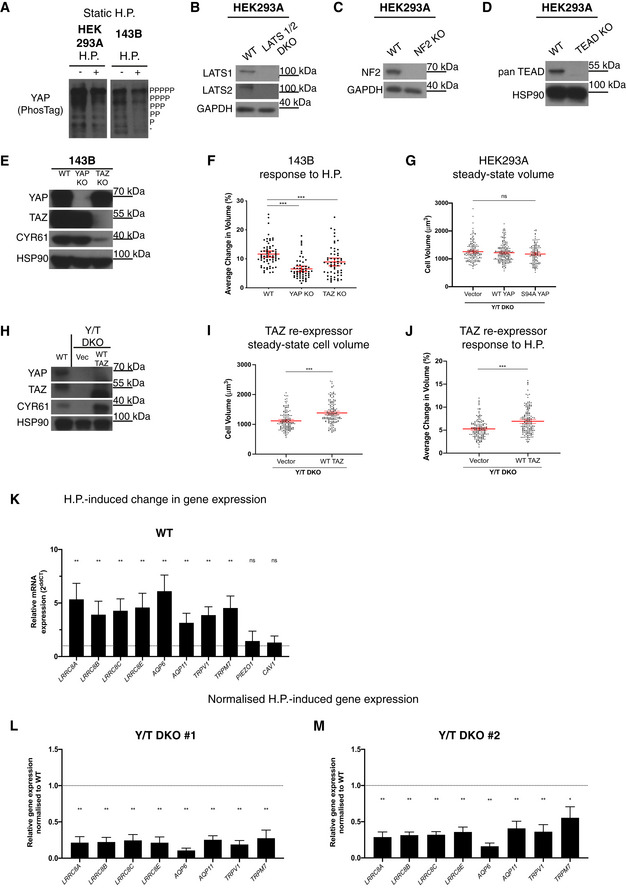
YAP/TAZ regulate cell responses to hydrostatic pressure PhosTag‐based Western blot probed for YAP reveals no changes in YAP phosphorylation levels in HEK293A and 143B cells in response to static 500 mbar hydrostatic pressure (+) compared to control (−).Western blot showing LATS1 and LATS2 levels in WT and LATS1/2 DKO cells.Western blot showing NF2 expression level in WT and NF2 KO cells.Western blot showing TEAD expression level in WT and TEAD KO cells.Western blot showing YAP, TAZ and CYR61 levels in 143B YAP KO and TAZ KO cells relative to WT.143B WT, YAP KO and TAZ KO response to 100 mbar cyclic hydrostatic pressure as measured by DHM. Each dot represents a single cell from three independent experiments and error bars represent mean ± 95% CI. Mann–Whitney U test. ***P < 0.001 (WT vs. YAP KO), ***P = 0.006 (WT vs. TAZ KO).Steady‐state volume of Y/T DKO relative to WT YAP and S94A mutant YAP re‐expressing Y/T DKO cells as measured by DHM. Each dot represents a single cell from three independent experiments and error bars represent 95% CI. Kruskal–Wallis test with Dunn’s *post‐hoc*. *P* > 0.999 (vector vs. WT YAP), *P* = 0.2173 (vector vs. S94A YAP) and, *P* = 0.9837 (WT YAP vs. S94A YAP).Western blot showing total YAP, TAZ and CYR61 levels in WT TAZ re‐expressing cells compared to WT and Y/T DKO vector control.Steady‐state cell volume WT TAZ re‐expressing cells relative to Y/T DKO as imaged and analysed by DHM. Each dot represents a single cell from three independent experiments. Error bars represent mean ± 95% CI. Mann–Whitney U test. ***P < 0.001.Average change in cell volume in response to hydrostatic pressure in WT TAZ re‐expressing cells relative to Y/T DKO as imaged and analysed by DHM. Each dot represents a single cell from three independent experiments. Error bars represent mean ± 95% CI. Mann–Whitney U test. ***P < 0.001.Candidate genes conferring YAP/TAZ‐mediated cellular response to hydrostatic pressure. Gene expression levels of HEK293A WT cells in response to 0.1 Hz, 200 mbar cyclic hydrostatic pressure (4 h) compared with steady‐state levels within each genotype and analysed by RT–qPCR. Graphs show data obtained from four independent experiments. Error bars represent mean ± SD. Kruskal–Wallis test with Dunn’s *post‐hoc*.Gene expression level induced by hydrostatic pressure in Y/T DKO#1 cells normalized to WT levels. Graphs show data obtained from four independent experiments. Error bars represent mean ± SD. Kruskal–Wallis test with Dunn’s *post‐hoc*.Gene expression level induced by hydrostatic pressure in Y/T DKO#2 cells normalized to WT levels. Graphs show data obtained from four independent experiments. Error bars represent mean ± SD. Kruskal–Wallis test with Dunn’s *post‐hoc*. Source data are available online for this figure.

Initially, we examined if the Hippo pathway is responsive to hydrostatic pressure and examined 200 mbar, readily within the pathophysiological range of cellular interstitial fluid pressures (Heldin *et al*, 2004; DuFort, 2016). HEK293A cells were cultured with or without cyclic hydrostatic pressure for 2 h, whereafter lysates were prepared and analysed by PhosTag gels (Fig 1A and B). YAP and YAZ are inhibited by LATS1/2‐mediated phosphorylation on multiple sites (Zhao *et al*, 2007; Mo *et al*, 2012; Yu *et al*, 2012; Meng *et al*, 2015; Moroishi *et al*, 2015b; Park *et al*, 2015). Consequently, the PhosTag technique allows for determination of YAP and TAZ phosphorylation levels, and thereby activation status (Zhao *et al*, 2007; Mo *et al*, 2012; Yu *et al*, 2012; Meng *et al*, 2015; Moroishi *et al*, 2015b; Park *et al*, 2015). A clear downshift (dephosphorylation) and, therefore, activation of YAP (Fig 1A) and TAZ (Fig 1B) are observed in cells experiencing cyclic hydrostatic pressure. To further confirm that the observed changes in dephosphorylation levels cause an increase in YAP/TAZ activity levels, we analysed the same lysates by conventional Western blots (Fig 1C). Increased levels of YAP and TAZ were evident in these experiments consistent with elevated YAP and TAZ activity. Using phospho‐specific YAP antibodies raised against S127, a major LATS site (Zhao *et al*, 2007), as well as using antibodies that exclusively recognizes S127 when YAP is not phosphorylated on this site (Si *et al*, 2017) (Fig 1C), confirmed results obtained by PhosTag gels. Protein levels of two well‐established YAP/TAZ targets, CTGF (*CCN2*) and CYR61 (*CCN1*), are likewise increased upon cyclic hydrostatic pressure (Fig 1C). These data show that YAP/TAZ are dephosphorylated on inhibitory phosphorylation sites upon increased cyclic pressure, and that protein levels of well‐established YAP/TAZ‐encoded target genes are increased upon oscillating hydrostatic pressure (Fig 1C). We next examined if this cellular response is conserved in additional cell types. As elevated IFP is well established in primary bone cancer (Nathan *et al*, 2005, 2008; Matsubara *et al*, 2013; Ariffin *et al*, 2014), we examined the osteosarcoma‐derived cell line 143B. Indeed in 143B cells, as in HEK293A cells, YAP becomes dephosphorylated upon oscillating hydrostatic pressure (Fig 1D and E). Furthermore, total 143B cellular levels of YAP and TAZ, likely due to increased stability of unphosphorylated protein (Zhao *et al*, 2010), as well as CTGF and CYR61, are elevated upon cyclic hydrostatic pressure (Fig 1E) mirroring the effect observed in HEK293A cells. We next examined cells experiencing comparable levels of static hydrostatic pressure and observed no YAP activation (Fig EV1A), which highlights that cells sense and respond to dynamic changes. A major point of YAP/TAZ regulation is via nuclear/cytoplasmic shuttling, as increased LATS1/2 phosphorylation renders YAP and TAZ cytoplasmic (Zhao *et al*, 2010, 2012; Mo *et al*, 2012; Yu *et al*, 2012; Meng *et al*, 2015; Moroishi *et al*, 2015b; Park *et al*, 2015). Therefore, we next examined the subcellular localization of YAP in HEK293A cells experiencing cyclic hydrostatic pressure. As predicted from our immunoblots (Fig 1A–C), YAP translocate to the nucleus upon oscillating hydrostatic pressure (Fig 1F). In parallel experiments, we analysed YAP localization in 143B cells. As in HEK293A cells, YAP translocate to the nucleus upon elevated hydrostatic pressure (Fig 1G). We next took advantage of genome‐edited YAP/TAZ‐deficient cells (Hansen *et al*, 2015b) (Fig 1H) to establish if YAP/TAZ drive the transcriptional regulation of the cellular response to hydrostatic pressure. We analysed the levels of the well‐established YAP/TAZ target genes *CYR61* and *CTGF* in cells challenged with cyclic hydrostatic pressure and compared those to cells at steady state. A clear induction of *CYR61* and *CTGF* encoding extracellular matrix proteins is evident in cells experiencing oscillating fluid pressure (Fig 1I). Comparing this effect to the cellular response in YAP/TAZ knockouts (Y/T DKO) (Fig 1H) allowed us to establish that this cellular response to hydrostatic pressure is dependent on YAP/TAZ (Fig 1I and J). These data combined confirm that YAP/TAZ are activated upon oscillating hydrostatic pressure.

### YAP/TAZ regulate cell volume

**Figure 2:**
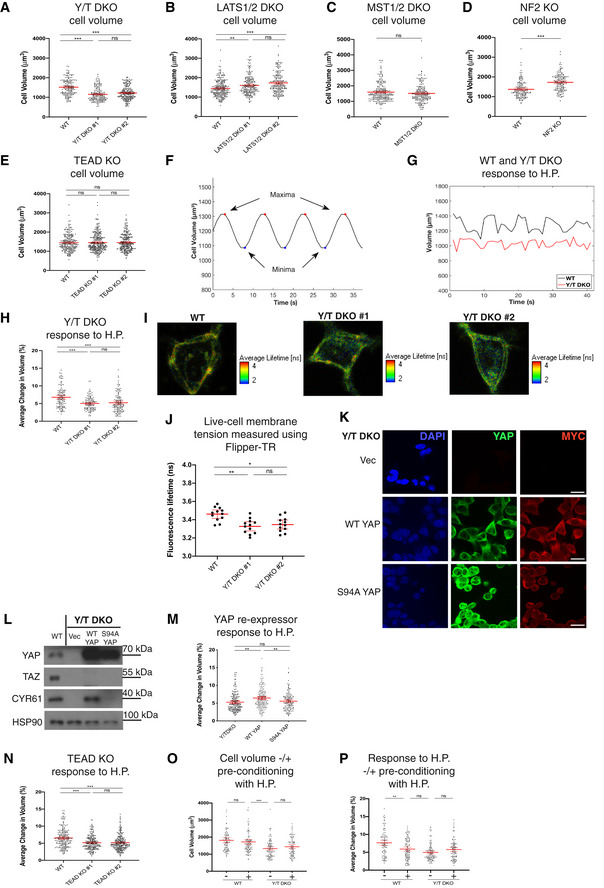
YAP/TAZ determine cell size and mediate cellular response to hydrostatic pressure Cell volume of WT and Y/T DKO clones at steady state measured using DHM. Each dot represents a single cell. Error bars represent mean ± 95% CI. Kruskal–Wallis test with Dunn’s *post‐hoc*. Data from three independent experiments. ***P < 0.001 and *P* = 0.0917 (Y/T DKO #1 vs. #2).Comparison of cell volume of WT and LATS1/2 DKO clones at steady state obtained using DHM. Each dot represents a single cell. Data from three independent experiments. Error bars represent mean ± 95% CI. Kruskal–Wallis test with Dunn’s *post‐hoc*. **P = 0.0018 (WT vs. LATS1/2 DKO #1), ***P < 0.001 (WT vs. LATS1/2 DKO #2) and *P* = 0.0557 (LATS1/2 DKO #1 vs. #2).Comparison of cell volume of WT and MST1/2 DKO at steady state obtained using DHM. Each dot represents a single cell. Data from three independent experiments. Error bars represent mean ± 95% CI. Mann–Whitney U test. *P* = 0.2731.HEK293A NF2 KO cells response to 100 mbar cyclic hydrostatic pressure. Each dot represents a single cell and error bars represent mean ± 95% CI. Data pooled from three independent experiments. Mann–Whitney U test. *P* = 0.4072.Comparison of cell volume of WT and TEAD KO clones at steady state obtained using DHM. Each dot represents a single cell. Data from three independent experiments. Error bars represent mean ± 95% CI. Kruskal–Wallis test with Dunn’s *post‐hoc*. *P* > 0.9999 for all comparisons.Maxima and minima are identified to calculate average percentage change in cell volume in response to hydrostatic pressure using measurements obtained by DHM.Representative WT and Y/T DKO single‐cell volume change in response to hydrostatic pressure recorded by DHM.Average change in cell volume in response to hydrostatic pressure in WT and Y/T DKO clones. Each dot represents a single cell. Images and data were acquired at the onset of oscillating pressure. Data from three independent experiments. Error bars represent mean ± 95% CI. Kruskal–Wallis test with Dunn’s *post‐hoc*. ***P < 0.001 (for both comparisons) and *P* = 0.1450 (Y/T DKO #1 vs. #2).Representative images of Flipper‐TR‐labelled HEK293A WT (top), Y/T DKO #1 (middle) and Y/T DKO #2 (bottom) are shown obtained using fluorescence lifetime imaging microscopy (FLIM) (ex. 485, em. 600)Average fluorescence lifetime of Flipper‐TR probe quantified using fluorescence lifetime imaging microscope (FLIM) (ex. 485, em. 600). HEK293A wild‐type and Y/T DKO clones #1 and #2 were labelled with 2 μM Flipper‐TR for 15 min prior to imaging. The average lifetime measurements were obtained by fitting the photon histogram with a double exponential and the longest lifetime was extracted to report plasma membrane tension, as previously described (Colom *et al*, 2018). Each dot represents fluorescence lifetime of a single cell and data shown are pooled from three independent experiments (mean ± 95% CI). Kruskal–Wallis test with Dunn’s *post‐hoc*. **P = 0.0027 (WT vs. Y/T DKO#1), *P = 0.0136 (WT vs. Y/T DKO #2) and *P* > 0.9999 (Y/T DKO#1 vs. #2).Representative immunofluorescence images showing MYC‐tagged YAP expression in WT YAP re‐expressing cells and S94A mutant YAP re‐expressing cells compared to Y/T DKO vector control. 95% were positive for expression of YAP or YAP mutant. Scale bar = 20 μm.Western blot showing total YAP, TAZ and CYR61 levels in WT YAP and S94A mutant YAP re‐expressing cells compared to WT and Y/T DKO vector control.Average change in cell volume in response to hydrostatic pressure in WT YAP and S94A mutant YAP re‐expressing cells relative to Y/T DKO. Each dot represents a single cell. Error bars represent mean ± 95% CI. Data pooled from three independent experiments. Kruskal–Wallis test with Dunn’s *post‐hoc*. *P* < 0.001 (vector vs. WT YAP), *P* = 0.4912 (vector vs. S94A YAP), *P* = 0.0327Average change in cell volume in response to 100 mbar cyclic hydrostatic pressure in WT and TEAD KO clones. Each dot represents a single cell. Data pooled from three independent experiments. Error bars represent mean ± 95% CI. Kruskal–Wallis test with Dunn’s *post‐hoc*. ***P < 0.001 (for both comparisons) and *P* > 0.9999 (TEAD KO #1 vs. #2).WT and Y/T DKO cell volume were measured at steady state and the average change in cell volume was quantified using the DHM as in previous experiments; these are labelled (−). Cells were then subjected to cyclic hydrostatic pressure for 2 h and their cell volume was quantified to determine whether prior exposure to hydrostatic pressure (“pre‐conditioning”) would change their cell volume response; these are labelled (+). Each dot represents a single cell and error bars represent 95% CI. Graphs include data obtained from four independent experiments. Kruskal–Wallis with Dunn’s *post‐hoc*. ns = *P* > 0.9999.The average percentage change in cell volume was quantified under the same experimental conditions as in “O”. The average change in cell volume in response to cyclic hydrostatic pressure of cells with no previous exposure to hydrostatic pressure (labelled as (−)) was quantified using DHM and compared to those with prior exposure to hydrostatic pressure (labelled (+)). Each dot represents a single cell and error bars represent 95% CI. Graphs include data obtained from four independent experiments. Kruskal–Wallis with Dunn’s *post‐hoc*. **P = 0.006 (WT), *P* = 0.4173 (Y/T DKO) and ns = *P* > 0.9999. Source data are available online for this figure.

To establish the macroscopic cellular response to hydrostatic pressure, we took advantage of live cell digital holographic imaging (DHM). DHM is a technique that allows for quantitative label‐free cellular imaging with single‐cell resolution (Marquet *et al*, 2005). Initially, we examined the cellular volume across genotypes (Fig 2A–D) derived from the optical volume under the assumptions described in the methods. The average cellular volume of two independent YAP/TAZ knockout clones (Hansen *et al*, 2015b) are 1,154 ± 348 μm^3^ (mean ± SD) (Y/T DKO#1) and 1,273 ± 352 μm^3^ (mean ± SD) (Y/T DKO#2), whereas the average cell volume of wild‐type cells is 1,532 ± 434 μm^3^ (mean ± SD). Y/T DKO cells are therefore ~ 16–25% smaller than wild‐type cells (Fig 2A). These data confirm recent reports highlighting that YAP/TAZ regulate cell size via signalling to mTORC1, and through other less defined mechanisms (Hansen *et al*, 2015b; Plouffe *et al*, 2018; Perez‐Gonzalez *et al*, 2019; Mugahid *et al*, 2020). LATS1/2 directly phosphorylate and thereby inhibit YAP/TAZ and are the major cellular regulators of YAP/TAZ (Zhao *et al*, 2010, 2012; Mo *et al*, 2012; Yu *et al*, 2012; Meng *et al*, 2015; Moroishi *et al*, 2015b; Park *et al*, 2015). YAP/TAZ are consequently nuclear and constitutively activated in LATS1/2 DKO cells (Meng *et al*, 2015; Park *et al*, 2015). Consistent with YAP/TAZ DKO cells being smaller, the average cell volume of LATS1/2 DKO with hyperactive YAP/TAZ are 1,603 ± 473 μm^3^ (mean ± SD) (LATS1/2 DKO#1) and 1,731 ± 526 μm^3^ (mean ± SD) (LATS1/2 DKO#2). The larger cell volume of LATS1/2 DKO cells compared to WT (Figs 2B and EV1B) confirms previous observations (Hansen *et al*, 2015b).

These data firmly establish our ability to precisely measure the cellular volume change associated with modification in the Hippo pathway. Using this approach, together with the isogenic Hippo pathway component knockout models (Hansen *et al*, 2015b; Meng *et al*, 2015; Park *et al*, 2015; Lin *et al*, 2017), allow us to delineate the potential role of discrete Hippo pathway components on cell volume.

MST1/2 phosphorylate and activate LATS1/2 (Hansen *et al*, 2015a; Moroishi *et al*, 2015a). To gain mechanistic insights into cellular volume regulation, we went on to determine the cell volume of cells without the Hippo kinases (MST1/2) (Huang *et al*, 2005; Meng *et al*, 2015). MST1/2 DKO cells are of similar size as WT cells (Fig 2C). MST1/2 DKO cells therefore do not phenocopy LATS1/2 DKO cells with respect to cellular volume. This is likely due to further regulation of LATS1/2 by MST1/2 compensating LATS1/2 activating kinases (Li *et al*, 2014b, 2018; Meng *et al*, 2015; Rausch & Hansen, 2020). We also examined the cellular volume of cells devoid of NF2. NF2, also known as MERLIN, is a tumour suppressor and functions as an upstream positive regulator of the Hippo pathway kinases (Zhang *et al*, 2010; Meng *et al*, 2015). NF2 is the most commonly mutated Hippo pathway component in cancers, and NF2 loss‐of‐function mutations are especially prevalent in pleural mesothelioma (Moroishi *et al*, 2015a; Petrilli & Fernandez‐Valle, 2016). YAP/TAZ are hyperactive in cells without NF2 (Moroishi *et al*, 2015a; Petrilli & Fernandez‐Valle, 2016). NF2 KO cells are 25.8% larger than WT cells (1,728 ± 439 μm^3^ (mean ± SD)) (Figs 2D and EV1C) and phenocopy LATS1/2 DKO cells. As the co‐transcriptional activators YAP/TAZ frequently regulate gene expression via binding to the TEAD transcription factors (Vassilev *et al*, 2001; Zhao *et al*, 2008; Zhang *et al*, 2009; Li *et al*, 2010; Lamar *et al*, 2012; Hansen *et al*, 2015b; Park *et al*, 2015; Rausch *et al*, 2019), we examined the cellular volume in cells without TEADs (these cells express low levels of TEAD3 (Lin *et al*, 2017)). Analysing two independent TEAD KO clones revealed that TEADs do not dictate the steady‐state cellular volume (Figs 2E and EV1D), and consequently, the role of YAP/TAZ in regulating the cellular volume at steady state takes place at least partly via additional transcription factors (Hansen *et al*, 2015a).

### Cells respond to oscillating hydrostatic pressure by YAP/TAZ‐TEAD‐dependent rapid volume changes

As the cellular consequences of increased interstitial fluid pressure are not well established (Heldin *et al*, 2004; Myers *et al*, 2007; Li *et al*, 2020), we sought to determine if the force exerted by hydrostatic pressure regulates cell size. To this end, we established a workflow that allows us to analyse the dynamic cellular response to elevated hydrostatic pressure in real time with a temporal resolution of seconds. In our system, the hydrostatic pressure is controlled by a microfluidic pump coupled to closed cell culture chambers, where cells are imaged using digital holographic microscopy (Marquet *et al*, 2005). We are consequently able to dictate the precise and temporal hydrostatic pressure experienced by cells, driven by the extra‐ and intracellular pressure differences, in a physiologically relevant manner while imaging the cells without labelling and at single‐cell resolution.

When the fluid pressure is modulated with a peak‐to‐peak pressure of 100 mbar and a 10 s cycle, a clear corresponding cyclic change in volume for the WT cells (6.79 ± 2.89% (mean ± SD)) (Fig 2F–H) is observed. In contrast, YAP/TAZ DKO cells exhibited a substantially smaller periodic volume change (Y/T DKO#1 5.03 ± 1.88% (mean ± SD) and Y/T DKO#2 4.42 ± 2.02% (mean ± SD)) (Fig 2G and H). This YAP and TAZ dependence on the cellular response to hydrostatic pressure is conserved in 143B cells (Fig EV1E and F). In order to further characterize how cells depleted of YAP/TAZ differ from WT cells, we took advantage of the membrane tension probe “Flipper‐TR” (Colom *et al*, 2018; Riggi *et al*, 2018). The Flipper probe inserts into the lipid bilayer and its mechanophore composition is altered depending on the organization of lipid bilayers. The conformational changes in the probe induced by membrane tension alter the excitation maxima and fluorescence lifetime, which can be quantified using fluorescence lifetime imaging microscopy (FLIM) (Colom *et al*, 2018; Riggi *et al*, 2018). Using this probe and comparing WT to Y/T DKO HEK293A cells, a substantial decrease in membrane tension is observed upon deletion of YAP/TAZ (Fig 1I and J). This highlights a distinct, so far unrecognized, YAP/TAZ‐dependent difference on the physical properties of the plasma membrane.

To further investigate that the effect observed upon hydrostatic pressure in the knockout clones is due to the lack of YAP/TAZ, we reintroduced YAP into YAP/TAZ DKO HEK293A cells (Fig 2K and L). YAP/TAZ do not bind DNA directly but function as transcriptional co‐activators through diverse sets of transcription factors (Hansen *et al*, 2015a), the TEAD family being the most prominent mediator of YAP/TAZ activity (Vassilev *et al*, 2001; Ota & Sasaki, 2008; Zhao *et al*, 2008; Li *et al*, 2010; Lamar *et al*, 2012; Hansen *et al*, 2015b; Huh *et al*, 2019; Rausch *et al*, 2019). We sought to establish if the observed YAP/TAZ contingent cellular response also depends on TEADs and generated separate stable cell lines of MYC‐tagged WT YAP and of a TEAD‐binding deficient YAP (S94A) (Zhao *et al*, 2008; Li *et al*, 2010). Cell populations were > 95% positive for YAP (Fig 2K), and cells re‐expressing WT YAP but not S94A YAP rescue CYR61 expression functionally validating these cell lines (Fig 2L). Introduction of exogenous wild‐type YAP into Y/T DKO cells, but not S94A YAP, rescue the cellular response to cyclic fluid pressure (Fig 2M). Introduction of exogenous TAZ into Y/T DKO cells likewise rescued the cellular response to cyclic fluid pressure (Fig EV1H–J), highlighting that YAP‐ or TAZ‐dependent TEAD activation is sufficient to drive the cellular response to hydrostatic pressure.

**Figure EV2:**
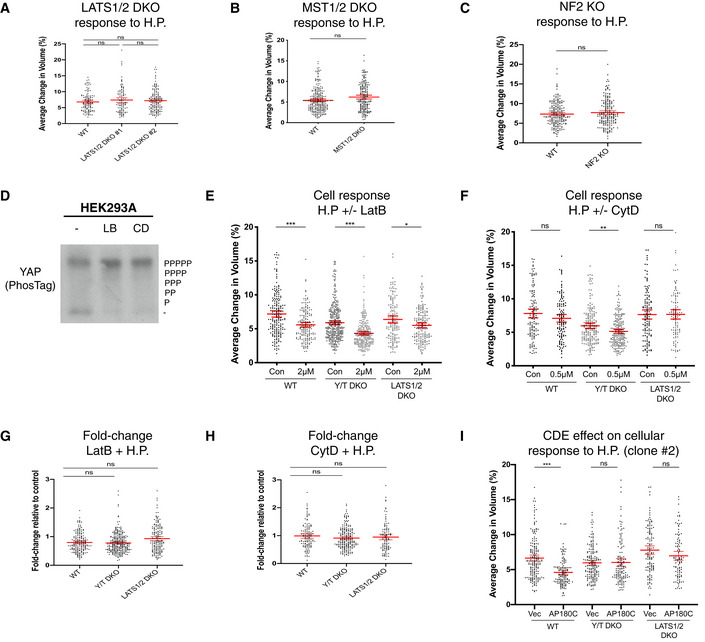
Cytoskeletal implications in the cellular response to hydrostatic pressure Average change in cell volume in response to 100 mbar cyclic hydrostatic pressure in WT and LATS1/2 DKO clones. Each dot represents a single cell. Data pooled from three independent experiments. Error bars represent mean ± 95% CI. Kruskal–Wallis test with Dunn’s *post‐hoc*. *P* > 0.9999 (for all comparisons).Average change in cell volume in response to 100 mbar cyclic hydrostatic pressure in WT and MST1/2 DKO. Each dot represents a single cell. Data pooled from three independent experiments. Error bars represent mean ± 95% CI. Mann–Whitney U test. *P* = 0.0781.HEK293A NF2 KO steady‐state volume compared to WT cells. Each dot represents a single cell and Error bars represent mean ± 95% CI. Data pooled from three independent experiments. Mann–Whitney U test. ***P < 0.001.PhosTag‐based Western blot probing for YAP reveals dephosphorylation of YAP in response to 0.5 μM latrunculin B (LB) and 2 μM cytochalasin D (CD) treatment in WT cells.Comparison of average change in cell volume measured by DHM in response to 100 mbar cyclic hydrostatic pressure with 2 μM latrunculin B treatment in WT, Y/T DKO and LATS1/2 DKO cells. Each cell represents a single cell and error bars represent mean ± 95% CI. Data pooled from four independent experiments. Mann–Whitney U test. ***P < 0.001 (WT), ***P < 0.001 (Y/T DKO) and *P = 0.0331 (LATS1/2 DKO).Comparison of average change in cell volume in response to 100 mbar cyclic hydrostatic pressure with 0.5 μM cytochalasin D treatment in WT, Y/T DKO and LATS1/2 DKO cells. Each cell represents a single cell and error bars represent mean ± 95% CI. Data pooled from four independent experiments. Mann–Whitney U test. *P* = 0.2021 (WT), **P = 0.0035 (Y/T DKO) and *P* = 0.9148 (LATS1/2 DKO).Fold difference in average change in cell volume in response to hydrostatic pressure from B normalized against cells not treated with 2 μM latrunculin B. Each dot represents a single cell and error bars are 95% CI. Mann–Whitney U test. *P* > 0.9999 (WT vs. Y/T DKO) and *P* = 0.0723 (WT vs. LATS1/2 DKO).Fold difference in average change in cell volume in response to hydrostatic pressure from C normalized against cells not treated with 0.5 μM cytochalasin D. Each dot represents a single cell and error bars represent mean ± 95% CI. Mann–Whitney U test. *P* = 0.4619 (WT vs. Y/T DKO) and *P* = 0.7087 (WT vs. LATS1/2 DKO).HEK293A YAP/TAZ DKO and LATS1/2 DKO clone #2’s responses to cyclic 0.1 Hz 200 mbar dynamic hydrostatic pressure with AP180C‐mediated inhibition of clathrin‐dependent endocytosis. Each dot represents a single cell and error bars represent mean ± 95% CI. Data pooled from four independent experiments. Kruskal–Wallis test with Dunn’s *post‐hoc*. ***P < 0.001 (WT), *P* = 0.2522 (Y/T DKO) and *P* = 0.0612 (LATS1/2 DKO). Source data are available online for this figure.

We in a complimentary approach took advantage of TEAD knockout HEK293A cells (Fig EV1D) (Lin *et al*, 2017). We hypothesized that if YAP/TAZ work via TEADs to dictate cellular volume via hydrostatic pressure, TEAD‐deficient cells would phenocopy Y/T DKO cells. Consistent with this, TEAD‐deficient cells closely mirror Y/T DKO cells regarding their cellular response to cyclic fluid pressure (Fig 2N). Consequently, we conclude that the cellular response to hydrostatic pressure is dependent on YAP/TAZ‐TEAD activity. Using our robust cell volume change assay as a read‐out for cellular response to fluid pressure, we next analysed LATS1/2 DKO, MST1/2 DKO and NF2 KO cells. Cells with these genotypes have varying degrees of hyperactive YAP/TAZ (Hansen *et al*, 2015b; Meng *et al*, 2015, 2018), but exhibit similar response to hydrostatic pressure (Fig EV2A–C). This highlights that increased YAP/TAZ levels above WT levels do not change the cell volume response to fluid pressure. We next sought to establish the longer‐term impact on cells upon changes in hydrostatic pressure. We initially measured by DHM the steady‐state cell volume and compared this to the steady‐state volume of cells that had undergone 2 h of oscillating 100 mbar hydrostatic pressure (with pre‐conditioning). Both WT and YAP/TAZ DKO cells do not change their cellular volume after hydrostatic pressure, regardless of prior exposure to hydrostatic pressure (Fig 2O). To obtain insights into if cells adapt their dynamic cell volume response to oscillating hydrostatic pressure, we in similar experiments as above compared the average change in cell volume between cells without any previous exposure to hydrostatic pressure to cells with prior exposure to oscillating hydrostatic pressure (Fig 2P). Cells with prior exposure to oscillating hydrostatic pressure adapt to this by lowering their volume changes, but interestingly this mechanical memory is lost in YAP/TAZ DKO cells (Fig 2P). In order to establish transcriptional regulation of surface molecules for potential long‐term adaption of the cellular response to hydrostatic pressure, we undertook a candidate approach of genes previously shown to be involved in cell volume regulation. We analysed WT cells after 4 h of oscillating hydrostatic pressure stimulation and analysed gene expression and identified a range of specific genes involved in dynamic cell volume regulation that is robustly induced, including *LRRC8A,B,C and E* (Qiu *et al*, 2014; Voss *et al*, 2014; Kefauver *et al*, 2018) encoding components of the heteromeric volume‐regulated anion channel (VRAC), as well as genes encoding homotetrameric aquaporins (*AQP6* and *11*) (Yasui *et al*, 1999), the non‐selective cation channel transient receptor potential vanilloid 1 (*TRPV1*) (Liao *et al*, 2013) and the swelling‐activated TRPM7 cation channels (Schmitz *et al*, 2003; Numata *et al*, 2021), but not other mechanotransductive active plasma membrane components, such as the mechanosensitive ion channel *PIEZO1 (*Coste *et al*, 2010; Li *et al*, 2014a *)* and the essential caveolae gene *CAV1* (Hansen & Nichols, 2010; Rausch *et al*, 2019; Rausch & Hansen, 2020) (Fig EV1K). In order to examine the role of YAP/TAZ in this transcriptional regulation, we analysed the induction of these specific gene sets in each of the two Y/T DKO clones, and compared this to that of WT cells (Fig EV1L and M). There is a marked decrease in the induction of the fold induction of these genes in Y/T DKO cells (Fig EV1L and M). This suggests that the hydrostatic pressure‐mediated transcriptional induction of specific gene sets is YAP/TAZ dependent, and highlights that YAP/TAZ likely also regulate long‐term cellular adaption to hydrostatic pressure.

### Actin cytoskeletal regulation of the cellular volume

The actin cytoskeleton and cortex are regulators and mediators of cellular deformations (Stewart *et al*, 2011; Chugh *et al*, 2017; van Helvert *et al*, 2018). The Hippo pathway is a transducer of mechanical cues and is regulated via actin cytoskeletal changes (Dupont *et al*, 2011; Sansores‐Garcia *et al*, 2011; Yu *et al*, 2012; Zhao *et al*, 2012; Li *et al*, 2018; Meng *et al*, 2018), including via transcriptional regulation of cytoskeletal components and modifiers and thereby controlling the dynamics of the actomyosin network (Zhao *et al*, 2008; Lucas *et al*, 2013; Porazinski *et al*, 2015; Kim *et al*, 2017; Nardone *et al*, 2017; Qiao *et al*, 2017; Mason *et al*, 2019).

**Figure 3:**
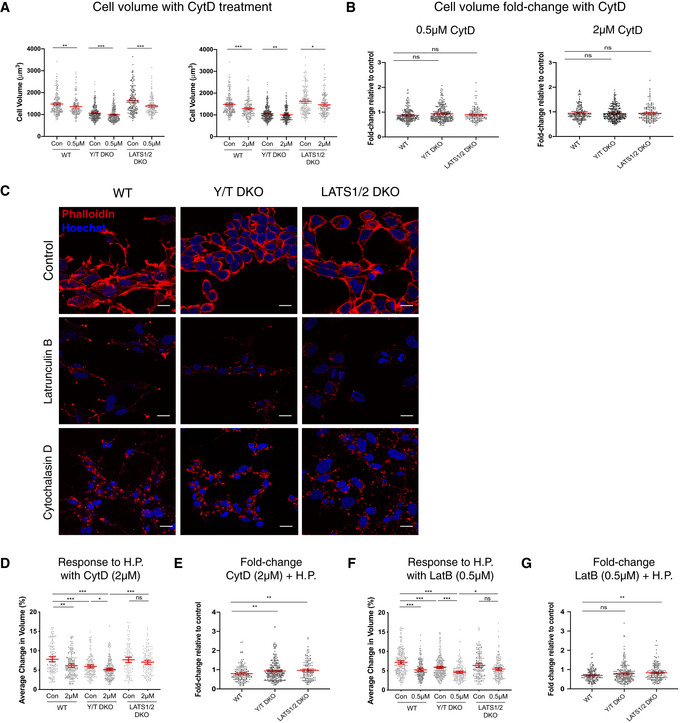
Cellular response to hydrostatic pressure is regulated by the actin cytoskeleton Cell volume measured by DHM of HEK293A cells treated with 0.5 μM (left) and 2 μM (right) cytochalasin D. Each dot represents a single cell and error bars represent mean ± 95% CI. Data pooled from four independent experiments. Mann–Whitney U test. **P = 0.0033 (WT 0.5 μM), ***P < 0.001 (Y/T DKO 0.5 μM), ***P = 0.004(LATS1/2 DKO 0.5 μM), ***P < 0.001 (WT 2 μM), **P = 0.0022 (Y/T DKO 2 μM) and *P = 0.0145 (LATS1/2 DKO 2 μM).Fold change in cell volume upon treatment with 0.5 μM (left) and 2 μM (right) cytochalasin D relative to untreated control. Each dot represents a single cell and error bars represent mean ± 95% CI. Data pooled from four independent experiments. Mann–Whitney U test. *P* = 0.1197 (WT vs. Y/T DKO) and *P* > 0.9999 (all other comparisons).Representative immunofluorescence images showing Phalloidin‐labelled actin (red) and Hoechst (blue) in latrunculin B (LatB) (0.5 μM)‐ or cytochalasin D (CytD) (2 μM)‐treated HEK293A cells compared to control across Hippo pathway genome‐edited cells as shown. Scale bar = 20 μm.Average change in cell volume obtained by DHM in response to hydrostatic pressure with 2 μM cytochalasin D (CytD) treatment in WT, Y/T DKO and LATS1/2 DKO cells. Each dot represents a single cell and error bars represent mean ± 95% CI. Data pooled from four independent experiments. Kruskal–Wallis test with Dunn’s *post‐hoc*. **P = 0.0044 (WT con vs. CytD), *P = 0.0409 (Y/T DKO con vs. CytD), *P* > 0.9999 (LATS1/2 DKO con vs. CytD), ***P = 0.0003 (WT con vs. Y/T DKO con), ***P < 0.001 (WT con vs. Y/T DKO CytD) and ***P < 0.001 (Y/T DKO CytD vs. LATS1/2 DKO CytD).Fold difference in average change in cell volume in response to dynamic hydrostatic pressure from (H) normalized against untreated cells. Each dot represents a single cell and error bars are 95% CI. Mann–Whitney U test. **P = 0.0069 (WT vs. Y/T DKO) and **P = 0.0031 (WT vs. LATS1/2 DKO).Average change in cell volume in response to hydrostatic pressure with 0.5 μM latrunculin B (Lat B) treatment in WT, Y/T DKO and LATS1/2 DKO cells. Each dot represents a single cell and error bars represent mean ± 95% CI. Data pooled from four independent experiments. Kruskal–Wallis test with Dunn’s *post‐hoc*. ***P < 0.001 (all comparisons), *P* = 0.0972 (LATS1/2 DKO con vs. LatB) and *P = 0.0216 (Y/T DKO LatB vs. LATS1/2 DKO LatB).Fold difference in average change in cell volume in response to cyclic 0.1 Hz 100 mbar hydrostatic pressure from (F) normalized against control. Each dot represents a single cell and error bars represent mean ± 95% CI. Mann–Whitney U test. *P* = 0.1472 (WT vs. Y/T DKO) and **P = 0.0015 (WT vs. LATS1/2 DKO).

To gain further mechanistic insights into the cellular response to hydrostatic pressure, we set out to analyse the impact of disrupting the cytoskeleton using cytochalasin D (CytD) and sought to establish if the cellular volume changes upon actin disruption. CytD lowered the overall cell volume across genotypes (Fig 3A and B). This prompted us to examine if actin disruption changes the cellular response to cyclic fluid pressure. Initially, we examined by immunofluorescence that the established actin disruptors latrunculin B (LatB) and CytD work across the genotypes in a similar manner (Fig 3C). This was indeed the case, as the organization of phalloidin‐labelled actin filaments is severely disrupted in WT, Y/T DKO and LATS1/2 DKO cells (Fig 3C). We next confirmed that these chemicals inhibit YAP by treating cells with either LatB or CytD and examining the cell lysates on PhosTag gels followed by immunoblots. As expected (Yu *et al*, 2012; Zhao *et al*, 2012), we noticed an upshift of YAP upon LatB and CytD treatment (Fig EV2D). To establish if the difference in cell volume, we observed across genotypes (Fig EV2D–F), is conserved upon actin disruption, we compared the observed change in cellular volume to the overall effect CytD have on WT cells (Fig 3A and B). We find that the divergent cellular volume across genotypes is not dependent on a functional actin cytoskeleton (Fig 3A–C). Consequently, additional factors such as differentially expressed cytoskeletal factors, changes within the plasma membrane or cellular volume sensing (Fig EV1L and M) operating independently of the actin cytoskeleton are likely factors dictating this difference.

### Actin cytoskeletal dependence on the cellular response to oscillating fluid pressure

We next set out to establish if the actin cytoskeleton mediates the cellular response to fluid pressure via the Hippo pathway. First, we analysed WT cells treated with CytD or LatB under cyclic fluid pressure, which revealed that the cellular response to hydrostatic pressure in WT cells is strongly dependent on the actin cytoskeleton (Fig 3D and F). This actin dependence is conserved in Y/T DKO cells in contrast to LATS1/2 DKO cells. Cell volume response to hydrostatic pressure in LATS1/2 DKO cells is insensitive to LatB and CytD. This highlights that LATS1/2‐deficient cells have additional mechanisms to ensure cellular response to hydrostatic pressure (Fig 3D–G). Taken together, our data reveal that differences in cellular volume upon alterations in fluid pressure across genotypes are not solely due to genotype‐specific alterations causing modifications to the actin cytoskeleton. Consequently, additional factors operating independently of the actin cytoskeleton dictate this difference.

### Microtubule regulation of the cellular volume and response to oscillating fluid pressure

**Figure 4:**
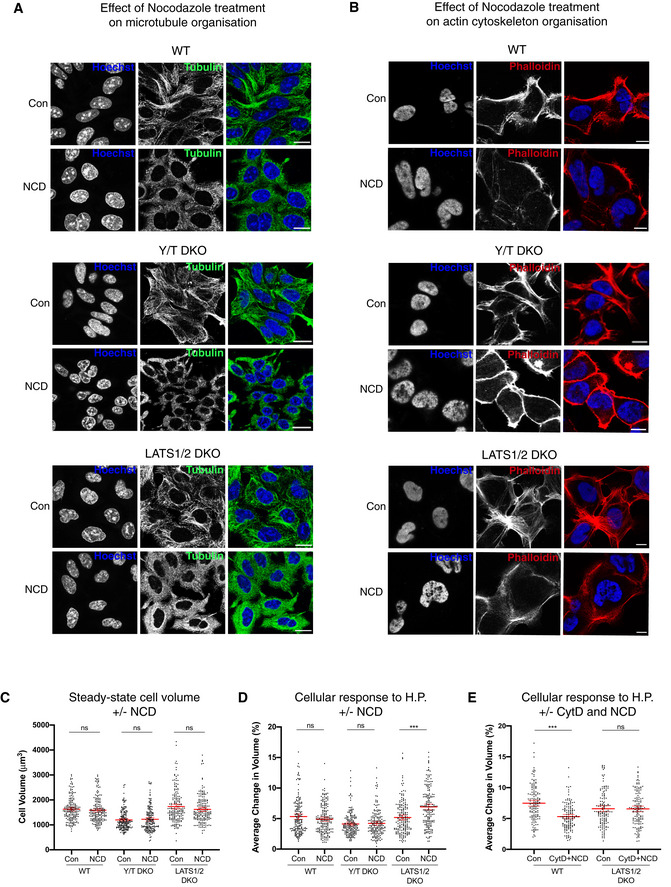
Disruption of microtubules has no effect on cellular response to hydrostatic pressure in WT and Y/T DKO cells Confocal IF images labelled with Hoechst (Blue) and alpha tubulin (green) showing loss of microtubule structures in response to 10 min nocodazole (NCD) treatment in WT, Y/T DKO and LATS1/2 DKO cells. Scale bar = 20 μm.Confocal IF images labelled with Hoechst (blue) and phalloidin (red) visualizing the actin cytoskeleton organization in response to NCD treatment. Scale bar = 10 μm.Steady‐state cell volume of nocodazole‐treated cells compared to control. Each dot represents a single cell and data pooled from five independent experiments. Error bars represent mean ± 95% CI. Mann–Whitney U test. *P* = 0.1925 (WT), *P* = 0.9008 (Y/T DKO) and *P* = 0.1375 (LATS1/2 DKO).Cell response to hydrostatic pressure after 10 min NCD treatment compared to control. Each dot represents a single cell and data pooled from five independent experiments. Error bars represent mean ± 95% CI. Mann–Whitney U test. *P* = 0.4240 (WT), *P* = 0.7567 (Y/T DKO) and ***P < 0.001 (LATS1/2 DKO).Cell response to hydrostatic pressure after CytD (40 min) and NCD (10 min) treatment compared to control. Each dot represents a single cell and data pooled from four independent experiments. Error bars represent mean ± 95% CI. Mann–Whitney U test. ***P < 0.001 (WT) and *P* > 0.9999 (LATS1/2 DKO).

**Figure EV3:**
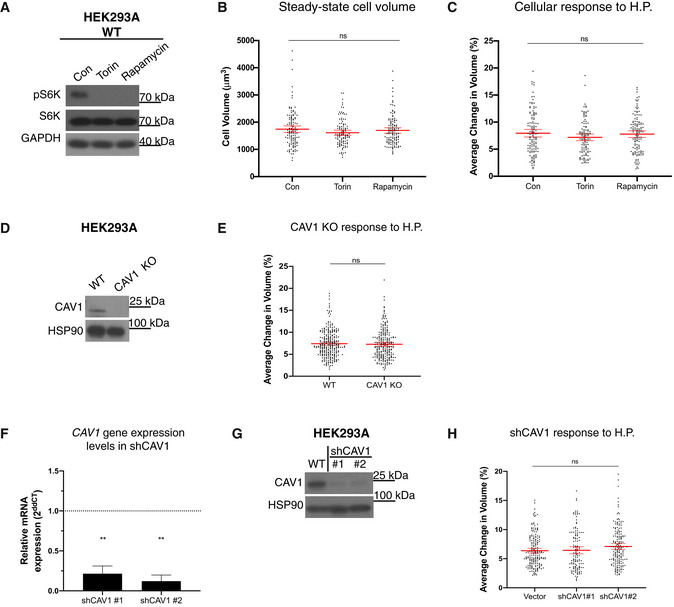
Additional mechanistic insights into cell volume responses mediated by hydrostatic pressure Lysates from cells treated for 40 min with Torin (1 μM) or Rapamycin (0.5 μM) compared to control cells were analysed by immunoblots for the levels of the mTORC1 substrate S6K, pS6K and GAPDH (loading control). Note the levels of pS6K are drastically decreased in Torin‐ and Rapamycin‐treated HEK293A cells, highlighting that these drugs effectively inhibits S6K phosphorylation.Cells treated with Torin (1 μM) and Rapamycin (0.5 μM) as in (A) and analysed by DHM to obtain their optical cellular volume. Each dot represents a single cell from three independent experiments. Forty minutes Torin or Rapamycin treatment does not affect steady‐state cell volume. Error bars represent mean ± 95% CI. Kruskal–Wallis test with Dunn’s *post‐hoc*. *P* = 0.5709 (con vs. Torin), *P* > 0.9999 (con vs. Rapamycin) and *P* > 0.9999 (Torin vs. Rapamycin).WT cells treated with Torin (1 μM) and Rapamycin (0.5 μM) as in (A) and imaged using DHM while being subjected to cyclic 0.1 Hz 100 mbar fluid pressure. Forty minutes Torin (1 μM) and Rapamycin (0.5 μM) treatment has no effect on cell volume changes in response to hydrostatic pressure. Each dot represents a single cell from three independent experiments. Error bars represent mean ± 95% CI. Kruskal–Wallis test with Dunn’s *post‐hoc*. *P* = 0.5321 (con vs. Torin), *P* > 0.9999 (con vs. Rapamycin) and *P* = 0.4860 (Torin vs. Rapamycin).Western blot confirming no CAV1 protein expression in CAV1 KO HEK293A cells.CAV1 KO cellular response to cyclic 0.1 Hz 200 mbar hydrostatic pressure compared to WT. Each dot represents a single cell from three independent experiments and error bars represent mean ± 95% CI. Mann–Whitney U test. *P* = 0.4904.Relative *CAV1* expression levels of CAV1 knockdown clones #1 and #2 from four independent experiments. Mann–Whitney U test. Error bars represent mean ± SD. **P = 0.0079.Western blot confirming reduction in total CAV1 protein levels in shCAV1 clones #1 and #2.shRNA CAV1 knockdown cell response to cyclic 0.1 Hz 100 mbar hydrostatic pressure compared to WT. Each dot represents a single cell and error bars represent mean ± 95% CI. Data pooled from four independent experiments. Kruskal–Wallis test with Dunn’s *post‐hoc*. *P* > 0.9999 (vector vs. shCAV#1), *P* = 0.1892 (vector shCAV#2) and *P* = 0.0812 (shCAV#1 vs. shCAV#2). Source data are available online for this figure.

Microtubules in some instances, like actin, function as mechanotransducers (Salmon, 1975; Rafiq *et al*, 2019). We therefore sought to establish the role of microtubules in the cellular response to cyclic hydrostatic pressure by treating cells with the microtubule depolymerization drug nocodazole (NCD). Initially, we established by immunofluorescence that the cellular microtubules are severely disrupted upon short‐term (10 min) NCD treatment across the genotypes (Fig 4A) and confirmed that the actin cytoskeleton is comparable across genotypes upon short‐term microtubule depolymerization (Fig 4B). When comparing the cellular effect upon microtubule depolymerization, the steady‐state cell volume across genotypes is conserved (Fig 4C), as well as a preserved cellular response towards hydrostatic pressure in Y/T DKO cells but not in LATS1/2 DKO cells. LATS1/2 DKO cells with disrupted microtubules (Fig 4A) respond to cyclic fluid pressure by an increased cellular volume change (Fig 4D). This prompted us to analyse the cellular response to hydrostatic pressure of combined actin cytoskeleton and microtubules disruption in WT and LATS1/2 DKO cells (Fig 4E). Combined NCD‐ and CytD‐treated WT cells experiencing cyclic fluid pressure phenocopied CytD‐treated WT cells (Figs 3D and 4E). NCD and CytD treatment of LATS1/2 DKO cells abolished the increased volume change observed in microtubule disrupted LATS1/2 DKO cells (Fig 4D and E), which highlights that this aspect is actin dependent. As mTORC1 is a major regulator of cell size (Liu & Sabatini, 2020), we asked if mTORC1 inhibition had consequences on cellular response to cyclic hydrostatic pressure. 40 min mTORC1 inhibition using two distinct and widely used mTORC1 inhibitors, namely the ATP‐competitive inhibitor Torin and the macrolide Rapamycin (Liu & Sabatini, 2020), do not change the cellular volume or cellular response to hydrostatic pressure (Fig EV3A–C). Overall, we conclude that the actin cytoskeleton mediates the cellular response to hydrostatic pressure.

### Clathrin‐dependent endocytosis mediates the cellular response to hydrostatic pressure

**Figure 5:**
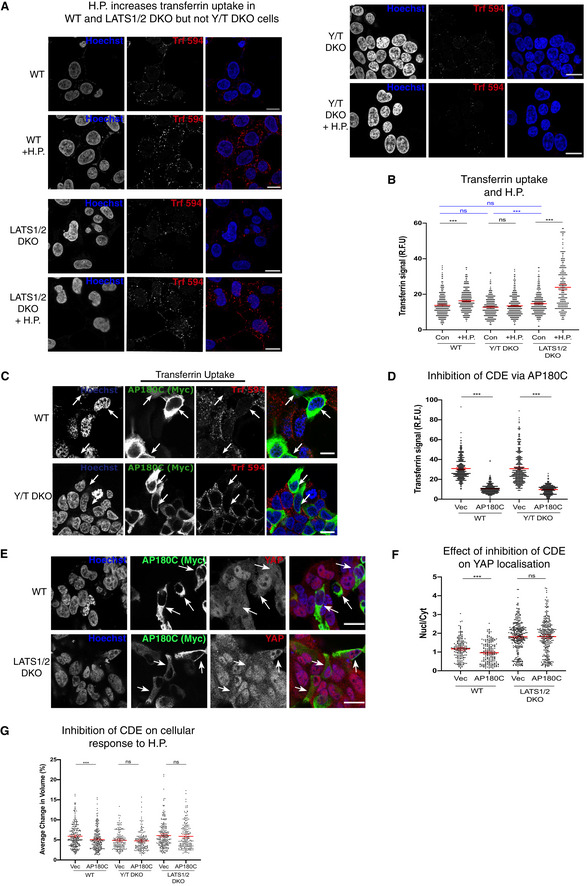
Hydrostatic pressure promotes clathrin‐dependent internalization Confocal IF images labelled with Hoechst (Blue) and internalized transferrin (Trf, red). Cells were allowed to internalize for 10 min, fixed and acid stripped to remove surface bound Trf. Hydrostatic pressure (H.P.) promotes transferrin uptake in WT and LATS1/2 DKO cells. Scale bar = 20 μm.Quantification of transferrin uptake in WT, Y/T DKO and LATS1/2 DKO cells treated with hydrostatic pressure compared to steady state. Each dot represents a single cell and error bars represent mean ± 95% CI. Data from four independent experiments. Kruskal–Wallis test with Dunn’s *post‐hoc*. Steady‐state statistical analysis is shown in blue and transferrin uptake induced by pressure is shown in black. ***P < 0.001 (WT con vs. +H.P.), *P* = 0.2375 (Y/T DKO con vs. +H.P.), ***P < 0.001 (LATS1/2 DKO con vs. +H.P.), *P* = 0.2764 (WT con vs. Y/T DKO con), *P* = 0.0808 (WT con vs. LATS1/2 DKO con) and ***P = 0.0004 (Y/T DKO con vs. LATS1/2 DKO con).Confocal IF images of myc‐tagged AP180C (green) and non‐transfected cells showing internalized Trf (red) and stained with Hoechst (blue). AP180C inhibits transferrin uptake in WT and Y/T DKO cells. Scale bar = 20 μm. Examples of AP180C expressing cells indicated with arrows.Quantification of transferrin uptake in AP180C positive cells compared to vector control from images as in C. Each dot represents a single cell from four independent experiments and error bars represent mean ± 95% CI. Mann–Whitney U test. ***P < 0.001 (for both comparisons).Confocal IF images of HEK293A WT cells showing subcellular localization of YAP (red) in AP180C (green) negative and positive WT and LATS1/2 DKO cells. Cells also stained with Hoechst (blue). Examples of AP180C expressing cells indicated with arrows.Quantification of cytoplasmic‐to‐nuclear ratio of YAP in AP180C positive WT and LATS1/2 DKO cells compared to vector control from images as in E. Each dot represents a single cell pooled from four independent experiments. Error bars represent mean ± 95% CI. Mann–Whitney U test. ***P = 0.0008 (WT) and *P* = 0.9318 (LATS1/2 DKO).Cell response to hydrostatic pressure with AP180C‐mediated inhibition of clathrin‐dependent endocytosis. Each dot represents a single cell pooled from four independent experiments. Error bars represent mean ± 95% CI. Data pooled from four independent experiments. Kruskal–Wallis test with Dunn’s *post‐hoc*. ***P = 0.0005 (WT), *P* = 0.3930 (Y/T DKO) and *P* = 0.2929 (LATS1/2 DKO).

**Figure EV4:**
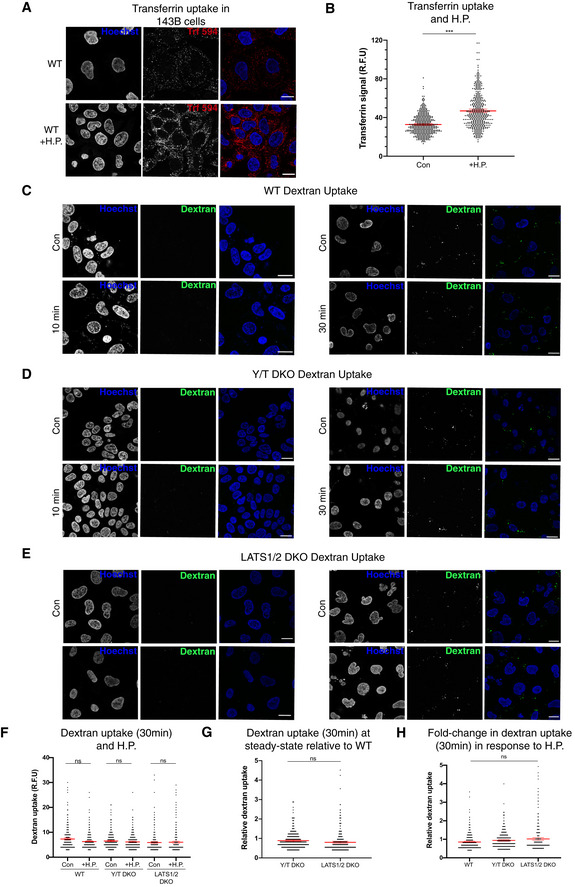
Endocytosis rates upon oscillating hydrostatic pressure Confocal image of fluorescently labelled transferrin (red) uptake by 143B cells under steady‐state conditions and in response to cyclic 0.1 Hz 200 mbar hydrostatic pressure. Cells labelled for Hoechst (blue). Scale bar = 20 μm.Quantification of transferrin uptake in 143B cells treated with hydrostatic pressure compared to steady state from images as in A. Each dot represents a single cell and error bars represent mean ± 95% CI. Data from four independent experiments. Mann–Whitney U test. *** *P* < 0.001.Confocal IF images of HEK293A cells. Cells are labelled with Dextran (green) and Hoechst (blue). HEK293A WT dextran uptake in response to 10 min (left) and 30 min (right) cyclic hydrostatic pressure compared to steady state (Con). Scale bar = 20 μm.Confocal images of Y/T DKO HEK293A cells. Cells are labelled with Dextran (green) and Hoechst (blue). Y/T DKO HEK293A dextran uptake in response to 10 min (left) and 30 min (right) cyclic hydrostatic pressure compared to steady state (Con). Scale bar = 20 μm.Confocal images of LATS1/2 DKO HEK293A cells. Cells are labelled with Dextran (green) and Hoechst (blue). LATS1/2 DKO HEK293A dextran uptake in response to 10 min (left) and 30 min (right) cyclic hydrostatic pressure compared to steady state (Con). Scale bar = 20 μm.Changes in Dextran signal (30 min uptake) in response to cyclic hydrostatic pressure compared to control in WT, Y/T DKO and LATS1/2 DKO. Data obtained from images as in A‐C. Each dot represents a single cell from three independent experiments. Error bars represent mean ± 95% CI. Data points obtained from images as in A‐C. Kruskal–Wallis test with Dunn’s *post‐hoc*. *P* = 0.1406 (WT), *P* = 0.6850 (Y/T DKO) and *P* > 0.9999 (LATS1/2 DKO).Dextran uptake (30 min) at steady state in Y/T DKO and LATS1/2 DKO cells relative to WT steady state. Each dot represents a single cell from three independent experiments. Data points obtained from images as in A‐C. Error bars represent mean ± 95% CI. Mann–Whitney U test. *P* > 0.9999.Dextran uptake (30 min) in response to hydrostatic pressure relative to steady state. Each dot represents a single cell from three independent experiments. Error bars represent mean ± 95% CI. Kruskal–Wallis test with Dunn’s *post‐hoc*. *P* = 0.0542 (WT vs. Y/T DKO), *P* = 0.8633 (WT vs. LATS1/2 DKO) and *P* = 0.5795 (Y/T DKO vs. LATS1/2 DKO).

**Figure EV5:**
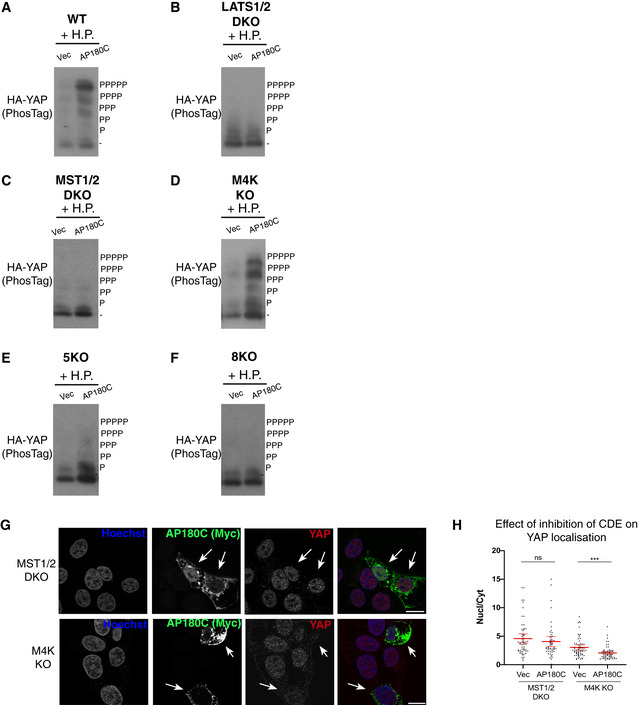
AP180C's effect on the Hippo pathway A–FPhosTag probing from HA‐tagged YAP levels in AP180C transfection positive cells. Vector or AP180C plasmid was co‐transfected with HA‐YAP and YAP activation status in response to hydrostatic pressure is shown (PhosTag gel). Anti‐HA tag antibody was used to probe for HA‐tagged YAP. YAP phosphorylation levels in HEK293A (A) WT, (B) LATS1/2 DKO, (C) MST1/2 DKO, (D) M4K4/6/7 KO (M4K KO), (E) MST1/2‐M4K4/6/7 KO (5KO) and (F) MST1/2‐M4K1/2/3/4/6/7 KO (8KO) cells are shown.GImmunofluorescence images show AP180C positive cells in green and YAP in red. Hoechst highlights the nucleus (shown in blue). AP180C promotes nuclear exclusion of YAP in M4K KO but not MST1/2 DKO cells. Cells were transfected with Myc‐tagged AP180C and the subcellular localization of YAP was examined in response to hydrostatic pressure. Scale bar = 20 μm.HQuantification of nuclear‐to‐cytoplasmic ratio (Nucl/Cyt) of YAP in AP180C transfection positive cells compared to control using images as shown in (G). Each dot represents a single cell and error bars are mean ± 95% CI. Graph shows data obtained from three independent experiments. Mann–Whitney U test. *P* = 0.3516 (MST1/2 DKO) and *P* = 0.0001 (M4K KO). Source data are available online for this figure.

As the cellular volume change is reversible and fast (within seconds) (Fig 2G), the surface area of the plasma membrane needs to dynamically and rapidly change. Taking a simplified view that the plasma membrane does not change area directly by stretching and that the cell is spherical, this 6.7% increase (Fig 2A–F) in cellular volume ((V_1_−V_o_)/V_o_) would equate to a change in plasma surface of 4.42% (S_1_−S_o_)/S_o_ = ((V_1_^2/3^−V_o_^2/3^)/V_o_^2/3^)). We and others recently discovered that caveolae, 60–100 nm invaginations of the plasma membrane composed of the structural caveolae proteins CAVEOLINs and CAVINs (Hansen & Nichols, 2010), mediate YAP/TAZ‐dependent cellular response to shear stress (Rausch *et al*, 2019) and substrate stiffness (Moreno‐Vicente *et al*, 2019) responses (Rausch & Hansen, 2020). We therefore examined if the cellular response to hydrostatic pressure is mediated via caveolae. However, cells devoid of the essential caveolar protein, CAVEOLIN1, are comparable to WT cells in their response to fluid pressure (Fig EV3D–H). An alternative way for the cell surface to undergo reversible change is through altering the rates of endocytosis (Kaksonen & Roux, 2018). Several types of endocytosis exist including macropinocytosis, caveolae‐mediated endocytosis, clathrin‐dependent endocytosis (CDE) and additional less well‐defined clathrin‐independent internalization processes (Hansen & Nichols, 2009; Kaksonen & Roux, 2018). Of these, CDE is the best characterized. In CDE, a range of adaptor proteins, including AP2, actin filaments and the ANTH domain containing protein AP180, are recruited to the plasma membrane and cause formation of clathrin coats (Pearse, 1976) in a precise and temporal manner (Ford *et al*, 2001; Taylor *et al*, 2011; Yoshida *et al*, 2018; Akamatsu *et al*, 2020), where after the coated vesicles with internalized cargo are pinched off by the recruitment of the large GTPase dynamin (Ford *et al*, 2001; Taylor *et al*, 2011; Yoshida *et al*, 2018; Akamatsu *et al*, 2020). Importantly, CDE is mechanosensitive (Boulant *et al*, 2011; Saleem *et al*, 2015; Ferguson *et al*, 2017; Malinverno *et al*, 2017; Akamatsu *et al*, 2020; Baschieri *et al*, 2020) and in non‐specialized cells account for upwards of 95% internalization of the plasma membrane (Bitsikas *et al*, 2014). CDE therefore represents the major route for internalization of the plasma membrane and the process is an attractive pathway that could confer the change in cell surface plasma membrane necessary for changing the cellular volume. To test this hypothesis, we used fluorescently labelled transferrin (Trf) that binds to the transferrin receptor and is internalized exclusively via clathrin‐mediated endocytosis (Ford *et al*, 2001; Taylor *et al*, 2011; Bitsikas *et al*, 2014; Yoshida *et al*, 2018). Using an established internalization assay, where surface bound Trf is stripped off the plasma membrane, we compared the internalization of Trf across WT, Y/T DKO and LATS1/2 DKO cells at steady state (Fig 5A and B). The internalization rate of Trf and thereby CDE at steady state in Y/T DKO is comparable to WT cells, whereas LATS1/2 DKO cells internalize Trf faster (Fig 5A and B). We next examined if hydrostatic pressure influences CDE under the same assay conditions used previously. We noticed a robust upregulation of Trf uptake upon cyclic hydrostatic pressure in both HEK293A and 143B cells (Figs 5A and B, and EV4A and B). This effect is conserved in LATS1/2 DKO cells but not in cells without YAP/TAZ (Fig 5A and B). Oscillating hydrostatic pressure therefore increases the rate of CDE in a YAP/TAZ‐dependent manner. The increased hydrostatic pressure‐mediated internalization rate is specific to CDE, as rates of fluid phase internalization as measured by uptake of fluorescently labelled dextran do not increase (Fig EV4C–H). To firmly establish the role of CDE in this process, we took advantage of a dominant negative CDE construct. This construct encodes the C terminus of the adaptor protein 180 (AP180C), and blocks clathrin‐mediated endocytosis (Ford *et al*, 2001). We initially expressed AP180C in WT and Y/T DKO cells to establish that AP180C function is YAP/TAZ independent. A robust decrease in Trf uptake in all AP180C expressing cells is, as expected, evident (Fig 5C and D). We next sought to analyse the effect of AP180C expression on YAP. We co‐labelled WT or LATS1/2 DKO cells expressing either vector or AP180C and analysed these by confocal microscopy. YAP localized predominantly in the cytoplasm in WT AP180C expressing cells, an effect not observed in LATS1/2 DKO cells (Fig 5E and F). YAP therefore gets inactivated upon CDE inhibition in a LATS1/2‐dependent manner. Our immunofluorescence‐based assay also allowed us to establish that our transfection efficiency is > 80%, which prompted us to analyse pools of cells expressing AP180C using DHM at the single cell level. In WT cells expressing AP180C, the cellular volume change upon cyclic fluid pressure is diminished to levels comparable to Y/T DKO cells (Figs 5G and EV2I), whereas AP180C had no effect on the cellular response to hydrostatic pressure in LATS1/2 DKO and Y/T DKO cells (Figs 5G and EV2I). LATS1/2 are activated by either MST1/2 (the *hpo* kinases), or by the MAP4K family members as alternative direct LATS1/2 activating kinases (Li *et al*, 2014b; Meng *et al*, 2015; Zheng *et al*, 2015). In order to define which parts of the upstream kinase module are critical for sensing and integrating the clathrin‐dependent endocytic cellular response to hydrostatic pressure to the Hippo pathway, we transfected vector control or AP180C into an expanded range of isogenic cell lines (Hansen *et al*, 2015b; Meng *et al*, 2015), including WT, LATS1/2 DKO, MST1/2 DKO, MAP4K4/6/7 KO, MST1/2‐MAP4K4/6/7 KO and MST1/2‐MAP4K1/2/3/4/6/7 KO cells. These transfected cells were stimulated with oscillating hydrostatic pressure and changes in YAP phosphorylation levels were analysed by PhosTag. A clear upshift (increased YAP phosphorylation) is observed in WT and MAP4K4/6/7 KO cells, but not in LATS1/2 DKO, MST1/2 DKO, MST1/2‐MAP4K4/6/7 KO or MST1/2‐MAP4K1/2/3/4/6/7 KO cells (Fig EV5A–F). These findings suggest that YAP is inhibited and cytosolic in AP180C MAP4K4/6/7 KO, but not in MST DKO cells stimulated with oscillating hydrostatic pressure. In order to confirm this, in complimentary experiments, we compared changes in the nuclear localization of YAP between AP180C transfection positive and transfection negative cells within the same field of view upon stimulation with cyclic hydrostatic pressure. A marked decrease in YAP nuclear localization was observed in MAP4K4/6/7 KO cells but not in MST1/2 KO cells (Fig EV5G and H), which highlights the role of MST1/2 in the integration of CDE with downstream cellular effects induced by hydrostatic pressure. We conclude that CDE is regulated by hydrostatic pressure in a YAP/TAZ‐dependent manner and function as a mediator of the cellular response to hydrostatic pressure.

Cell volume regulation at steady state and upon hydrostatic pressure differ in some aspects, but both regulations centre on the Hippo pathway transcriptional mediators YAP/TAZ. We here establish that YAP and TAZ regulate plasma membrane tension, and that YAP/TAZ are critical regulators of cell volume, and that hydrostatic pressure and CDE are additional factors that dynamically dictate this fundamental cell volume regulatory process. This intricate feedback underlies the cellular response to hydrostatic pressure.

## Discussion

Our studies reveal through quantitative single‐cell measurements that oscillating fluid pressure induces fast cell‐size fluctuations dependent on YAP/TAZ. Cells devoid of YAP/TAZ are smaller and have a lower membrane tension and are less adaptable to rapid cell shape changes. We show that this dynamic cellular response is contingent on both the cytoskeleton‐ and clathrin‐dependent endocytosis. YAP/TAZ are dephosphorylated and consequently activated upon elevated cyclic fluid pressure. This activation drives a transcriptional response, including of the matricellular proteins CYR61 and CTGF. CYR61 and CTGF have established roles in development, as well as in pro‐tumorigenic and regenerative properties within the microenvironment (Mo *et al*, 2002; Zhao *et al*, 2008; Mokalled *et al*, 2016; Park *et al*, 2019; Pepe‐Mooney *et al*, 2019). Such coupling as described here between the Hippo pathway, internalization of receptors, nutrients (including iron) and dynamic forces within the cellular microenvironment likely ensure cell‐size distribution homeostasis in a population by modulating growth rates and the duration of cell division cycles (Stewart *et al*, 2011; Cadart & Heald, 2019; Cadart *et al*, 2019). This feedback process involves dramatic and rapid changes in the cellular volume and consequently includes changes in intracellular concentrations of solutes and organelles, hence influence directly the rates of chemical reactions occurring in the cell (Cai *et al*, 2010; Miermont *et al*, 2013; Hansen *et al*, 2015b; Delarue *et al*, 2018; Ginzberg *et al*, 2018; Neurohr *et al*, 2019; Lu *et al*, 2020). Similarly, mechanisms ensure surface tension homeostasis (Lecuit & Lenne, 2007; Collinet & Lecuit, 2021), couple volume and surface area (Fischer‐Friedrich *et al*, 2014; Guo *et al*, 2017). Our data highlight that rapid volume modulation depends on YAP/TAZ and the actin cortex, and suggest that clathrin‐dependent endocytosis provides a membrane reservoir, which is coupled to membrane tension. This implies that repeated cycles of rapid internalization, endocytic recycling and exocytosis take place in order to replenish the plasma membrane. Long‐term hydrostatic pressure adaptations are likely caused by YAP/TAZ‐mediated transcription of regulators of ion and water fluxes (Figs 2P and EV1K–M). These examples hint at how dynamic size homeostasis is a consequence of intrinsic and extrinsic parameters that drive global growth, and that this needs to be precisely coupled by both biological and physical effects in a healthy individual (Cadart & Heald, 2019; Cadart *et al*, 2019). Specialized cell types differ greatly in size; however, the size of specific cell types within a given tissue is strikingly similar (Ginzberg *et al*, 2015; Cadart *et al*, 2019). Uniformity of the size of particular cell types is a consistent feature of healthy tissues. Consequently, loss of cell‐size uniformity is a frequent diagnostic marker of malignancy (Greenough, 1925; Nguyen *et al*, 2016; Dagogo‐Jack & Shaw, 2018; Gast *et al*, 2018). Cell volume is a fundamental aspect in biology and integrates both the physics and the physiology, including the metabolic state of the cell. Given that YAP/TAZ activation regulates cell growth and proliferation, we speculate that dysregulation of oscillating fluid pressure, likely coupled to the viscoelasticity of the ECM (Vining & Mooney, 2017; Zanconato *et al*, 2019; Chaudhuri *et al*, 2020), may therefore play a major role in a variety of conditions. For instance, the interplay among YAP/TAZ, oscillating fluid pressure and resistance to cancer treatments could be a confounding factor in solid tumours characterized by an elevated interstitial fluid pressure (Heldin *et al*, 2004; Moroishi *et al*, 2015a; Northcott *et al*, 2018; Stylianopoulos *et al*, 2018); and together with additional factors in the microenvironment drive cancer development and metastasis (Heldin *et al*, 2004; Moroishi *et al*, 2015a; Fulford *et al*, 2018; Northcott *et al*, 2018; Rognoni & Walko, 2019; Salem & Hansen, 2019; Zanconato *et al*, 2019; Chaudhuri *et al*, 2020; Thompson, 2020). The interstitial fluid pressure within the tumour microenvironment drastically increases as a consequence of tumour growth, increased vascular permeability and impaired lymphatic drainage (Northcott *et al*, 2018). Elevated pressure in solid tumours caused by increased IFP is one of the culprits that impede effective cancer treatment, as it drives cancer proliferation and metastasis, as well as make it challenging to deliver therapeutics to their targets (Heldin *et al*, 2004; Nathan *et al*, 2005, 2008; Matsubara *et al*, 2013; Ariffin *et al*, 2014; Jain *et al*, 2014; Holle *et al*, 2016). Our findings highlight that the cellular response to fluid pressure via the Hippo pathway is distinct to shear stress, as it is sensed and mediated differently at the plasma membrane. How short‐timescale size fluctuations connect to longer‐term growth and differentiation processes *in vivo* are still outstanding questions. We hypothesize that such couplings might provide robust feedback loops (Park & Hansen, 2021) into providing steady‐state cell‐size control important for organ homeostasis, which are likely important cellular regulators during development (Chan *et al*, 2019), in inflammatory and regenerative processes (Chan & Hiiragi, 2020), and that this dynamic regulation might be chronically altered in tumours (Heldin *et al*, 2004; Wiig & Swartz, 2012; Northcott *et al*, 2018). Our work highlights a highly dynamic cellular homeostasis module that is constantly at work in cells. We expect these dynamics are integrated into organ‐wide multilevel regulation with potential consequences on physiology and disease. How these dynamics are integrated among changes in hydrostatic pressure, CDE and the Hippo pathway is not yet fully understood. We speculate cellular sensing of membrane tension and overall cellular volume changes may alter protein condensate levels, and overall spatiotemporal localization of protein complexes, which together might play coordinating roles in this regulation. It is possible that both subcellular components, integral parts of the plasma membrane, and cell–cell and cell–matrix interactions might serve as integrators of this cellular process (Rausch & Hansen, 2020). Our findings offer therapeutically targetable insights into fundamental cellular processes and highlight the intricate dynamics necessary for adaptive cell‐size regulation within the microenvironment.

## Materials and Methods

### Reagents and Tools table

**Table:** 

Reagent/ resource	Reference or source	Identifier or catalogue number
**Experimental models**		
HEK293A cells (*H. sapiens*)	Prof. Kun‐Liang Guan, University of California San Diego, USA	
143B cells (*H. sapiens*)	Prof. Donald Salter, University of Edinburgh, UK	
**Recombinant DNA**		
AP180C‐MYC	Dr Ben Nichols, LMB, Cambridge, UK	
HA‐YAP	Prof Kun Liang Guan, UCSD	
pQCXIH‐Myc‐YAP	Addgene	#33091
pQCXIH‐Myc‐YAP S94A	Addgene	#33094
**Antibodies**		
Rabbit anti‐YAP1	Abcam	ab52771
Mouse anti‐YAP1/TAZ	Santa Cruz Biotechnology	sc101199
Rabbit anti‐ “active” YAP	Abcam	ab205270
Rabbit anti‐phospho YAP S127	Cell Signalling Technology	4911
Rabbit anti‐TAZ (V386)	Cell Signalling Technology	4883
Rabbit anti‐pan TEAD (D2F7L)	Cell Signalling Technology	13295S
Goat anti‐CTGF (C‐20)	Santa Cruz Biotechnology	SC14939
Rabbit anti‐CYR61	Santa Cruz Biotechnology	SC13100
Mouse anti‐CAV1	BD Biosciences	BD610060
Rabbit anti‐LATS1	Cell Signalling Technology	3477
Rabbit anti‐LATS2	Cell Signalling Technology	5888
Rabbit anti‐NF2	Cell Signalling Technology	D1D8
Rabbit anti‐GAPDH (FL‐335)	Santa Cruz Biotechnology	SC25778
Mouse anti‐HSP90	BD Biosciences	BD610418
Mouse anti‐HA‐Tag (6E2)	Cell Signalling Technology	2999S
Mouse anti‐α/β tubulin	Cell Signalling Technology	2148
Mouse anti‐MYC	Cell Signalling Technologies	2276S
Goat anti‐mouse IgG/HRP	DAKO	P044701
Goat anti‐rabbit IgG/HRP	DAKO	P044801
Rabbit anti‐goat IgG/HRP	DAKO	P044901
**Oligonucleotides and sequence‐based reagents**		
PCR primers	IDT	See Methods and Protocols section
**Chemicals, enzymes, and other reagents**		
μ‐Slide I luer 0.4 channels	ibidi	80176
μ‐Slide VI 0.4 channel slides	ibidi	80604
0.05% Trypsin‐EDTA	Thermo Fisher Scientific	25300054
Alexa Fluor 488 phalloidin	Thermo Fisher Scientific	A12379
Brilliant III Ultra‐Fast SYBR Green QPCR master mix	Agilent Technologies	600883
Cytochalasin D	Sigma‐Aldrich	22144‐77‐0
Dextran, Oregon Green 488; 70,000 MW	Life technologies	D7173
DMEM/F‐12	Thermo Fisher Scientific	21331046
DMSO	Sigma‐Aldrich	D2650
DPBS^−/−^	Thermo Fisher Scientific	14190144
DPBS^+/+^	Thermo Fisher Scientific	14040133
Immobilon western chemiluminescent HRP substrate	Millipore	WBKLS0500
Exoenzyme C3	Sigma‐Aldrich	341208
Flipper‐TR	Spirochrome	SC020
GenJet	SignaGen	
Goat anti‐mouse immunoglobulin Alexa Fluor 488	Thermo Fisher Scientific	A‐11029
Goat anti‐rabbit immunoglobulin Alexa Fluor 594	Thermo Fisher Scientific	A‐11037
Heat inactivated FBS	Thermo Fisher Scientific	10500064
High‐capacity cDNA Reverse transcription kit	Applied Biosystems	4374966
Hygromycin B	Scientific Laboratory Supplies	H5527‐250MG
Jasplakinolide	Invitrogen	J7473
Latrunculin B	Sigma‐Aldrich	L5288
L‐glutamine (200 mM)	Thermo Fisher Scientific	A2916801
LY 294002	Sigma‐Aldrich	L9908
Nocodazole	Sigma‐Aldrich	M1404
Paraformaldehyde	Thermo Fisher Scientific	28908
Penicillin‐Streptomycin (10,000 U/ml)	Thermo Fisher Scientific	15140122
PhosTag reagent	Wako Chemicals	AAL‐107
PVDF membrane	Merck	IPVH00010
Rapamycin	Sigma‐Aldrich	R0395
RNeasy plus micro kit	Qiagen	74034
Torin	Sigma‐Aldrich	SML1224
Transferrin (human) Alexa 594	Life Technologies	T13343
Y‐27632	Tocris	1254
**Software**		
GraphPad Prism 8.0	https://www.graphpad.com/scientific‐software/prism/	
MATLAB	https://www.mathworks.com/products/matlab.html	
ImageJ	https://imagej.nih.gov/ij/	
BioRender	https://biorender.com	

### Methods and Protocols

#### Cell culture

Cell lines were cultured in a humidified incubator at 37°C with 5% CO_2_ HEK293A and 143B cells were cultured in DMEM (Gibco) supplemented with 10% heat‐inactivated FBS (Gibco), 1% (v/v) penicillin–streptomycin (Gibco) and 2 mM L‐glutamine (Gibco) unless indicated otherwise. Cells were passaged before cells reached 70–80% confluence using 0.05% Trypsin‐EDTA (0.05%) (Gibco). HEK293A wild‐type, YAP/TAZ double‐knockout cells, LATS1/2 double‐knockout cells, NF2 knockout cells, TEAD1/2/4 triple‐knockout cells, MST1/2 double‐knockout cells and other HEK293A derived knockouts were obtained from Professor Kun‐Liang Guan’s lab at the University of California San Diego (UCSD) (Hansen *et al*, 2015b; Meng *et al*, 2015; Lin *et al*, 2017). 143B, osteosarcoma‐derived cells were obtained from Professor Donald Salter, University of Edinburgh (UoE).

#### Generation of knockout (KO) cell lines

Guide sequences for *YAP1* and *WWTR1* (encoding TAZ) were annealed to pSpCas9(BB)‐2A‐Puro (PX459 V2.0; Addgene plasmid #48139) and plasmids were generated using heat‐shocked DH5α‐competent *E. coli* as previously described (Hansen *et al*, 2015b; Rausch *et al*, 2019).

Genome‐edited 143B cells were generated in this study. 143B cells were transfected with plasmids using GenJet *in vitro* transfection reagent (SignaGen Laboratories) and cells were selected with puromycin (Alfa Aesar) 24 h post‐transfection for 2–3 days. Puromycin‐resistant cells were single‐cell sorted into 96‐well plates containing growth medium supplemented with 20% total serum concentration at the QMRI Flow Cytometry and Cell Sorting Facility (UoE). Replica plates were generated to allow for screening of KO clones by Western blotting. Confirmed knockout clones were expanded, analysed and frozen down.

#### Generation of YAP or TAZ re‐expression cell lines

Wild‐type YAP and S94A mutant YAP stable expression was achieved in HEK293A YAP/TAZ double‐knockout cells using retroviral transduction. Virus expressing pQCXIH‐Myc‐YAP or pQCXIH‐Myc‐YAP S94A plasmids were added to polybrene‐treated cells and selected using hygromycin B (SLS) for YAP re‐expressing cell lines. Virus expressing a TAZ WT construct (Zhang *et al*, 2009; Rausch *et al*, 2019) were added to polybrene‐treated Y/T DKO cells; cells were selected using puromycin to obtain stable‐cell populations expressing TAZ variants.

#### Transient transfection of dominant negative AP180 (AP180c)

AP180C is a MYC‐tagged carboxy‐terminal domain of AP180 and acts as a dominant negative mutant of AP180, which inhibits clathrin‐dependent endocytosis (Ford *et al*, 2001). HEK293A cells were seeded at 60–70% confluence in six‐well plates and were transiently transfected with 1 μg of AP180C plasmid using GenJet *in vitro* DNA transfection reagent (SignaGen Laboratories) following the manufacturer’s protocol. Experiments investigating the role of clathrin‐mediated endocytosis were carried out between 36 and 40 h post‐transfection.

#### Western blotting

Cells were harvested, and lysates prepared with lysis buffer consisting of pH 6.8 50 mM Tris buffer, 2% (w/v) sodium dodecyl sulphate, 0.025% (w/v) bromophenol blue, 40% (v/v) glycerol and 5% (v/v) β‐mercaptoethanol. Samples were run on SDS–PAGE gels via electrophoresis between 70 and 110 V. PhosTag Western blotting, which segregates proteins depending on the degree of phosphorylation, was carried out by supplementing standard 7.5% SDS–PAGE gels with 5 mM PhosTag reagent (Wako Chemicals) and 50 μM MnCl_2_. Segregated protein mixtures were then transferred to a PVDF. Enhanced chemiluminescence (Millipore) was used for immunodetection and signal was developed onto X‐ray films. Western blots shown are representative of 2–4 biological replicates.

Primary antibodies used in the Western blot analysis are as the following: anti‐YAP1 (ab52771) from abcam; anti‐YAP1 63.7 (sc101199), anti‐CTGF (sc14939), anti‐CYR61 (sc13100) and anti‐GAPDH (sc47724) from Santa Cruz Biotechnology; anti‐TAZ V387 (4883), anti‐pan TEAD (13295S) and anti‐pYAP S127 (4911) from Cell Signalling Technology; anti‐HSP90 (BD610418) from BD Bioscience. Antibodies were diluted in TBST with 5% BSA at 1:1,000 except anti‐GAPDH (1:4,000) and anti‐HSP90 (1:10,000).

Secondary antibodies anti‐mouse IgG/HRP (P044701), anti‐rabbit IgG/HRP (P044801) and anti‐goat IgG/HRP (P044901) from Dako were diluted 1:10,000 in 5% milk in TBST.

#### Quantitative reverse transcription

RNA was extracted from mammalian cells using RNeasy micro kit (Qiagen) following manufacturer’s instructions. cDNA synthesis was carried out using high‐capacity cDNA reverse transcriptase kit (Applied Biosystems). Real‐time PCR using 1 ng of cDNA/ sample using brilliant III Ultra‐Fast SYBR Green qPCR master mix (Agilent Technologies) was used to detect relative gene expression levels.

Primer sequences (5′ to 3′) are as the following:

*HPRT1* (fwd AGAATGTCTTGATTGTGGAAGA; rev ACCTTGACCATCTTTGGATTA)

*YAP1* (fwd CCAAGGCTTGACCCTCGTTTTG; rev TCGCATCTGTTGCTGCTGGTTG)

*WWTR1 (TAZ)* (fwd AATGGAGGGCCATATCATTCGAG; rev GTCCTGCGTTTTCTCCTGTATC)

*CYR61* (fwd AGCCTCGCATCCTATACAACC; rev TTCTTTCACAAGGCGGCACTC)

*CTGF* (fwd CCAATGACAACGCCTCCTG; rev TGGTGCAGCCAGAAAGCTC)

*CAV1* (fwd GCGACCCTAAACACCTCAAC; rev ATGCCGTCAAAACTGTGTGTC)

*LRRC8A* (fwd CCTGCCTTGTAAGTGGGTCAC; rev CACAGCGTCCACGTAGTTGTA)

*LRRC8B* (fwd CAGCAACTTTTGGCTTCACTAC; rev TGTTTGCCGGAATCTATGTCAG)

*LRRC8C* (fwd GGGATGTGTTTACCGATTACCTC; rev CTGCACTCTTTTCGGAAGGC)

*LRRC8E* (fwd CAAGCAGTTCACGGAACAGC; rev GGGCCTCTGATAAGTTCTCCTG)

*AQP6* (fwd GTCTTCGCTTCCACCGACAG; rev GCGGGCTGGATTCATGGAG)

*AQP11* (fwd GCTCAAAGCGGTCATCACAGA; rev GCCAGCAGGTGGATACGAAG)

*TRPV1* (fwd CAGGCTCTATGATCGCAGGAG; rev TTTGAACTCGTTGTCTGTGAGG)

*TRPM7* (fwd ACTGGAGGAGTAAACACAGGT; rev TGGAGCTATTCCGATAGTGCAA)

*PIEZO1* (fwd CCGCTCGTTTCCGAGTCAC; rev TGGTAGCAGTAGAGGCAGATG).

#### Microfluidic setup to alter cellular hydrostatic pressure

A bespoke setup was established consisting of 5% CO_2_/air supplied from a gas cylinder (BOC) to OB1 Microfluidic flow controller (Elveflow) to control the air pressure applied to the input of a μ‐Slide I luer 0.4 or μ‐Slide VI 0.4 channels (ibidi) containing cells and media, while the output is closed with a stopper. Particular attention was paid to leave no airspace between the stopper and the media. Cells were seeded at 22,000 cells per channel and maintained in 1% FBS complete DMEM throughout the duration of the experiments. Cyclic hydrostatic pressure at 0.1 Hz of 100 mbar or 200 mbar was applied to cells.

#### Transferrin uptake assay

Cells were seeded as described for hydrostatic pump experiments in μ‐Slide VI 0.4 channels (ibidi) and maintained at 37°C, 5% CO_2_, overnight. Cells were serum starved for 1 h then incubated on ice for 10 min in pre‐cooled 50 µg/ml transferrin‐Alexa 594 (Life Technologies). Transferrin uptake was stimulated by incubating cells at steady state with pre‐warmed 50 µg/ml transferrin‐Alexa 594 at 37°C or in conjunction with hydrostatic pressure. Surface labelling of transferrin was removed by washing cells with ice‐cold stripping buffer (29.2 g/l NaCl, 0.5% (v/v) acetic acid in distilled H_2_O) twice for 30–40 s.

#### Dextran uptake assay

Cells were seeded as described for hydrostatic pump experiments in μ‐Slide VI 0.4 channels (ibidi) and maintained at 37°C, 5% CO_2_, overnight. Cells were serum‐starved for 1 h then incubated on ice with pre‐cooled 100 µg/ml dextran Oregon green 488 (Life Technologies). Dextran uptake was stimulated using pre‐warmed dextran Oregon green 488 and 100 ng/ml human recombinant EGF (Gibco) in serum‐free DMEM for 10 or 30 min at 37°C in conjunction with hydrostatic pressure or at cells at steady state.

#### Digital holographic microscopy (DHM)

Aforementioned microfluidic set up was coupled to a Digital Holographic Microscope (Phi LAB, Holomonitor M4) to investigate the effect of hydrostatic pressure on cell response in real time. Time‐lapse imaging was acquired for 60 s at a rate of 1 Hz. Then, cells were segmented with the Hstudio Tracking software (Otsu’s thresholding) to extract for each cell in the field of view and for each time point quantitative cellular parameters. In particular, cell area and mean optical thickness were used to calculate the cellular volume considering a mean cellular index of 1.38 and a mean media refractive index of 1.34. A Matlab script was written to automate calculation of average cell volume at steady state and percentage average change in cell volume in response to hydrostatic pressure.

#### Flipper‐TR and FLIM imaging

Flipper‐TR probe was diluted to 2 μM in serum‐free DMEM and applied to cells 15 min prior to imaging. Fluorescent lifetime imaging microscope (FLIM) was used to excite cells at 485 nm and photons were collected through a 600/50 nm bandpass filter. Average fluorescent lifetime measurements were obtained by fitting photon histograms with a double exponential using the SymPhoTime software, which ensured that chi‐squared value was close to 1. The fluorescent lifetime measurements were used as a readout for membrane tension, where lifetime values range between 2.8 and 7.0 ns (Colom *et al*, 2018). FLIM imaging was conducted at the Edinburgh Super‐Resolution Imaging Consortium (ESRIC) facility branch at Herriot‐Watt University.

#### Drug treatments

Cells were treated with DMSO control or drugs diluted in DMSO at the following concentrations: cells were treated with latrunculin B (0.5, 2 μM) or cytochalasin D (0.5, 2 μM; Sigma) for 40 min prior to hydrostatic pressure experiments. Nocodazole (3 μM; abcam) treatment was carried out for 10 min. For cytochalasin D and nocodazole combination treatment, cells were treated with cytochalasin D for a total length of 40 min while nocodazole for a total length of 10 min at the aforementioned concentrations. For Torin and Rapamycin treatments, cells were treated at 1 and 0.5 μM, respectively, for a total of 40 min.

#### Immunofluorescence imaging (IF)

Cells plated on μ‐Slide VI 0.4 channels (ibidi) were in general fixed with pre‐warmed 37°C 4% paraformaldehyde (PFA) (Thermo Fisher Scientific) in PBS^+/+^ for 20 min. For visualization of tubulin, cells were fixed with ice‐cold MeOH, as this better preserves the microtule network. PFA (or MeOH) was removed and carefully rinsed with PBS^+/+^. IF blocking buffer (2.5% (v/v) FBS (Life Technologies) and 0.3% (v/v) Triton‐X‐100 in PBS^+/+^) was applied, followed by primary antibody incubation at room temperature (anti‐YAP1 (ab52771; abcam; 1:400), Alexa Fluor 488 phalloidin (A12379; Thermo Fisher Scientific; 1:1,000)) for 3 h. Cells were incubated with secondary anti‐rabbit Immunoglobulin Alexa Fluor 594 (A11037, Thermo Fisher Scientific, 1:400) for 1 h and nuclei was labelled with Hoechst (H3570; Thermo Fisher Scientific, 1:2,000). Labelled cells were imaged using a 63× oil objective on a Leica TCS SP8 MP confocal microscope.

Nuclear‐to‐cytoplasmic ratio of fluorescence intensity of immunofluorescence images was quantified in Fiji/ImageJ. The nucleus was visualized in the Hoechst channel, and a region of interest (ROI) was determined and used to measure fluorescence intensity in the channel of interest. The same method was carried out to calculate fluorescence intensity in the cytoplasm. For transferrin and dextran measurements, ROI in the cytoplasm was determined by referring to the Hoechst channel and channel of interest. Three ROIs were measured per cell. For all measurements and analysis, cells with multiple nuclei, apoptotic or undergoing mitosis were excluded.

#### Statistical analysis

Unless otherwise stated, three independent experiments were carried out for each experiment. For statistical tests, Mann–Whitney U test or Kruskal–Wallis test with Dunn’s *post‐hoc* was conducted. *P* = 0.05 was determined as the level of significance for statistical tests.

## Author contributions

**Jiwon Park:** Conceptualization; Data curation; Formal analysis; Validation; Investigation; Methodology; Writing—original draft; Writing—review & editing; Designed the study and planned experiments. Performed most of the experiments, wrote the Matlab script and analysed the data. Helped write the manuscript including preparing the figures. Writing had critical input from all the authors. **Siyang Jia:** Formal analysis; Investigation. Performed some of the revision experiments, including conducting Western blot based experiments to establish further mechanistic insights. **Donald Salter:** Resources; Supervision. **Pierre Bagnaninchi:** Conceptualization; Resources; Data curation; Formal analysis; Supervision; Validation; Investigation; Methodology; Project administration; Writing—review & editing; Designed and conceptualized the study. Planned and supervised the experiments. **Carsten G Hansen:** Conceptualization; Resources; Data curation; Formal analysis; Supervision; Funding acquisition; Investigation; Visualization; Methodology; Writing—original draft; Project administration; Writing—review & editing.

## Disclosure and competing interest statement

The authors declare that they have no conflict of interest.

## Supporting information



Expanded View Figures PDF

Source Data for Expanded View

Source Data for Figure 1

Source Data for Figure 2

## Data Availability

According to EMBO guidelines, no data that require deposition in a public database have been generated as part of this study.
