# Supplementary material for: The Hippo pathway drives the cellular response to hydrostatic pressure
Source: EMBO J. 2022 Jun 15;41(13):e108719. doi: 10.15252/embj.2021108719 (PMC9251841; doi:10.15252/embj.2021108719)
Supplement: Supplementary file 1 — Expanded View Figures PDF [file EMBJ-41-e108719-s003.pdf]

Expanded View Figures

Figure EV1. YAP/TAZ regulate cell responses to hydrostatic pressure.

A PhosTag-based Western blot probed for YAP reveals no changes in YAP phosphorylation levels in HEK293A and 143B cells in response to static 500 mbar hydrostatic pressure (+) compared to control (−).
B Western blot showing LATS1 and LATS2 levels in WT and LATS1/2 DKO cells.
C Western blot showing NF2 expression level in WT and NF2 KO cells.
D Western blot showing TEAD expression level in WT and TEAD KO cells.
E Western blot showing YAP, TAZ and CYR61 levels in 143B YAP KO and TAZ KO cells relative to WT.
F 143B WT, YAP KO and TAZ KO response to 100 mbar cyclic hydrostatic pressure as measured by DHM. Each dot represents a single cell from three independent experiments and error bars represent mean ± 95% CI. Mann–Whitney U test. ***$P < 0.001$ (WT vs. YAP KO), ***$P = 0.006$ (WT vs. TAZ KO).
G Steady-state volume of Y/T DKO relative to WT YAP and S94A mutant YAP re-expressing Y/T DKO cells as measured by DHM. Each dot represents a single cell from three independent experiments and error bars represent 95% CI. Kruskal–Wallis test with Dunn's *post-hoc*. $P > 0.999$ (vector vs. WT YAP), $P = 0.2173$ (vector vs. S94A YAP) and, $P = 0.9837$ (WT YAP vs. S94A YAP).
H Western blot showing total YAP, TAZ and CYR61 levels in WT TAZ re-expressing cells compared to WT and Y/T DKO vector control.
I Steady-state cell volume WT TAZ re-expressing cells relative to Y/T DKO as imaged and analysed by DHM. Each dot represents a single cell from three independent experiments. Error bars represent mean ± 95% CI. Mann–Whitney U test. ***$P < 0.001$.
J Average change in cell volume in response to hydrostatic pressure in WT TAZ re-expressing cells relative to Y/T DKO as imaged and analysed by DHM. Each dot represents a single cell from three independent experiments. Error bars represent mean ± 95% CI. Mann–Whitney U test. ***$P < 0.001$.
K Candidate genes conferring YAP/TAZ-mediated cellular response to hydrostatic pressure. Gene expression levels of HEK293A WT cells in response to 0.1 Hz, 200 mbar cyclic hydrostatic pressure (4 h) compared with steady-state levels within each genotype and analysed by RT–qPCR. Graphs show data obtained from four independent experiments. Error bars represent mean ± SD. Kruskal–Wallis test with Dunn's *post-hoc*.
L Gene expression level induced by hydrostatic pressure in Y/T DKO#1 cells normalized to WT levels. Graphs show data obtained from four independent experiments. Error bars represent mean ± SD. Kruskal–Wallis test with Dunn's *post-hoc*.
M Gene expression level induced by hydrostatic pressure in Y/T DKO#2 cells normalized to WT levels. Graphs show data obtained from four independent experiments. Error bars represent mean ± SD. Kruskal–Wallis test with Dunn's *post-hoc*.

Source data are available online for this figure.

 © 2022 The Authors

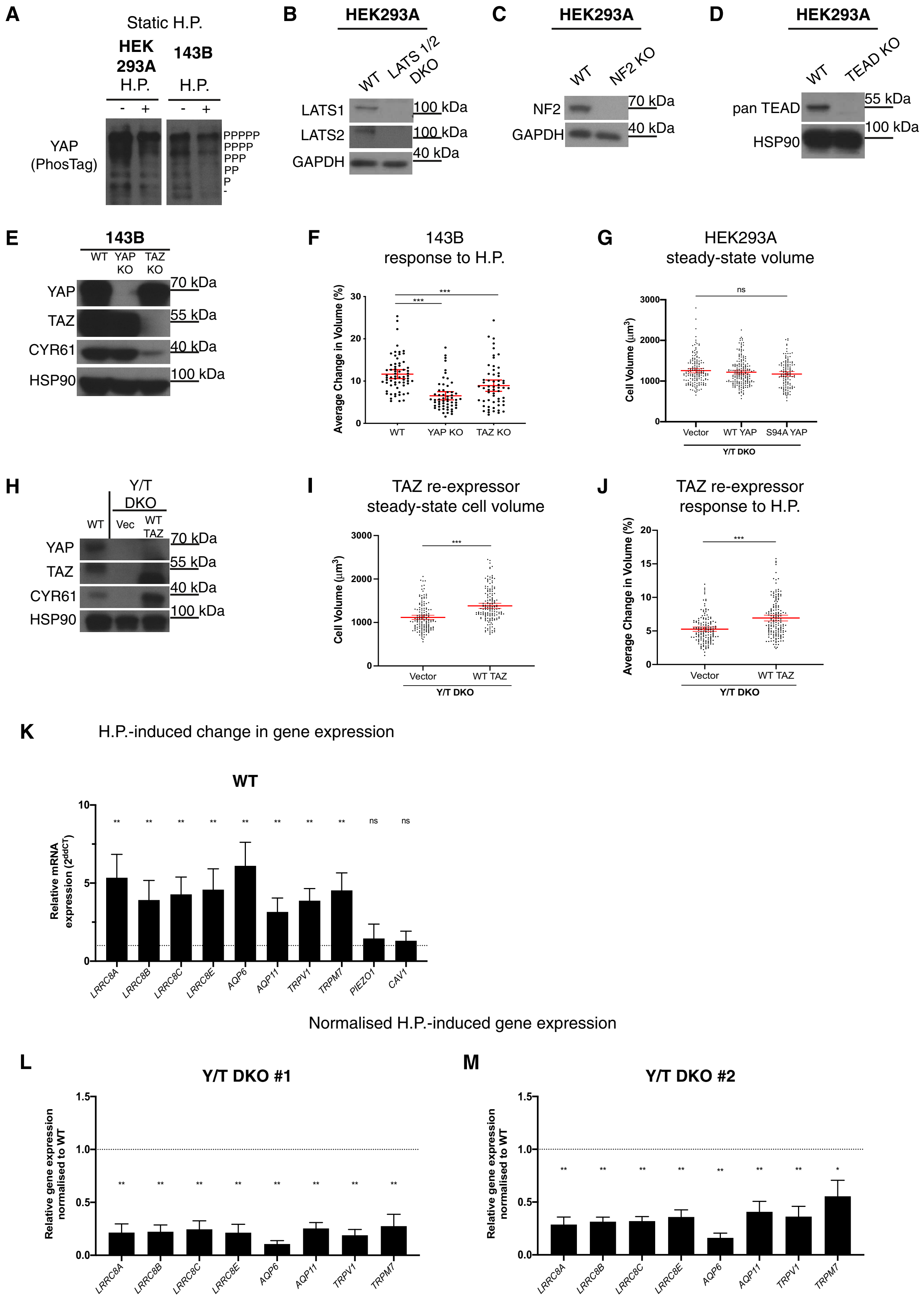
A
Static H.P.
HEK 293A
H.P.
143B
H.P.
- +
- +
YAP (PhosTag)
PPPPP
PPPP
PPP
PP
P
-
B
HEK293A
WT
LATS 1/2 DKO
LATS1
LATS2
GAPDH
100 kDa
100 kDa
40 kDa
C
HEK293A
WT
NF2 KO
NF2
GAPDH
70 kDa
40 kDa
D
HEK293A
WT
TEAD KO
pan TEAD
HSP90
55 kDa
100 kDa
E
143B
WT
YAP KO
TAZ KO
YAP
TAZ
CYR61
HSP90
70 kDa
55 kDa
40 kDa
100 kDa
F
143B response to H.P.
Average Change in Volume (%)
WT
YAP KO
TAZ KO
***
***
G
HEK293A steady-state volume
Cell Volume (μm³)
Vector
WT YAP
S94A YAP
Y/T DKO
ns
H
Y/T DKO
WT
Vec
WT TAZ
YAP
TAZ
CYR61
HSP90
70 kDa
55 kDa
40 kDa
100 kDa
I
TAZ re-expressor steady-state cell volume
Cell Volume (μm³)
Vector
WT TAZ
Y/T DKO
***
J
TAZ re-expressor response to H.P.
Average Change in Volume (%)
Vector
WT TAZ
Y/T DKO
***
K
H.P.-induced change in gene expression
WT
Relative mRNA expression (2^ddCT)
** ** ** ** ** ** ** ** ns ns
LRRC8A
LRRC8B
LRRC8C
LRRC8E
AQP6
AQP11
TRPV1
TRPM7
PIEZO1
CAV1
Normalised H.P.-induced gene expression
L
Y/T DKO #1
Relative gene expression normalised to WT
** ** ** ** ** ** ** **
LRRC8A
LRRC8B
LRRC8C
LRRC8E
AQP6
AQP11
TRPV1
TRPM7
M
Y/T DKO #2
Relative gene expression normalised to WT
** ** ** ** ** ** ** *
LRRC8A
LRRC8B
LRRC8C
LRRC8E
AQP6
AQP11
TRPV1
TRPM7

Figure EV1.

Figure EV2. Cytoskeletal implications in the cellular response to hydrostatic pressure.

A Average change in cell volume in response to 100 mbar cyclic hydrostatic pressure in WT and LATS1/2 DKO clones. Each dot represents a single cell. Data pooled from three independent experiments. Error bars represent mean ± 95% CI. Kruskal–Wallis test with Dunn's *post-hoc*. $P > 0.9999$ (for all comparisons).

B Average change in cell volume in response to 100 mbar cyclic hydrostatic pressure in WT and MST1/2 DKO. Each dot represents a single cell. Data pooled from three independent experiments. Error bars represent mean ± 95% CI. Mann–Whitney U test. $P = 0.0781$.

C HEK293A NF2 KO steady-state volume compared to WT cells. Each dot represents a single cell and Error bars represent mean ± 95% CI. Data pooled from three independent experiments. Mann–Whitney U test. ***$P < 0.001$.

D PhosTag-based Western blot probing for YAP reveals dephosphorylation of YAP in response to 0.5 μM latrunculin B (LB) and 2 μM cytochalasin D (CD) treatment in WT cells.

E Comparison of average change in cell volume measured by DHM in response to 100 mbar cyclic hydrostatic pressure with 2 μM latrunculin B treatment in WT, Y/T DKO and LATS1/2 DKO cells. Each cell represents a single cell and error bars represent mean ± 95% CI. Data pooled from four independent experiments. Mann–Whitney U test. ***$P < 0.001$ (WT), ***$P < 0.001$ (Y/T DKO) and *$P = 0.0331$ (LATS1/2 DKO).

F Comparison of average change in cell volume in response to 100 mbar cyclic hydrostatic pressure with 0.5 μM cytochalasin D treatment in WT, Y/T DKO and LATS1/2 DKO cells. Each cell represents a single cell and error bars represent mean ± 95% CI. Data pooled from four independent experiments. Mann–Whitney U test. $P = 0.2021$ (WT), **$P = 0.0035$ (Y/T DKO) and $P = 0.9148$ (LATS1/2 DKO).

G Fold difference in average change in cell volume in response to hydrostatic pressure from B normalized against cells not treated with 2 μM latrunculin B. Each dot represents a single cell and error bars are 95% CI. Mann–Whitney U test. $P > 0.9999$ (WT vs. Y/T DKO) and $P = 0.0723$ (WT vs. LATS1/2 DKO).

H Fold difference in average change in cell volume in response to hydrostatic pressure from C normalized against cells not treated with 0.5 μM cytochalasin D. Each dot represents a single cell and error bars represent mean ± 95% CI. Mann–Whitney U test. $P = 0.4619$ (WT vs. Y/T DKO) and $P = 0.7087$ (WT vs. LATS1/2 DKO).

I HEK293A YAP/TAZ DKO and LATS1/2 DKO clone #2's responses to cyclic 0.1 Hz 200 mbar dynamic hydrostatic pressure with AP180C-mediated inhibition of clathrin-dependent endocytosis. Each dot represents a single cell and error bars represent mean ± 95% CI. Data pooled from four independent experiments. Kruskal–Wallis test with Dunn's *post-hoc*. ***$P < 0.001$ (WT), $P = 0.2522$ (Y/T DKO) and $P = 0.0612$ (LATS1/2 DKO).

Source data are available online for this figure.

 © 2022 The Authors

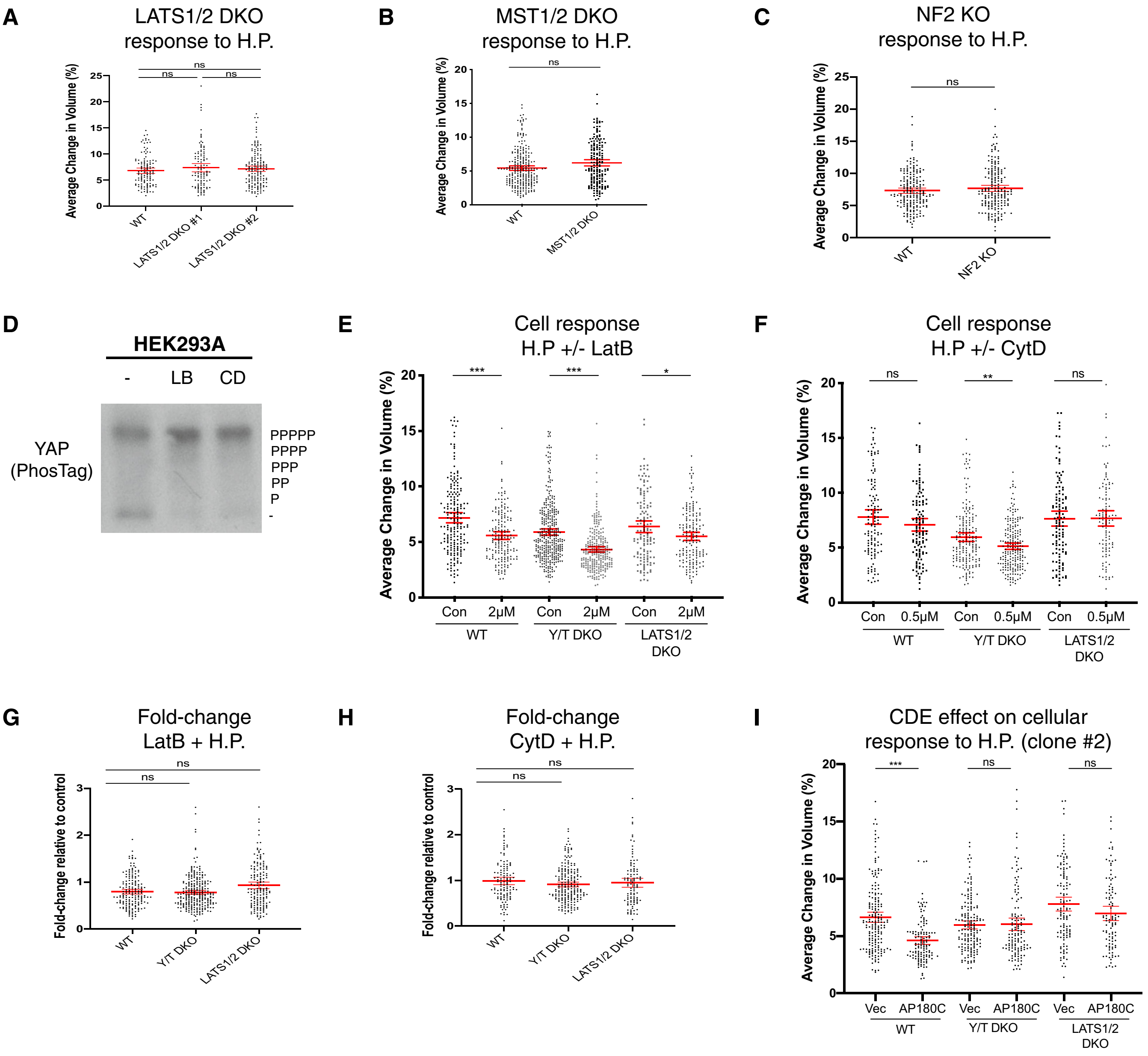
A
LATS1/2 DKO
response to H.P.
Average Change in Volume (%)
WT
LATS1/2 DKO #1
LATS1/2 DKO #2
ns
B
MST1/2 DKO
response to H.P.
MST1/2 DKO
C
NF2 KO
response to H.P.
NF2 KO
D
HEK293A
- LB CD
YAP
(PhosTag)
PPPPP
PPPP
PPP
PP
P
E
Cell response
H.P +/- LatB
Con 2μM
Y/T DKO
LATS1/2
DKO
F
Cell response
H.P +/- CytD
Con 0.5μM
G
Fold-change
LatB + H.P.
Fold-change relative to control
H
Fold-change
CytD + H.P.
I
CDE effect on cellular
response to H.P. (clone #2)
Vec AP180C

Figure EV2.

Figure EV3. Additional mechanistic insights into cell volume responses mediated by hydrostatic pressure.

A Lysates from cells treated for 40 min with Torin (1 μM) or Rapamycin (0.5 μM) compared to control cells were analysed by immunoblots for the levels of the mTORC1 substrate S6K, pS6K and GAPDH (loading control). Note the levels of pS6K are drastically decreased in Torin- and Rapamycin-treated HEK293A cells, highlighting that these drugs effectively inhibits S6K phosphorylation.

B Cells treated with Torin (1 μM) and Rapamycin (0.5 μM) as in (A) and analysed by DHM to obtain their optical cellular volume. Each dot represents a single cell from three independent experiments. Forty minutes Torin or Rapamycin treatment does not affect steady-state cell volume. Error bars represent mean ± 95% CI. Kruskal–Wallis test with Dunn's *post-hoc*. $P = 0.5709$ (con vs. Torin), $P > 0.9999$ (con vs. Rapamycin) and $P > 0.9999$ (Torin vs. Rapamycin).

C WT cells treated with Torin (1 μM) and Rapamycin (0.5 μM) as in (A) and imaged using DHM while being subjected to cyclic 0.1 Hz 100 mbar fluid pressure. Forty minutes Torin (1 μM) and Rapamycin (0.5 μM) treatment has no effect on cell volume changes in response to hydrostatic pressure. Each dot represents a single cell from three independent experiments. Error bars represent mean ± 95% CI. Kruskal–Wallis test with Dunn's *post-hoc*. $P = 0.5321$ (con vs. Torin), $P > 0.9999$ (con vs. Rapamycin) and $P = 0.4860$ (Torin vs. Rapamycin).

D Western blot confirming no CAV1 protein expression in CAV1 KO HEK293A cells.

E CAV1 KO cellular response to cyclic 0.1 Hz 200 mbar hydrostatic pressure compared to WT. Each dot represents a single cell from three independent experiments and error bars represent mean ± 95% CI. Mann–Whitney U test. $P = 0.4904$.

F Relative *CAV1* expression levels of CAV1 knockdown clones #1 and #2 from four independent experiments. Mann–Whitney U test. Error bars represent mean ± SD. $^{**}P = 0.0079$.

G Western blot confirming reduction in total CAV1 protein levels in shCAV1 clones #1 and #2.

H shRNA CAV1 knockdown cell response to cyclic 0.1 Hz 100 mbar hydrostatic pressure compared to WT. Each dot represents a single cell and error bars represent mean ± 95% CI. Data pooled from four independent experiments. Kruskal–Wallis test with Dunn's *post-hoc*. $P > 0.9999$ (vector vs. shCAV#1), $P = 0.1892$ (vector shCAV#2) and $P = 0.0812$ (shCAV#1 vs. shCAV#2).

Source data are available online for this figure.

 © 2022 The Authors

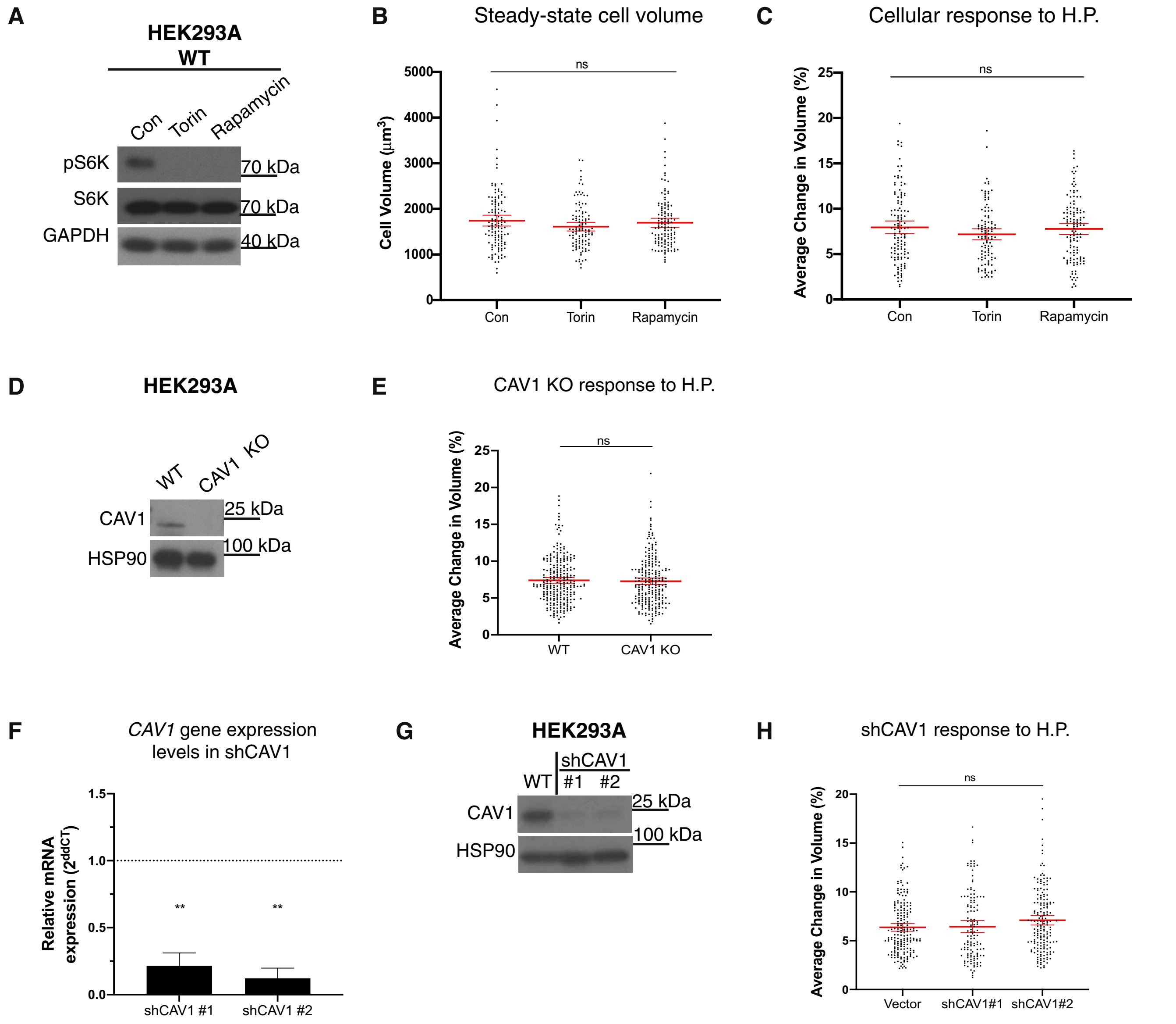
A
HEK293A
WT
Con
Torin
Rapamycin
pS6K
S6K
GAPDH
70 kDa
70 kDa
40 kDa
B
Steady-state cell volume
ns
Cell Volume (μm³)
Con
Torin
Rapamycin
C
Cellular response to H.P.
ns
Average Change in Volume (%)
Con
Torin
Rapamycin
D
HEK293A
WT
CAV1 KO
CAV1
HSP90
25 kDa
100 kDa
E
CAV1 KO response to H.P.
ns
Average Change in Volume (%)
WT
CAV1 KO
F
CAV1 gene expression levels in shCAV1
Relative mRNA expression (2ddCT)
**
**
shCAV1 #1
shCAV1 #2
G
HEK293A
shCAV1
WT
#1
#2
CAV1
HSP90
25 kDa
100 kDa
H
shCAV1 response to H.P.
ns
Average Change in Volume (%)
Vector
shCAV1#1
shCAV1#2

Figure EV3.

Figure EV4. Endocytosis rates upon oscillating hydrostatic pressure.

A Confocal image of fluorescently labelled transferrin (red) uptake by 143B cells under steady-state conditions and in response to cyclic 0.1 Hz 200 mbar hydrostatic pressure. Cells labelled for Hoechst (blue). Scale bar = 20 μm.

B Quantification of transferrin uptake in 143B cells treated with hydrostatic pressure compared to steady state from images as in A. Each dot represents a single cell and error bars represent mean ± 95% CI. Data from four independent experiments. Mann–Whitney U test. *** $P < 0.001$.

C Confocal IF images of HEK293A cells. Cells are labelled with Dextran (green) and Hoechst (blue). HEK293A WT dextran uptake in response to 10 min (left) and 30 min (right) cyclic hydrostatic pressure compared to steady state (Con). Scale bar = 20 μm.

D Confocal images of Y/T DKO HEK293A cells. Cells are labelled with Dextran (green) and Hoechst (blue). Y/T DKO HEK293A dextran uptake in response to 10 min (left) and 30 min (right) cyclic hydrostatic pressure compared to steady state (Con). Scale bar = 20 μm.

E Confocal images of LATS1/2 DKO HEK293A cells. Cells are labelled with Dextran (green) and Hoechst (blue). LATS1/2 DKO HEK293A dextran uptake in response to 10 min (left) and 30 min (right) cyclic hydrostatic pressure compared to steady state (Con). Scale bar = 20 μm.

F Changes in Dextran signal (30 min uptake) in response to cyclic hydrostatic pressure compared to control in WT, Y/T DKO and LATS1/2 DKO. Data obtained from images as in A-C. Each dot represents a single cell from three independent experiments. Error bars represent mean ± 95% CI. Data points obtained from images as in A-C. Kruskal–Wallis test with Dunn's *post-hoc*. $P = 0.1406$ (WT), $P = 0.6850$ (Y/T DKO) and $P > 0.9999$ (LATS1/2 DKO).

G Dextran uptake (30 min) at steady state in Y/T DKO and LATS1/2 DKO cells relative to WT steady state. Each dot represents a single cell from three independent experiments. Data points obtained from images as in A-C. Error bars represent mean ± 95% CI. Mann–Whitney U test. $P > 0.9999$.

H Dextran uptake (30 min) in response to hydrostatic pressure relative to steady state. Each dot represents a single cell from three independent experiments. Error bars represent mean ± 95% CI. Kruskal–Wallis test with Dunn's *post-hoc*. $P = 0.0542$ (WT vs. Y/T DKO), $P = 0.8633$ (WT vs. LATS1/2 DKO) and $P = 0.5795$ (Y/T DKO vs. LATS1/2 DKO).

 © 2022 The Authors

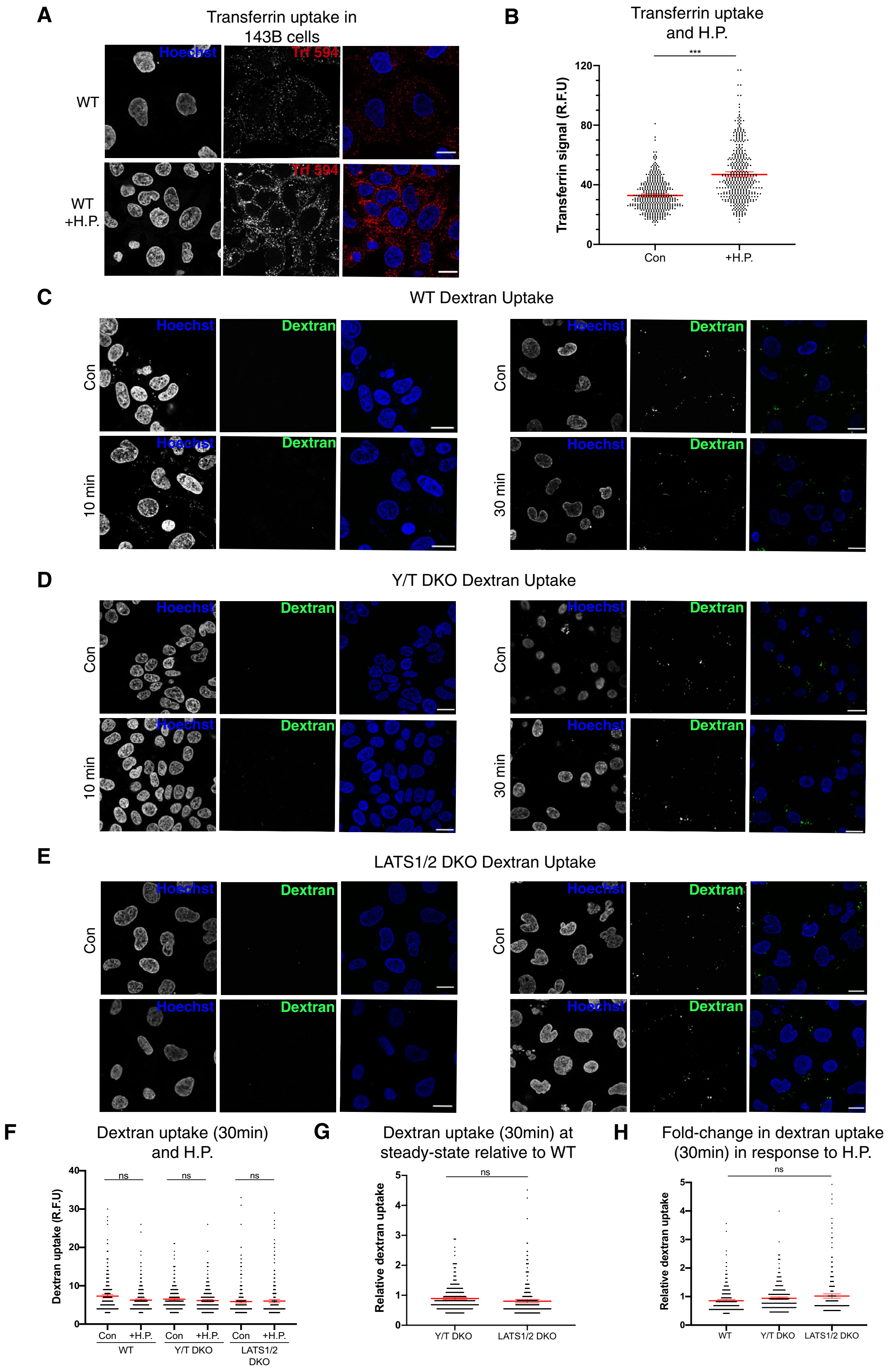

A
Transferrin uptake in 143B cells
Hoechst
Trf 594
WT
WT +H.P.
B
Transferrin uptake and H.P.
***
Transferrin signal (R.F.U)
120
80
40
0
Con
+H.P.
C
WT Dextran Uptake
Hoechst
Dextran
Con
10 min
30 min
D
Y/T DKO Dextran Uptake
E
LATS1/2 DKO Dextran Uptake
F
Dextran uptake (30min) and H.P.
Dextran uptake (R.F.U)
ns
WT
Y/T DKO
LATS1/2 DKO
G
Dextran uptake (30min) at steady-state relative to WT
Relative dextran uptake
H
Fold-change in dextran uptake (30min) in response to H.P.

Figure EV4.

Figure EV5. AP180C's effect on the Hippo pathway.

A–F PhosTag probing from HA-tagged YAP levels in AP180C transfection positive cells. Vector or AP180C plasmid was co-transfected with HA-YAP and YAP activation status in response to hydrostatic pressure is shown (PhosTag gel). Anti-HA tag antibody was used to probe for HA-tagged YAP. YAP phosphorylation levels in HEK293A (A) WT, (B) LATS1/2 DKO, (C) MST1/2 DKO, (D) M4K4/6/7 KO (M4K KO), (E) MST1/2-M4K4/6/7 KO (5KO) and (F) MST1/2-M4K1/2/3/4/6/7 KO (8KO) cells are shown.

G Immunofluorescence images show AP180C positive cells in green and YAP in red. Hoechst highlights the nucleus (shown in blue). AP180C promotes nuclear exclusion of YAP in M4K KO but not MST1/2 DKO cells. Cells were transfected with Myc-tagged AP180C and the subcellular localization of YAP was examined in response to hydrostatic pressure. Scale bar = 20 μm.

H Quantification of nuclear-to-cytoplasmic ratio (Nucl/Cyt) of YAP in AP180C transfection positive cells compared to control using images as shown in (G). Each dot represents a single cell and error bars are mean ± 95% CI. Graph shows data obtained from three independent experiments. Mann–Whitney U test. $P = 0.3516$ (MST1/2 DKO) and $P = 0.0001$ (M4K KO).

Source data are available online for this figure.

 © 2022 The Authors

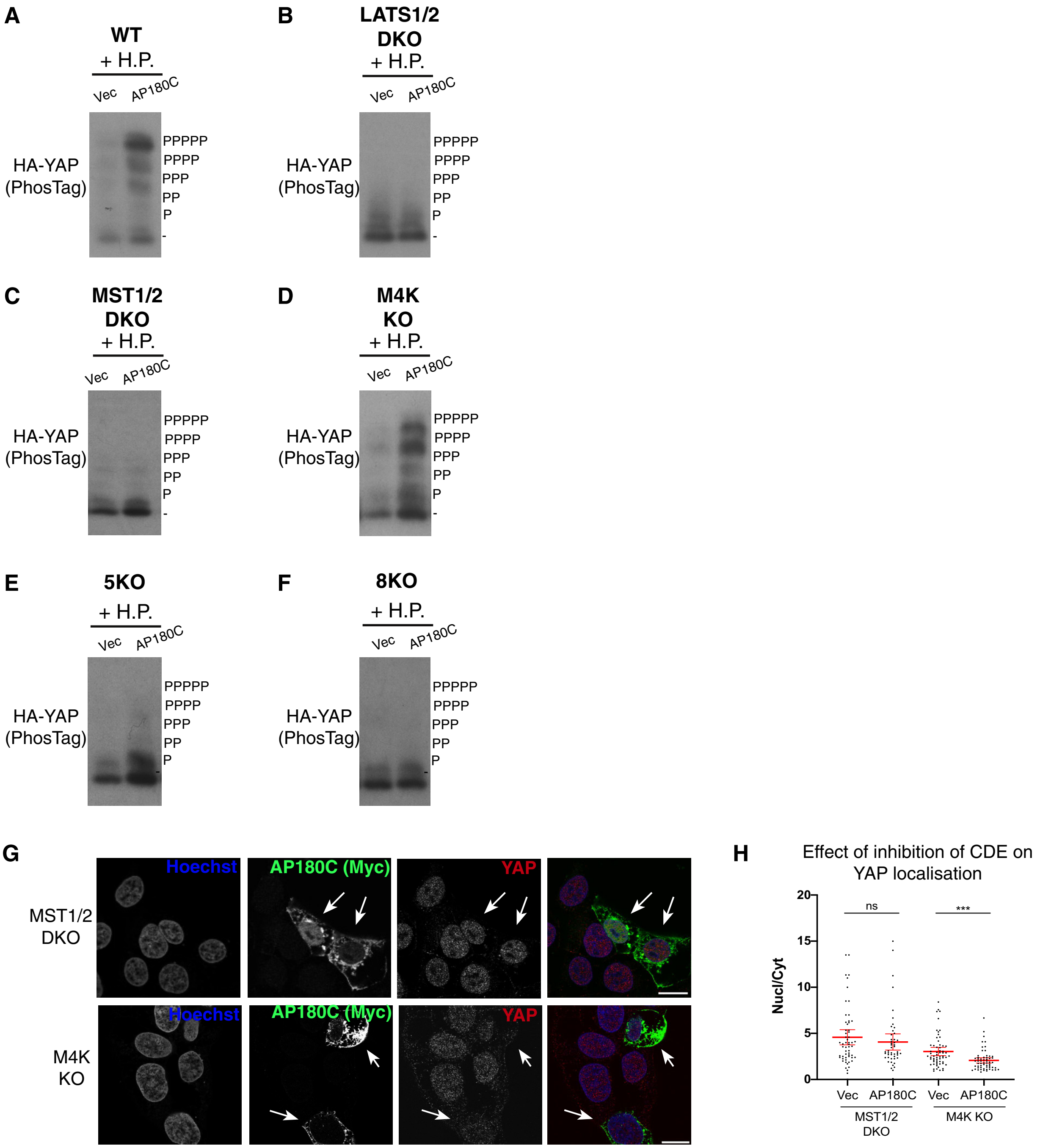
A
WT
+ H.P.
Vec
AP180C
HA-YAP
(PhosTag)
PPPPP
PPPP
PPP
PP
P
-
B
LATS1/2
DKO
+ H.P.
C
MST1/2
DKO
+ H.P.
D
M4K
KO
+ H.P.
E
5KO
+ H.P.
F
8KO
+ H.P.
G
Hoechst
AP180C (Myc)
YAP
MST1/2
DKO
M4K
KO
H
Effect of inhibition of CDE on
YAP localisation
Nucl/Cyt
ns
***
Vec
AP180C
MST1/2
DKO
M4K KO

Figure EV5.
