# Supplementary figures and images for: The Hippo pathway drives the cellular response to hydrostatic pressure
Source: EMBO J. 2022 Jun 15;41(13):e108719. doi: 10.15252/embj.2021108719 (PMC9251841; doi:10.15252/embj.2021108719)

EV2D

PhosTag (YAP)

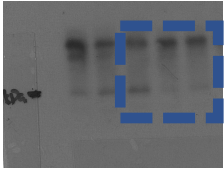

Supplement: Supplementary file 2 — Source Data for Expanded View [file EMBJ-41-e108719-s005.zip › Western_Blots_for_Park_et_al_EV2.pdf]

## EV3A

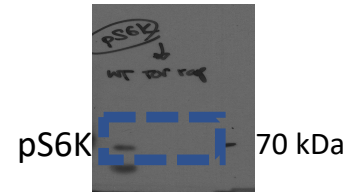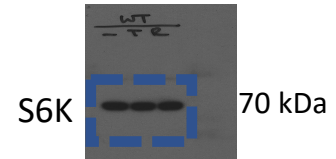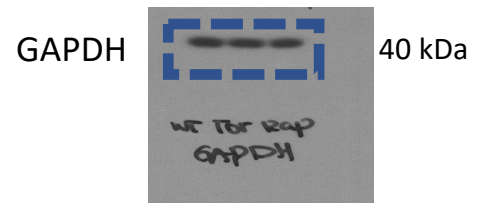

## EV3D

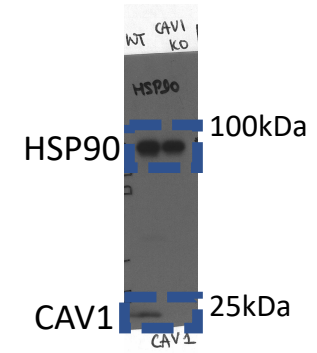

## EV3G

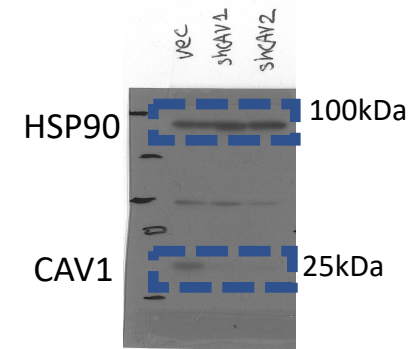

Supplement: Supplementary file 2 — Source Data for Expanded View [file EMBJ-41-e108719-s005.zip › Western_Blots_for_Park_et_al_EV3.pdf]

EV5A

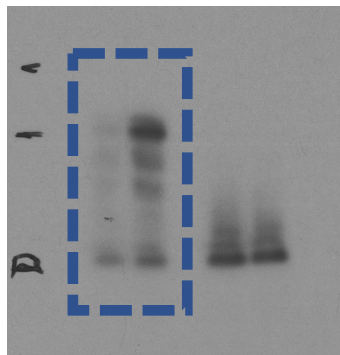

PhosTag (YAP)

EV5B

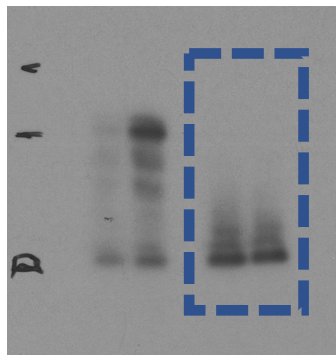

PhosTag (YAP)

EV5C

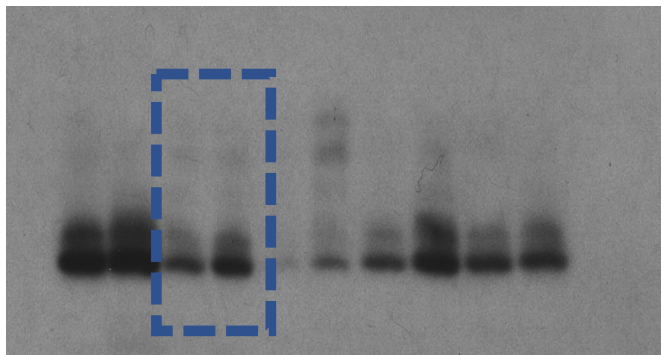

PhosTag (YAP)

EV5D

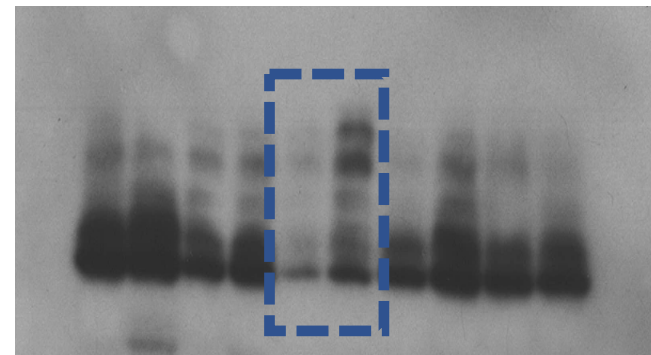

PhosTag (YAP)

EV5E

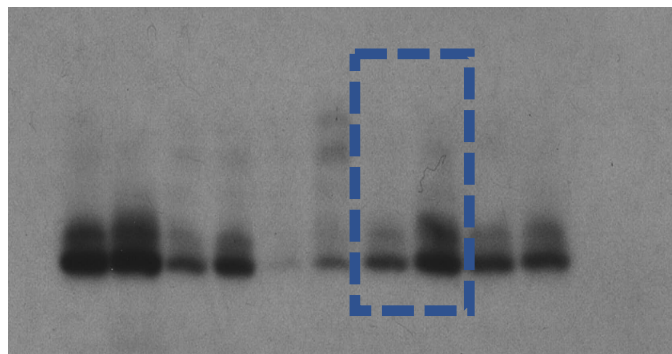

PhosTag (YAP)

EV5F

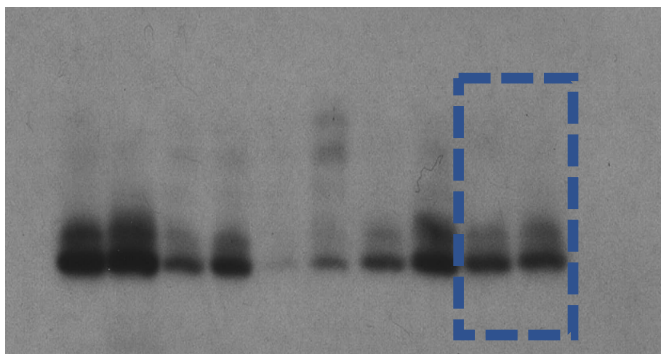

PhosTag (YAP)

Supplement: Supplementary file 2 — Source Data for Expanded View [file EMBJ-41-e108719-s005.zip › Western_Blots_for_Park_et_al_EV5.pdf]

Fig 1A  
PhosTag (YAP)

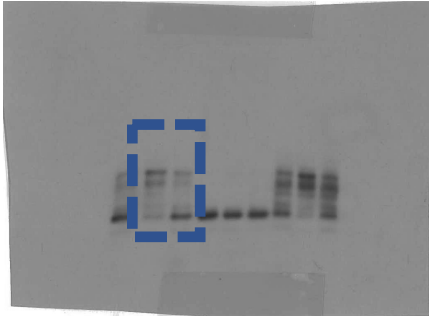

Fig 1B  
PhosTag (TAZ)

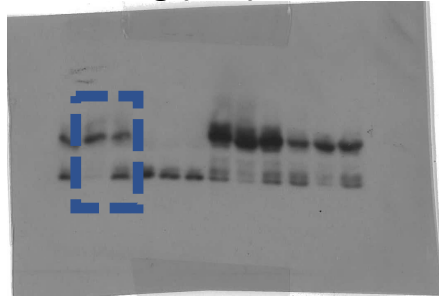

Fig 1H

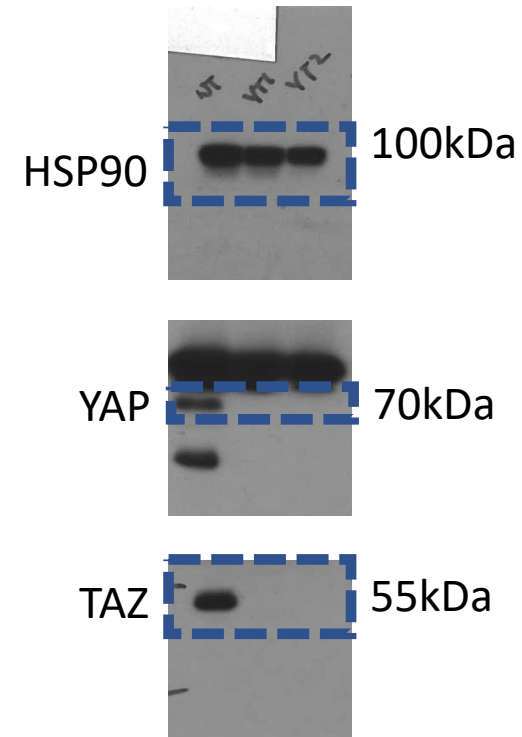

Fig 1D  
PhosTag (YAP) 143B cells

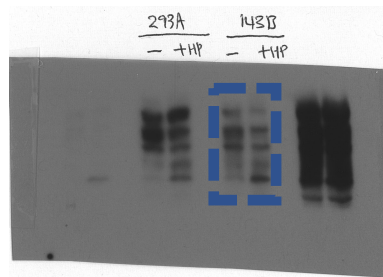

Supplement: Supplementary file 3 — Source Data for Figure 1 [file EMBJ-41-e108719-s001.zip › Western_Blots_for_Park_et_al_Fig1_part1.pdf]

Fig 1C/1E

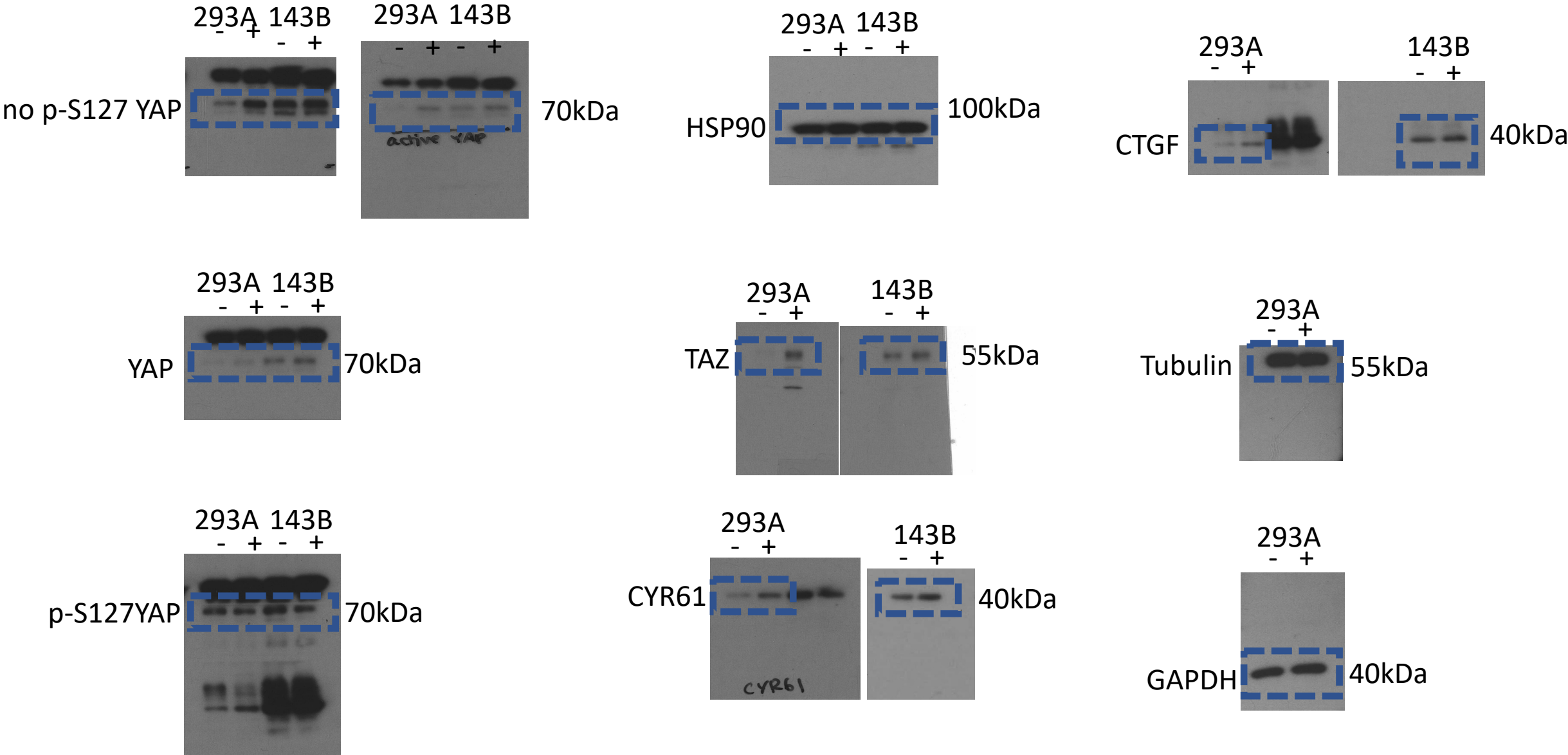

Supplement: Supplementary file 3 — Source Data for Figure 1 [file EMBJ-41-e108719-s001.zip › Western_Blots_for_Park_et_al_Fig1_Part2.pdf]

Fig 2L

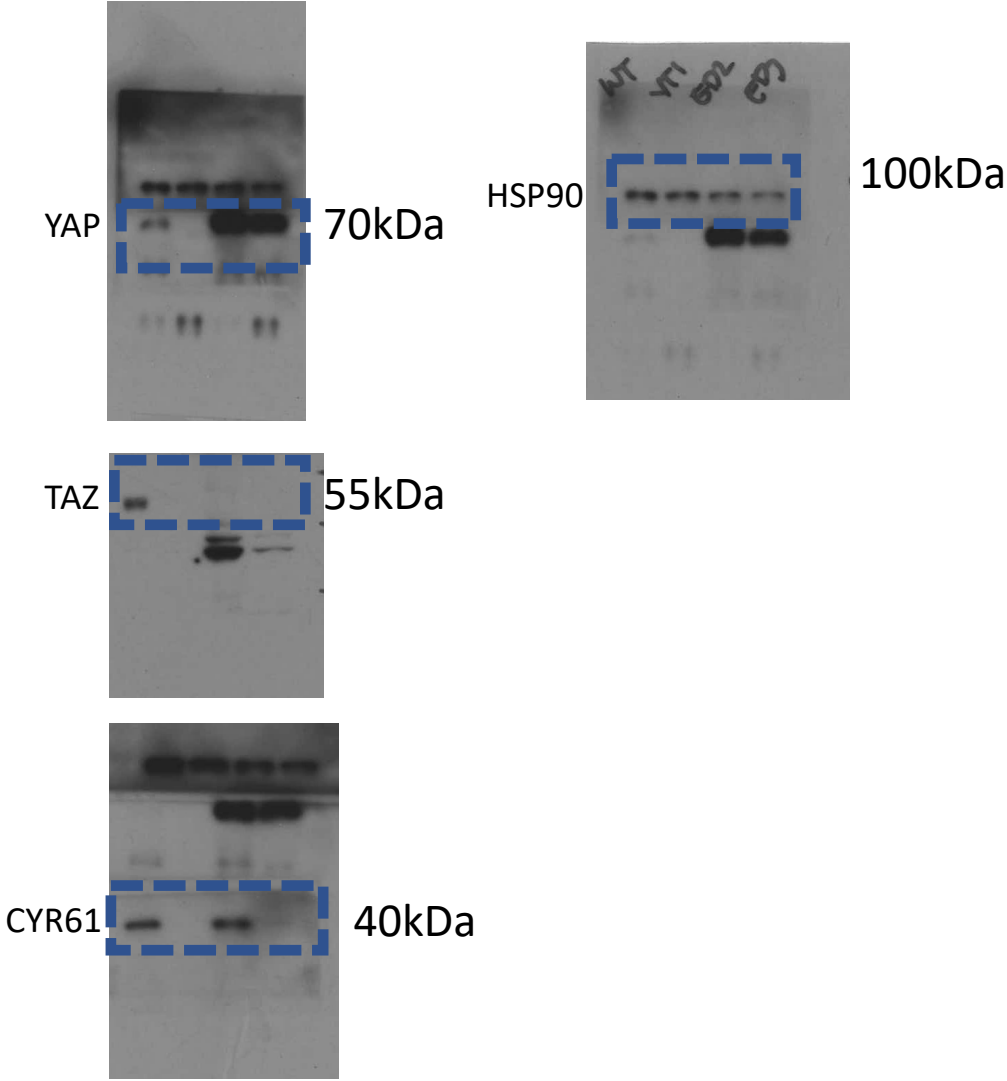

Supplement: Supplementary file 4 — Source Data for Figure 2 [file EMBJ-41-e108719-s004.pdf]
