# Supplementary material for: The Hippo pathway drives the cellular response to hydrostatic pressure
Source: EMBO J. 2022 Jun 15;41(13):e108719. doi: 10.15252/embj.2021108719 (PMC9251841; doi:10.15252/embj.2021108719)
Supplement: Supplementary file 5 [file EMBJ-41-e108719-s002.pdf]

THE EMBO JOURNAL

The Hippo pathway drives the cellular response to hydrostatic pressure

Jiwon Park, Siyang JIA, Donald Salter, Pierre Bagnaninchi, and Carsten Hansen
DOI: 10.15252/embj.2021108719

Corresponding author: Carsten Hansen (Carsten.G.Hansen@ed.ac.uk)

Review Timeline:

Transfer from Review Commons:	13th May 21
Editorial Decision:	21st May 21
Appeal Received:	27th May 21
Editorial Decision:	27th May 21
Revision Received:	20th Dec 21
Editorial Decision:	14th Feb 22
Revision Received:	13th Apr 22
Accepted:	16th May 22

Editor: Ieva Gailite

Transaction Report:

This manuscript was transferred to The EMBO Journal following peer review at Review Commons.

(Note: With the exception of the correction of typographical or spelling errors that could be a source of ambiguity, letters and reports are not edited. Depending on transfer agreements, referee reports obtained elsewhere may or may not be included in this compilation. Referee reports are anonymous unless the Referee chooses to sign their reports.)

Review #1

1. How much time do you estimate the authors will need to complete the suggested revisions:

Estimated time to Complete Revisions (Required)

(Decision Recommendation)

Less than 1 month

2. Evidence, reproducibility and clarity:

Evidence, reproducibility and clarity (Required)

In this interesting manuscript, the authors characterise the role of the Hippo-YAP/TAZ pathway in the cellular response to hydrostatic pressure. When oscillating waves of extracellular hydrostatic pressure are applied to cells in culture, YAP nuclear localisation becomes moderately increased, along with the expression of YAP target genes such as CTGF and CYR61. In the absence of YAP/TAZ, cells cannot increase their volume in response to hydrostatic pressure (normally an ~8% increase, which is reduced to 5% in the YAP/TAZ double knockout cells). While the magnitude of this effect is mild, the statistical analysis and time course data indicate that the effect is robust and reproducible. The authors then implicate the actin cytoskeleton as crucial for sensing hydrostatic pressure, while microtubules appear to play no major role in this response. Interestingly, they also show that hydrostatic pressure increases endocytic update, which they show also depends on the level of activity of YAP/TAZ, suggesting that YAP/TAZ activation must induce an increased rate of endocytosis in these cells.

3. Significance:

Significance (Required)

The primary significance is that the phenomenon of how cells respond to pressure is poorly understood, and these results suggest a possible molecular mechanism that may mediate part of this response. Overall, these are interesting and important findings and I recommend publication without delay.

Review #2

1. How much time do you estimate the authors will need to complete the suggested revisions:

Estimated time to Complete Revisions (Required)

(Decision Recommendation)

Between 1 and 3 months

2. Evidence, reproducibility and clarity:

Evidence, reproducibility and clarity (Required)

In the present manuscript, Park et al investigate the role of the Hippo pathway and its transcriptional co-activators YAP and TAZ in the response to elevated hydrostatic pressure. They combine engineered cell lines, digital holographic imaging to measure cell volumes and a microfluidics setup to manipulate hydrostatic pressure (HP). The authors show that oscillating HP induces YAP nuclear localization and cyclic volume changes that are YAP-dependent. Mechanistically, they implicate clathrin-dependent endocytosis as both a potential upstream signal that relay HP to YAP and an effector of YAP in HP-induced volume changes.

The data are extensive and carefully quantified. The effect of HP on the Hippo pathway has not been investigated to date, therefore the topic should be of broad interest to cell and cancer biologists. However, there are some important issues for the authors to address before this work can be published.

Major points:

1. Figure 1J: there doesn't appear to be a control gene against which the increases in Cyr61 and CTGF mRNA levels have been normalized. The authors should perform the experiments again with such a control (or ideally more than one) to ensure the treatment does not induce a general increase in transcription.
2. The timescale of the volume change response to cyclic HP implies it is an almost instantaneous response and recovery on the part of the cells. This is not compatible with a transcriptional response, and even seems fast for a change in endocytosis, so presumably there are water/ion fluxes involved. In addition, due to the cyclic nature of the stimulus, it is hard to discern whether the effect of increased HP on cell volume is an increase or a decrease. Could the authors therefore do this experiment over a 2h+ time period as in Figure 1? In this setup they could test if wt cells gradually increase their average volume due to YAP activation (and if this fails to occur in the Y/T KO cells).

In addition, can the authors exclude the possibility that the changes in cell volume observed are due to the deformation of the chamber in response to vacuum application? Is it for example possible to measure the volume of glass beads in the experimental setup, as these should not change volume upon cyclic changes in HP.

3. In order to strengthen the connection between endocytosis and YAP activation, the authors should test whether AP180C expression suppresses the increase in YAP nuclear levels upon HP treatment.
4. The connection between HP-induced volume change/endocytosis and the cytoskeletal manipulations (particularly tubulin) is not straightforward. Although the cytoskeletal drug

treatments show some potentially interesting changes, it is not clear how these can be interpreted. For instance, the effect of F-actin depletion in reversing the volume change elicited by LATS KO plus might be very pleiotropic given the two drug treatments. Given this, the manuscript would be less dense and more focused without Figures 3 and 4.
5. Even if the response to HP is dampened upon YAP/TAZ silencing and endocytosis blockage, the cells still do respond. Therefore, the authors should make it clear in their discussion that other independent mechanism are likely to be at play that participate in the HP response.

Minor points:

1. In general, the authors should ensure the figures are presented in the text and in the figures in a consistent order. For example, in figure 2 panels A-D are not presented in the same order.
2. "First, we analysed WT cells treated with CytD or LatB under cyclic fluid pressure, which revealed that the cellular response to hydrostatic pressure in WT cells is strongly dependent on the actin cytoskeleton (Fig 3F, H)."
There is no Fig 3H.
3. "This highlights that LATS1/2 deficient cells have additional mechanisms to ensure cellular response to hydrostatic pressure (Fig 3F-I and Sup Fig 2A-D)."
There is no Fig 3H, I.
4. Figure 5G: if the effect of high pressure on volume change is YAP/TAZ-dependent, how do the authors explain that the response is abolished in LATS1/2 ko cells.
5. Why is YAP localization measured as Nuc/Cyt ratios in Figure 1 and Cyt/Nuc ratios in Figure 5?

3. Significance:

Significance (Required)

See above.

Dear *EMBO Journal*

It is our pleasure to submit our manuscript "The Hippo pathway drives the cellular response to hydrostatic pressure" to *EMBO Journal.* The manuscript has been fully reviewed by Review commons. Both reviewers complement both the rigor of our studies and the impact of our findings. Please see below the reviewers and our comments. Our response in blue.

We are confident that our manuscript will appeal to *EMBO Journal's* readership. We hope you enjoy reading it, and hope, that you, after we have carried out our suggested revision experiments find the paper to be suitable for publication in *EMBO Journal*.

We also attach our updated manuscript, which contains the requested changes highlighted by reviewer 2 under "minor" comments. These are mainly regarding figure references.

We are looking forward to hearing from you,

Kind regards,

Carsten Gram Hansen

Reviewer #1 (Evidence, reproducibility and clarity (Required)):

In this interesting manuscript, the authors characterise the role of the Hippo-YAP/TAZ pathway in the cellular response to hydrostatic pressure. When oscillating waves of extracellular hydrostatic pressure are applied to cells in culture, YAP nuclear localisation becomes moderately increased, along with the expression of YAP target genes such as CTGF and CYR61. In the absence of YAP/TAZ, cells cannot increase their volume in response to hydrostatic pressure (normally an ~8% increase, which is reduced to 5% in the YAP/TAZ double knockout cells). While the magnitude of this effect is mild, the statistical analysis and time course data indicate that the effect is robust and reproducible. The authors then implicate the actin cytoskeleton as crucial for sensing hydrostatic pressure, while microtubules appear to play no major role in this response. Interestingly, they also show that hydrostatic pressure increases endocytic update, which they show also depends on the level of activity of YAP/TAZ, suggesting that YAP/TAZ activation must induce an increased rate of endocytosis in these cells.

Reviewer #1 (Significance (Required)):

The primary significance is that the phenomenon of how cells respond to pressure is poorly understood, and these results suggest a possible molecular mechanism that may mediate part of this response. Overall, these are interesting and important findings and I recommend publication without delay.

Response: We thank the reviewer for his/her time, and supportive statement. We are pleased that reviewer 1 states that our findings are "**interesting and important**" and "**recommend publication without delay**".

Reviewer #2 (Evidence, reproducibility and clarity (Required)):

In the present manuscript, Park et al investigate the role of the Hippo pathway and its transcriptional co-activators YAP and TAZ in the response to elevated hydrostatic pressure. They combine engineered cell lines, digital holographic imaging to measure cell volumes and a microfluidics setup to manipulate hydrostatic pressure (HP). The authors show that oscillating HP induces YAP nuclear localization and cyclic volume changes that are YAP-dependent. Mechanistically, they implicate clathrin-dependent endocytosis as both a potential upstream signal that relay HP to YAP and an effector of YAP in HP-induced volume changes.

The data are extensive and carefully quantified. The effect of HP on the Hippo pathway has not been investigated to date, therefore the topic should be of broad interest to cell and cancer biologists. However, there are some important issues for the authors to address before this work can be published.

We thank the reviewer for the supportive comments of our manuscript and are pleased that he/she highlights that our data is "**extensive and carefully quantified**" and that the topic is of "**broad interest to cell and cancer biologists**".

Major points:

1. Figure 1J: there doesn't appear to be a control gene against which the increases in Cyr61 and CTGF mRNA levels have been normalized. The authors should perform the experiments again with such a control (or ideally more than one) to ensure the treatment does not induce a general increase in transcription.

The genes have been normalised to *HPRT1* gene expression levels. This is a widely used normalisation gene, we apologies that we didn't make this clearer in the manuscript and will include a statement of this in the figure legend.

In addition, we have previously shown, carrying out in silico bioinformatics analysis across 967 cell lines, that *HPRT1* expression does not correlate with YAP/TAZ target genes (Rausch et al, *Cur Bio* 2019).

2. The timescale of the volume change response to cyclic HP implies it is an almost instantaneous response and recovery on the part of the cells. This is not compatible with a transcriptional response, and even seems fast for a change in endocytosis, so presumably there are water/ion fluxes involved. In addition, due to the cyclic nature of the stimulus, it is hard to discern whether the effect of increased HP on cell volume is an increase or a decrease. Could the authors therefore do this experiment over a 2h+ time period as in Figure 1? In this setup they could test if wt cells gradually increase their average volume due to YAP activation (and if this fails to occur in the Y/T KO cells).

We thank the reviewer for this suggestion, and we will analyse this aspect and carry out the suggested experiment.

In addition, can the authors exclude the possibility that the changes in cell volume observed are due to the deformation of the chamber in response to vacuum application? Is it for example possible to measure the volume of glass beads in the experimental setup, as these should not change volume upon cyclic changes in HP.

We appreciate reviewer 2's comment. We are ascertained that deformation of the chamber is not a confounding factor, as different genotypes and cell types behave markedly different in our measurements.
However, we will further investigate this aspect by imaging glass beads, and if this proves technical challenging we will use quartz containers to address this aspect.

3. In order to strengthen the connection between endocytosis and YAP activation, the authors should test whether AP180C expression suppresses the increase in YAP nuclear levels upon HP treatment.
We appreciate reviewer 2's comments and we will carry out AP180C transfection in cells undergoing HP and image these by confocal microscopy.

4. The connection between HP-induced volume change/endocytosis and the cytoskeletal

manipulations (particularly tubulin) is not straightforward. Although the cytoskeletal drug treatments show some potentially interesting changes, it is not clear how these can be interpreted. For instance, the effect of F-actin depletion in reversing the volume change elicited by LATS KO plus might be very pleiotropic given the two drug treatments. Given this, the manuscript would be less dense and more focused without Figures 3 and 4.

We appreciate the comments and suggestions from the reviewer. We feel this link is important to include, as it highlights the intricate relationship between the Hippo pathway and the cellular response to hydrostatic pressure. We will carry out additional experiments utilizing compounds targeting the actin cytoskeleton, including the C-3 transferase, which inhibits Rho, Jasplakonide, an actin filament polymerizing and stabilizing drug, as well as the myosin inhibitor blebbistatin. These experiments will provide additional mechanistic insights into the link between the hippo pathway and hydrostatic pressure.

5. Even if the response to HP is dampened upon YAP/TAZ silencing and endocytosis blockage, the cells still do respond. Therefore, the authors should make it clear in their discussion that other independent mechanism are likely to be at play that participate in the HP response.

We do mention this aspect in the current manuscript, but we will broaden this discussion further. The cellular response mechanism to hydrostatic pressure we have defined and described in our manuscript, we argue play a major role, but we acknowledge that additional factors are implicated. We will include more detailed discussion of this as suggested.

Minor points:

1. In general, the authors should ensure the figures are presented in the text and in the figures in a consistent order. For example, in figure 2 panels A-D are not presented in the same order.
Thanks for the comment, we have now reordered these and updated the order of these figures.

2. "First, we analysed WT cells treated with CytD or LatB under cyclic fluid pressure, which revealed that the cellular response to hydrostatic pressure in WT cells is strongly dependent on the actin cytoskeleton (Fig 3F, H)."
There is no Fig 3H.
Thanks, good point. We apologise for this oversight. We have now deleted this.

3. "This highlights that LATS1/2 deficient cells have additional mechanisms to ensure cellular

response to hydrostatic pressure (Fig 3F-I and Sup Fig 2A-D)."
There is no Fig 3H, I.
Thanks, good point. We apologise for this oversight. We have now deleted this.

4. Figure 5G: if the effect of high pressure on volume change is YAP/TAZ-dependent, how do the authors explain that the response is abolished in LATS1/2 ko cells.
YAP and TAZ are constitutive active in LATS1/2 KO cells. The realisation that LATS1/2 KO cells don't respond as wild type cells is therefore consistent with that this response is YAP/TAZ mediated.

5. Why is YAP localization measured as Nuc/Cyt ratios in Figure 1 and Cyt/Nuc ratios in Figure 5?
This is a good point, and has now been updated so we display this in the same manner.

Reviewer #2 (Significance (Required)):

See above.

We appreciate both reviewers' constructive critique, time, insights, comments and suggestions.

Thank you for submitting your Review Commons manuscript "The Hippo pathway drives the cellular response to hydrostatic pressure" for consideration by The EMBO Journal. I sincerely apologise for the delay in the assessment of your manuscript due to the high number of submissions we receive at the moment. I have now read your study, the reviewer reports and your response letter and discussed the work with my colleagues. I am sorry to say that we have decided not to pursue publication of the manuscript at The EMBO Journal.

We appreciate that your study describes the role of extracellular hydrostatic pressure in regulation of cell size via activation of YAP/TAZ. Further findings show that the effect of hydrostatic pressure is mediated by clathrin-mediated endocytosis, actin cytoskeleton and LATS1/2 activity. While we certainly appreciate that your study presents a novel link between hydrostatic pressure and YAP/TAZ activation, we also noted that the physiological context and relevance of the proposed mechanism of cell volume regulation remain currently unclear. Furthermore, reviewer #2 indicates that the relevance of YAP/TAZ transcriptional activity in for the rapidly induced cell response to hydrostatic pressure changes would have to be clarified. We also find that the molecular links between clathrin endocytosis and YAP/TAZ nuclear localisation and the involvement of LATS1/2 in this process need further investigation. Therefore, I am afraid we concluded that your manuscript in its current form is not a sufficiently strong candidate for publication in The EMBO Journal.

Thank you nevertheless for giving us the opportunity to consider this manuscript. I am sorry that I could not offer more positive news at this time, and I sincerely hope for rapid publication of your manuscript at another Review Commons partner journal.

____

==========================

Rev_Com_number: RC-2021-00708
New_manu_number: EMBOJ-2021-108719
Corr_author: Hansen
Title: The Hippo pathway drives the cellular response to hydrostatic pressure

THE UNIVERSITY of EDINBURGH

Edinburgh, May 26th 2021

This letter is an updated revision plan for our manuscript "*The Hippo pathway drives the cellular response to hydrostatic pressure*" which will inform our discussion tomorrow.

I have attached our original revision plan in the end of this letter. We are carrying out these experiments as planned to address reviewer 2's feedback.

I here highlight our comments and intended experiments to address the three additional points that you raised.

1) The physiological context and relevance of the proposed mechanism of cell volume regulation remain currently unclear.

Cell volume is a fundamental cellular feature. Its regulation is therefore of central interest and regulates multiple fundamental processes, one of which we highlight here is endocytosis. We touched briefly in the manuscript on the physiological significance of elevated oscillating pressure in human diseases and we will be happy to cover in more details these aspects in the updated manuscript to underline a potential link between abnormal interstitial pressure and Yap/Taz activation.
We can provide measurements of additional physical parameters, such as the ratio of dry mass per volume, as well as include further discussions to contextualise these important findings further.

2) YAP/TAZ transcriptional activity in the rapidly induced cell response to hydrostatic pressure changes would have to be clarified.

   A) We infer that the abrupt YAP/TAZ activation is a cellular feedback mechanism as highlighted in the manuscript. We will aim to provide more molecular details on the sensing and mechanotransduction. We will do this by investigating additional YAP/TAZ transcriptional targets as mediators of this cellular function.
   B) We will seek to use the "flipper" membrane tension probe to determine if cell membrane tension is different in our cell lines. This will likely add additional mechanistic insights.

3) The molecular links between clathrin endocytosis and YAP/TAZ nuclear localisation and the involvement of LATS1/2 in this process need further investigation.

We will take advantage of our library of knock out (KO) cells, and include additional KO lines not present in the current version of the manuscript. LATS1/2 are activated by direct phosphorylation by the Hippo kinases MST1/2, but also in parallel by the MAP4K1/2/3/4/6/7 kinases (Ref 1). We will compare LAST1/2 KO cells, as well as of MST1/2 KO, MM-5KO (devoid of MAP4K4/6/7 and MST1/2 KO), and MM-8KO (combined

deletion of MST1/2 and MAP4K1/2/3/4/6/7. These cell lines will be compared to WT cells and provide us with unique opportunities to gain mechanistic insights. This will allow us to directly establish if the clathrin dependent endocytosis effect is LATS1/2 mediated (which we have exploratory data confirming that it is), but also which "arm" of the upstream LATS1/2 activating kinases that are engaged and sensitive to hydrostatic pressure and CDE. Experimentally, we will co-transfect these cell lines with the dominant negative CDE construct (AP180C) with low levels of tagged YAP. These assays will be examined by PhosTag (which allow us to determine phosphorylation status of YAP). We will also in parallel studies examine this effect by IF (nuclear/cytoplasmic YAP localisation). This will provide mechanistic insights, and importantly establish if the effect observed is LATS1/2 and phosphorylation dependent, as well as obtain insights into what part of the upstream kinase cascade is activated and thereby how different upstream regulators feed into the core Hippo pathway.

We will in complimentary experiments probe cell lysates lysates from the KO library for the phosphorylation stage (and thereby infer activity stage) of critical Hippo pathway components.

Reviewer #1 (Evidence, reproducibility and clarity (Required)):

In this interesting manuscript, the authors characterise the role of the Hippo-YAP/TAZ pathway in the cellular response to hydrostatic pressure. When oscillating waves of extracellular hydrostatic pressure are applied to cells in culture, YAP nuclear localisation becomes moderately increased, along with the expression of YAP target genes such as CTGF and CYR61. In the absence of YAP/TAZ, cells cannot increase their volume in response to hydrostatic pressure (normally an ~8% increase, which is reduced to 5% in the YAP/TAZ double knockout cells). While the magnitude of this effect is mild, the statistical analysis and time course data indicate that the effect is robust and reproducible. The authors then implicate the actin cytoskeleton as crucial for sensing hydrostatic pressure, while microtubules appear to play no major role in this response. Interestingly, they also show that hydrostatic pressure increases endocytic update, which they show also depends on the level of activity of YAP/TAZ, suggesting that YAP/TAZ activation must induce an increased rate of endocytosis in these cells.

Reviewer #1 (Significance (Required)):

The primary significance is that the phenomenon of how cells respond to pressure is poorly understood, and these results suggest a possible molecular mechanism that may mediate part of this response. Overall, these are interesting and important findings and I recommend publication without delay.

Response: We thank the reviewer for his/her time, and supportive statement. We are pleased that reviewer 1 states that our findings are "**interesting and important**" and "**recommend publication without delay**".

Reviewer #2 (Evidence, reproducibility and clarity (Required)):

In the present manuscript, Park et al investigate the role of the Hippo pathway and its transcriptional co-activators YAP and TAZ in the response to elevated hydrostatic pressure. They combine engineered cell lines, digital holographic imaging to measure cell volumes and a microfluidics setup to manipulate hydrostatic pressure (HP). The authors show that oscillating HP induces YAP nuclear localization and cyclic volume changes that are YAP-dependent. Mechanistically, they implicate clathrin-dependent endocytosis as both a potential upstream signal that relay HP to YAP and an effector of YAP in HP-induced volume changes.

The data are extensive and carefully quantified. The effect of HP on the Hippo pathway has not been investigated to date, therefore the topic should be of broad interest to cell and cancer biologists. However, there are some important issues for the authors to address before this work can be published.

We thank the reviewer for the supportive comments of our manuscript and are pleased that he/she highlights that our data is "**extensive and carefully quantified**" and that the topic is of "**broad interest to cell and cancer biologists**".

Major points:

1. Figure 1J: there doesn't appear to be a control gene against which the increases in Cyr61 and CTGF mRNA levels have been normalized. The authors should perform the experiments again with such a control (or ideally more than one) to ensure the treatment does not induce a general increase in transcription.

The genes have been normalised to *HPRT1* gene expression levels. This is a widely used normalisation gene, we apologies that we didn't make this clearer in the manuscript and will include a statement of this in the figure legend.

In addition, we have previously shown, carrying out in silico bioinformatics analysis across 967 cell lines, that *HPRT1* expression does not correlate with YAP/TAZ target genes (Rausch et al, *Cur Bio* 2019).

2. The timescale of the volume change response to cyclic HP implies it is an almost instantaneous response and recovery on the part of the cells. This is not compatible with a transcriptional response, and even seems fast for a change in endocytosis, so presumably there are water/ion fluxes involved. In addition, due to the cyclic nature of the stimulus, it is hard to discern whether the effect of increased HP on cell volume is an increase or a decrease. Could the authors therefore do this experiment over a 2h+ time period as in Figure 1? In this setup they could test if wt cells gradually increase their average volume due to YAP activation (and if this fails to occur in the Y/T KO cells).

We thank the reviewer for this suggestion, and we will analyse this aspect and carry out the suggested experiment.

In addition, can the authors exclude the possibility that the changes in cell volume observed are due to the deformation of the chamber in response to vacuum application? Is it for example possible to measure the volume of glass beads in the experimental setup, as these should not change volume upon cyclic changes in HP.

We appreciate reviewer 2's comment. We are ascertained that deformation of the chamber is not a confounding factor, as different genotypes and cell types behave markedly different in our measurements.
However, we will further investigate this aspect by imaging glass beads, and if this proves technical challenging we will use quartz containers to address this aspect.

3. In order to strengthen the connection between endocytosis and YAP activation, the authors should test whether AP180C expression suppresses the increase in YAP nuclear levels upon HP treatment.
We appreciate reviewer 2's comments and we will carry out AP180C transfection in cells undergoing HP and image these by confocal microscopy.

4. The connection between HP-induced volume change/endocytosis and the cytoskeletal

manipulations (particularly tubulin) is not straightforward. Although the cytoskeletal drug treatments show some potentially interesting changes, it is not clear how these can be interpreted. For instance, the effect of F-actin depletion in reversing the volume change elicited by LATS KO plus might be very pleiotropic given the two drug treatments. Given this, the manuscript would be less dense and more focused without Figures 3 and 4.

We appreciate the comments and suggestions from the reviewer. We feel this link is important to include, as it highlights the intricate relationship between the Hippo pathway and the cellular response to hydrostatic pressure. We will carry out additional experiments utilizing compounds targeting the actin cytoskeleton, including the C-3 transferase, which inhibits Rho, Jasplakonide, an actin filament polymerizing and stabilizing drug, as well as the myosin inhibitor blebbistatin. These experiments will provide additional mechanistic insights into the link between the hippo pathway and hydrostatic pressure.

5. Even if the response to HP is dampened upon YAP/TAZ silencing and endocytosis blockage, the cells still do respond. Therefore, the authors should make it clear in their discussion that other independent mechanism are likely to be at play that participate in the HP response.

We do mention this aspect in the current manuscript, but we will broaden this discussion further. The cellular response mechanism to hydrostatic pressure we have defined and described in our manuscript, we argue play a major role, but we acknowledge that additional factors are implicated. We will include more detailed discussion of this as suggested.

Minor points:

1. In general, the authors should ensure the figures are presented in the text and in the figures in a consistent order. For example, in figure 2 panels A-D are not presented in the same order.
Thanks for the comment, we have now reordered these and updated the order of these figures.

2. "First, we analysed WT cells treated with CytD or LatB under cyclic fluid pressure, which revealed that the cellular response to hydrostatic pressure in WT cells is strongly dependent on the actin cytoskeleton (Fig 3F, H)."
There is no Fig 3H.
Thanks, good point. We apologise for this oversight. We have now deleted this.

3. "This highlights that LATS1/2 deficient cells have additional mechanisms to ensure cellular

response to hydrostatic pressure (Fig 3F-I and Sup Fig 2A-D)."
There is no Fig 3H, I.
Thanks, good point. We apologise for this oversight. We have now deleted this.

4. Figure 5G: if the effect of high pressure on volume change is YAP/TAZ-dependent, how do the authors explain that the response is abolished in LATS1/2 ko cells.
YAP and TAZ are constitutive active in LATS1/2 KO cells. The realisation that LATS1/2 KO cells don't respond as wild type cells is therefore consistent with that this response is YAP/TAZ mediated.

5. Why is YAP localization measured as Nuc/Cyt ratios in Figure 1 and Cyt/Nuc ratios in Figure 5?
This is a good point, and has now been updated so we display this in the same manner.

Reviewer #2 (Significance (Required)):

See above.

We appreciate both reviewers' constructive critique, time, insights, comments and suggestions.

Thank you for contacting me regarding the recent decision on your manuscript. Based on your preliminary revision plan and our discussion, I would like to invite you to submit a revised manuscript in which you address the comments of reviewer #2 and expand the analysis of the molecular links between clathrin endocytosis and YAP/TAZ activation, as well as provide further insights into the nature of the rapid cell response to hydrostatic pressure vs the proposed feedback loop via YAP/TAZ activation. Please also significantly expand the introduction and discussion on the relevance of hydrostatic pressure in physiological and disease contexts.

I should add that it is The EMBO Journal policy to allow only a single major round of revision and that it is therefore important to resolve the main concerns at this stage. We have extended our 'scooping protection policy' beyond the usual 3-month revision timeline to cover the period required for a full revision to address the essential experimental issues. This means that competing manuscripts published during revision period will not negatively impact on our assessment of the conceptual advance presented by your study. Please contact me if you see a paper with related content published elsewhere to discuss the appropriate course of action.

When preparing your letter of response to the referees' comments, please bear in mind that this will form part of the Review Process File and will therefore be available online to the community. For more details on our Transparent Editorial Process, please visit our website: https://www.embopress.org/page/journal/14602075/authorguide#transparentprocess

Please feel free to contact me if have any further questions regarding the revision. Thank you for the opportunity to consider your work for publication. I look forward to your revision.

THE UNIVERSITY of EDINBURGH

Reviewer #1 (Evidence, reproducibility and clarity (Required)):

In this interesting manuscript, the authors characterise the role of the Hippo-YAP/TAZ pathway in the cellular response to hydrostatic pressure. When oscillating waves of extracellular hydrostatic pressure are applied to cells in culture, YAP nuclear localisation becomes moderately increased, along with the expression of YAP target genes such as CTGF and CYR61. In the absence of YAP/TAZ, cells cannot increase their volume in response to hydrostatic pressure (normally an ~8% increase, which is reduced to 5% in the YAP/TAZ double knockout cells). While the magnitude of this effect is mild, the statistical analysis and time course data indicate that the effect is robust and reproducible. The authors then implicate the actin cytoskeleton as crucial for sensing hydrostatic pressure, while microtubules appear to play no major role in this response. Interestingly, they also show that hydrostatic pressure increases endocytic update, which they show also depends on the level of activity of YAP/TAZ, suggesting that YAP/TAZ activation must induce an increased rate of endocytosis in these cells.

Reviewer #1 (Significance (Required)):

The primary significance is that the phenomenon of how cells respond to pressure is poorly understood, and these results suggest a possible molecular mechanism that may mediate part of this response. Overall, these are interesting and important findings and I recommend publication without delay.

Response: We thank the reviewer for his/her time, and supportive statement. We are pleased that reviewer 1 states that our findings are "**interesting and important**" and "**recommend publication without delay**".

Reviewer #2 (Evidence, reproducibility and clarity (Required)):

In the present manuscript, Park et al investigate the role of the Hippo pathway and its transcriptional co-activators YAP and TAZ in the response to elevated hydrostatic pressure. They combine engineered cell lines, digital holographic imaging to measure cell volumes and a microfluidics setup to manipulate hydrostatic pressure (HP). The authors show that oscillating HP induces YAP nuclear localization and cyclic volume changes that are YAP-dependent. Mechanistically, they implicate clathrin-dependent endocytosis as both a potential upstream signal that relay HP to YAP and an effector of YAP in HP-induced volume

changes.

The data are extensive and carefully quantified. The effect of HP on the Hippo pathway has not been investigated to date, therefore the topic should be of broad interest to cell and cancer biologists. However, there are some important issues for the authors to address before this work can be published.

We thank the reviewer for the supportive comments of our manuscript and are pleased that he/she highlights that our data is "**extensive and carefully quantified**" and that the topic is of "**broad interest to cell and cancer biologists**".

Major points:

1. Figure 1J: there doesn't appear to be a control gene against which the increases in Cyr61 and CTGF mRNA levels have been normalized. The authors should perform the experiments again with such a control (or ideally more than one) to ensure the treatment does not induce a general increase in transcription.

These figures are now Figure 1 I and J.
The genes have been normalised to *HPRT1* gene expression levels. This is a widely used normalisation gene, we apologies that we didn't make this clearer in the manuscript and have emphasized this further in the material and methods.

In addition, we have previously shown, carrying out in silico bioinformatics analysis across 967 cell lines, that *HPRT1* expression does not correlate with YAP/TAZ target genes (Rausch et al, *Cur Bio* 2019).

2. The timescale of the volume change response to cyclic HP implies it is an almost instantaneous response and recovery on the part of the cells. This is not compatible with a transcriptional response, and even seems fast for a change in endocytosis, so presumably there are water/ion fluxes involved. In addition, due to the cyclic nature of the stimulus, it is hard to discern whether the effect of increased HP on cell volume is an increase or a decrease. Could the authors therefore do this experiment over a 2h+ time period as in Figure 1? In this setup they could test if wt cells gradually increase their average volume due to YAP activation (and if this fails to occur in the Y/T KO cells).

We thank the reviewer for these comments and suggestions. We agree with the reviewer that the fast dynamics are not directly transcriptional driven.

However, the response is YAP/TAZ mediated, as cells without YAP/TAZ change the response. We have analysed the additional aspects raised by the reviewer, and carried out the suggested experiments. The main findings are as follow.

1) We identify a specific range of YAP/TAZ activated hydrostatic pressure induced genes, including water channels, and that have previously been identified to regulate cellular volume (Fig 2 Q,R). We also identify that YAP/TAZ KO cells have a lower plasma membrane tension (Fig 2 I,J). These findings provide mechanistic insights and plausible scenarios of long-term cellular effect.
2) We find that the overall cellular volume after 2 hours oscillating pressure does not change compared to pre pressure. However, the cellular response to hydrostatic pressure does. As such WT cells pre stimulated with oscillating hydrostatic pressure phenocopy YAP/TAZ cells (Fig 2 O,P).

In addition, can the authors exclude the possibility that the changes in cell volume observed are due to the deformation of the chamber in response to vacuum application? Is it for example possible to measure the volume of glass beads in the experimental setup, as these should not change volume upon cyclic changes in HP.

We appreciate reviewer 2's comment. As proposed by the reviewer we investigated this aspect by imaging glass beads but unfortunately the large change of refractive index induced by the glass beads generated phase wrapping (Phi >2pi) that confounded the holographic reconstruction algorithm.
However, we are ascertained that deformation of the chamber is not a confounding factor, as different genotypes and cell types behave markedly different in our measurements, and that genotypes exhibit either decrease or increase in volume change upon different chemical treatment.

3. In order to strengthen the connection between endocytosis and YAP activation, the authors should test whether AP180C expression suppresses the increase in YAP nuclear levels upon HP treatment.
We appreciate reviewer 2's comments and have carried out the experiments suggested, including AP180C transfection in cells undergoing HP and imaged these by confocal microscopy Fig 5 E, F and EV 5 G,H . We have additionally examined these aspects by PhosTag gels using a range of KO cells lines.
AP180C limits, as predicted the hydrostatic pressure-based activity induction of YAP (Fig 5 E, F and EV 5 A-H), in a phosphorylation and MST1/2 dependent manner (EV 5A-H).

4. The connection between HP-induced volume change/endocytosis and the cytoskeletal

manipulations (particularly tubulin) is not straightforward. Although the cytoskeletal drug treatments show some potentially interesting changes, it is not clear how these can be interpreted. For instance, the effect of F-actin depletion in reversing the volume change elicited by LATS KO plus might be very pleiotropic given the two drug treatments. Given this, the manuscript would be less dense and more focused without Figures 3 and 4.

We appreciate the comments and suggestions from the reviewer. We feel this link is important to include, and although admittable we don't have a full mechanistic understanding, this data highlights the intricate relationship between the Hippo pathway and the cellular response to hydrostatic pressure. We have carried out additional experiments (Fig 3 and 4). These experiments provide additional insights into the link between the hippo pathway and hydrostatic pressure. As our manuscript is one of the first papers investigating the cellular response to hydrostatic pressure with cell level resolution, we feel these complexities benefit the readership.

5. Even if the response to HP is dampened upon YAP/TAZ silencing and endocytosis blockage, the cells still do respond. Therefore, the authors should make it clear in their discussion that other independent mechanism are likely to be at play that participate in the HP response.

We did mention this aspect briefly in the previous version of the manuscript, but have now broadened this discussion further. The cellular response mechanism to hydrostatic pressure we have defined and described in our manuscript, we argue play a major role, but we acknowledge that additional factors are implicated. We have included a more detailed discussion of this as suggested.

Minor points:

1. In general, the authors should ensure the figures are presented in the text and in the figures in a consistent order. For example, in figure 2 panels A-D are not presented in the same order.
Thanks for the comment, we have now reordered these and updated the order of these and other figures.

2. "First, we analysed WT cells treated with CytD or LatB under cyclic fluid pressure, which revealed that the cellular response to hydrostatic pressure in WT cells is strongly dependent on the actin cytoskeleton (Fig 3F, H)."
There is no Fig 3H.
Thanks, good point. We apologise for this oversight. We have now deleted this.

3. "This highlights that LATS1/2 deficient cells have additional mechanisms to ensure cellular

response to hydrostatic pressure (Fig 3F-I and Sup Fig 2A-D)."
There is no Fig 3H, I.
Thanks, good point. We apologise for this oversight. We have now deleted this.

4. Figure 5G: if the effect of high pressure on volume change is YAP/TAZ-dependent, how do the authors explain that the response is abolished in LATS1/2 ko cells.
YAP and TAZ are constitutive active in LATS1/2 KO cells. The realisation that LATS1/2 KO cells don't respond as wild type cells is therefore consistent with that this response is YAP/TAZ mediated.

5. Why is YAP localization measured as Nuc/Cyt ratios in Figure 1 and Cyt/Nuc ratios in Figure 5?
This is a good point, and has now been updated so we display this in the same manner. We thank the reviewer for pointing out this inconsistency to us.

Reviewer #2 (Significance (Required)):

See above.

We appreciate both reviewers' constructive critique, time, insights, comments and suggestions.

Thank you for the submission of your revised manuscript to The EMBO Journal and please accept my apologies for the delay in responding. I have received the comments from one referee that now considers that all the major concerns regarding your study have been addressed S/he has however a couple of minor issues that will require some further discussion.

In addition, before we can proceed with the acceptance of your study, there are a few minor editorial issues that will also need your attention:

- Figure 2 is provided in two separate files spanning two pages. It must be a single file and single page figure. If it is too big, please consider splitting it into two figures or placing some of the panels in EV or appendix figures.
- You provide an Excel file containing a table with primer sequences. Unfortunately, we cannot properly link this type of files to the paper, so we suggest you include this list as Table 1 in the main manuscript, adding the corresponding callouts in the text (probably in the methods section). The Word document including the list of reagents is fine.
- Please provide 3 to 5 keywords describing your findings.
- The respective author contributions are missing and need to be provided.
- You need to add a "Competing interests and disclosure statement" section to the main text of the manuscript.

You need to provide a synopsis. The paper's synopsis is composed of:
- a short 'blurb' text summarizing in two sentences the study (max. 250 characters) and three to four 'bullet points' highlighting the main findings. Bullet points and standfirst text should be submitted as a separate manuscript file in LaTeX, RTF or MS Word format.
- A "synopsis image", which can be used as a "visual title" for the synopsis section of your paper. While you provide this image, it needs to be resized. This figure must be in PNG or JPG format with pixel dimensions of 550 x 300-600 (width x height).

Please let me know if you have any further questions regarding any of these points. Thank you again for giving us the chance to consider your manuscript for The EMBO Journal and congratulations!

I look forward to receiving the final version of your manuscript with these minor changes included.

Referee #1:

The authors performed several new experiments that have answered my queries and strengthened the manuscript. I am happy to support publication in the EMBO Journal, provided the authors clarify the points below.

1. I found the way the differences between "pre-conditioning" response in cell volume to HP (Figure 2O) and the first batch of experiments on the effect of HP on cell volume (Figure 2G, H) are presented to be confusing. What is the difference between these experiments? To help the reader understand, the authors should state in the figure legends exactly how long the cells were exposed to HP, and when the cell volume was measured. Calling figure 2O a "pre-conditioning" experiment seems misleading to me. As far as I can make out, these cells were exposed to fluctuating HP for two hours and assayed for cell volume straight away, and in 2G, H the same thing was done with a 1 hour exposure, or did I miss something? It would be more appropriate to say that over time the cells adapt to HP and their volume returns to homeostatic levels after two hours. Ideally, the authors should provide the timing of HP application for all experiments in the fiure legends, or at least say the cells were assayed after 1 hour oscillating HP unless otherwise stated.

2. Some aspects of the discussion should be clarified.
- The authors show that, although cell volume increases upon HP (Figure 2H), clathrin-mediated endocytosis also increases (Figure 5A). This is not entirely consistent with the idea that "clathrin dependent endocytosis provide a membrane reservoir" to fuel the size increase upon HP, as in this case endocytosis might be predicted to increase upon HP, leading to more plasma membrane availability to allow for volume increase. The authors should clearly discuss this point and outline their interpretations of these findings.
- Likewise, the discussion of the relationship between HP, clathrin-dependent endocytosis and YAP activation is not very clear. Do the authors believe that there is a clear hierarchy whereby HP increases endocytosis, which then triggers YAP activation by blocking MST1/2 activity, or do they think the situation is more complex?

***

Rev_Com_number: RC-2021-00708
New_manu_number: EMBOJ-2021-108719R1
Corr_author: Hansen
Title: The Hippo pathway drives the cellular response to hydrostatic pressure

THE UNIVERSITY of EDINBURGH

Dear *EMBO J* and reviewer(s)

Thank you very much for your time and constructive feedback during the reviewing stage. Please see below our comments to the minor revisions requested. This letter is split in two, first section considers requests raised by the editor.
Our comments in blue.

Comments raised by the editor

- Figure 2 is provided in two separate files spanning two pages. It must be a single file and single page figure. If it is too big, please consider splitting it into two figures or placing some of the panels in EV or appendix figures.
Thanks for highlighting this. We have moved old Fig 2Q-S, so they are now EV 1K-M to accommodate this. This meant we needed to change the layout a little in the figures (Fig2 and EV1), but otherwise not the order or content.

- You provide an Excel file containing a table with primer sequences. Unfortunately, we cannot properly link this type of files to the paper, so we suggest you include this list as Table 1 in the main manuscript, adding the corresponding callouts in the text (probably in the methods section). The Word document including the list of reagents is fine.
We included the primer sequences both as an excel file and in the manuscript methods section. We have therefore just retained this information in the Word document.

- Please provide 3 to 5 keywords describing your findings.
Thanks, these are
YAP/TAZ, endocytosis, membrane tension, cell volume, holographic imaging

- The respective author contributions are missing and need to be provided.
Thanks, this has now been done using CRediT for author contributions.

- You need to add a "Competing interests and disclosure statement" section to the main text of the manuscript.
Thanks, this has now been included.

You need to provide a synopsis. The paper's synopsis is composed of:
- a short 'blurb' text summarizing in two sentences the study (max. 250 characters) and three to four 'bullet points' highlighting the main findings. Bullet

points and standfirst text should be submitted as a separate manuscript file in LaTeX, RTF or MS Word format.
Thanks, this is now included

- A "synopsis image", which can be used as a "visual title" for the synopsis section of your paper. While you provide this image, it needs to be resized. This figure must be in PNG or JPG format with pixel dimensions of 550 x 300-600 (width x height).
Thanks, we have attached this now.
Please let us know if this is acceptable.

In order to comply with new research funder Open Access publications policies, we have furthermore added a sentence “For the purpose of open access, the author has applied a Creative Commons Attribution (CC BY) licence to any Author Accepted Manuscript version arising from this submission” in the acknowledgement section.

THE UNIVERSITY of EDINBURGH

Dear reviewer(s), thanks for your comments and feedback throughout the process.

1. I found the way the differences between "pre-conditioning" response in cell volume to HP (Figure **2O**) and the first batch of experiments on the effect of HP on cell volume (Figure 2G, H) are presented to be confusing. What is the difference between these experiments? To help the reader understand, the authors should state in the figure legends exactly how long the cells were exposed to HP, and when the cell volume was measured. Calling figure 2O a "pre-conditioning" experiment seems misleading to me. As far as I can make out, these cells were exposed to fluctuating HP for two hours and assayed for cell volume straight away, and in 2G, H the same thing was done with a 1 hour exposure, or did I miss something? It would be more appropriate to say that over time the cells adapt to HP and their volume returns to homeostatic levels after two hours. Ideally, the authors should provide the timing of HP application for all experiments in the fiure legends, or at least say the cells were assayed after 1 hour oscillating HP unless otherwise stated.

Thanks for highlighting this. We apologise that we didn't make this clearer.
In 2G (diagram showing the fluctuations) and in 2H, the images are acquired at the onset of when hydrostatic pressure is turned on, whereafter cells undergo cyclic hydrostatic pressure at 0.1Hz between 0 and 200mBar. We are then acquiring data while the oscillating pressure is applied. Not after one hour. We have now made this clearer in the figure legend by that we in Fig 2H additionally include the statement "Images and data were acquired at the onset of oscillating pressure".
For Fig 2O, we are comparing in the same graph the cell volume of cells at steady state (without experiencing oscillating pressure), with those same cells imaged at steady state to obtain cell volume, but after they have experienced oscillating pressure for 2 hours. These are now labelled "+" and "-" respectively with additional details in the figure legend.
There is therefore a clear experimental difference between Fig 2H and Fig 2O/P. We thank the reviewer for highlighting that this needed clarification and hope that this distinction is now clear.

2. Some aspects of the discussion should be clarified.
- The authors show that, although cell volume increases upon HP (Figure 2H), clathrin-mediated endocytosis also increases (Figure 5A). This is not entirely consistent with the idea that "clathrin dependent endocytosis provide a membrane reservoir" to fuel the size increase upon HP, as in this case endocytosis might be predicted to increase upon HP, leading to more plasma membrane availability to allow for volume increase. The authors should clearly discuss this point and outline their interpretations of these findings.

Thanks for your comment. We interpret this, as overall plasma membrane dynamics, which involves continues cycles of endocytosis, endocytic recycling and exocytosis in order to replenish and redeposit the plasmamembrane. We have now included a statement in the discussion. "*This implies that repeated cycles of rapid internalization of clathrin coated pits, endocytic recycling and exocytosis takes place in order to serve as plasmamembrane buffer in order to replenish the plasma-membrane.*"

- Likewise, the discussion of the relationship between HP, clathrin-dependent endocytosis and YAP activation is not very clear. Do the authors believe that there is a clear hierarchy whereby HP increases endocytosis, which then triggers YAP activation by blocking MST1/2 activity, or do they think the situation is more complex?

This is a good question, and one that we don't feel we have yet fully answered. We are reluctant to make firm statements regarding this, as we currently don't have detailed molecular evidence, and do not want to overstate our findings. We have therefore included a sentence in the discussion

"*How these dynamics are integrated, between changes in hydrostatic pressure, CDE and the Hippo pathway are not yet fully understood. We speculate cellular sensing of membrane tension and overall cellular volume changes may alter protein condensate levels, and overall spatiotemporal localisation of protein complexes, which together might play coordinating roles in this regulation*".

We have in addition corrected a few typos and wordings in the document, and updated the figure referencing according to the change of Figure 2.

We thank the reviewer for his/her time and feedback.

EMBO PRESS

YOU MUST COMPLETE ALL CELLS WITH A PINK BACKGROUND ➔

PLEASE NOTE THAT THIS CHECKLIST WILL BE PUBLISHED ALONGSIDE YOUR PAPER

Corresponding Author Name: Carsten Gram Hansen
Journal Submitted to: EMBO Journal
Manuscript Number: EMBOJ-2021-108719R1

Reporting Checklist For Life Sciences Articles (Rev. June 2017)

This checklist is used to ensure good reporting standards and to improve the reproducibility of published results. These guidelines are consistent with the Principles and Guidelines for Reporting Preclinical Research issued by the NIH in 2014. Please follow the journal's authorship guidelines in preparing your manuscript.

A- Figures
1. Data
The data shown in figures should satisfy the following conditions:

- ➔ the data were obtained and processed according to the field's best practice and are presented to reflect the results of the experiments in an accurate and unbiased manner.
- ➔ figure panels include only data points, measurements or observations that can be compared to each other in a scientifically meaningful way.
- ➔ graphs include clearly labeled error bars for independent experiments and sample sizes. Unless justified, error bars should not be shown for technical replicates.
- ➔ if n< 5, the individual data points from each experiment should be plotted and any statistical test employed should be justified
- ➔ Source Data should be included to report the data underlying graphs. Please follow the guidelines set out in the author ship guidelines on Data Presentation.

2. Captions
Each figure caption should contain the following information, for each panel where they are relevant:

- ➔ a specification of the experimental system investigated (eg cell line, species name).
- ➔ the assay(s) and method(s) used to carry out the reported observations and measurements
- ➔ an explicit mention of the biological and chemical entity(ies) that are being measured.
- ➔ an explicit mention of the biological and chemical entity(ies) that are altered/varied/perturbed in a controlled manner.

- ➔ the exact sample size (n) for each experimental group/condition, given as a number, not a range;
- ➔ a description of the sample collection allowing the reader to understand whether the samples represent technical or biological replicates (including how many animals, litters, cultures, etc.).
- ➔ a statement of how many times the experiment shown was independently replicated in the laboratory.
- ➔ definitions of statistical methods and measures:
  - common tests, such as t-test (please specify whether paired vs. unpaired), simple χ^2 tests, Wilcoxon and Mann-Whitney tests, can be unambiguously identified by name only, but more complex techniques should be described in the methods section;
  - are tests one-sided or two-sided?
  - are there adjustments for multiple comparisons?
  - exact statistical test results, e.g., P values = x but not P values < x;
  - definition of 'center values' as median or average;
  - definition of error bars as s.d. or s.e.m.

Any descriptions too long for the figure legend should be included in the methods section and/or with the source data.

In the pink boxes below, please ensure that the answers to the following questions are reported in the manuscript itself. Every question should be answered. If the question is not relevant to your research, please write NA (non applicable). We encourage you to include a specific subsection in the methods section for statistics, reagents, animal models and human subjects.

B- Statistics and general methods

Please fill out these boxes ➔ (Do not worry if you cannot see all your text once you press return)

1.a. How was the sample size chosen to ensure adequate power to detect a pre-specified effect size?	As these are single cell based experiments we are not readily limited by number of cells we can quantify. No prior sample size or power calculations were carried out. Instead prior experience in picking up meaningful biological changes were implemented.
1.b. For animal studies, include a statement about sample size estimate even if no statistical methods were used.	NA
2. Describe inclusion/exclusion criteria if samples or animals were excluded from the analysis. Were the criteria pre-established?	Samples for IF: Where cells had either multiple nuclei, undergoing mitosis or cell death were omitted for quantification. Samples for DHM, cells that were in contact with other cells were omitted from the nanlysis, as the software can't separate these. Likewise cells undergoing mitosis or cell death also were not included.
3. Were any steps taken to minimize the effects of subjective bias when allocating animals/samples to treatment (e.g. randomization procedure)? If yes, please describe.	For IF, cells were imaged by one scientist and quantified by another investigator.
For animal studies, include a statement about randomization even if no randomization was used.	NA
4.a. Were any steps taken to minimize the effects of subjective bias during group allocation or/and when assessing results (e.g. blinding of the investigator)? If yes please describe.	As highlighted under 3
4.b. For animal studies, include a statement about blinding even if no blinding was done	NA
5. For every figure, are statistical tests justified as appropriate?	Yes. P<0.05 is used as significance value. Exact P values available in the legends.
Do the data meet the assumptions of the tests (e.g., normal distribution)? Describe any methods used to assess it.	None parametric or parametric test were evaluated. None parametric tests were only used if data exhibited a normal distribution. To evaluate data, if not clearly normal destributed when plotted, data were analysed using D'Agostino-Pearson normality test to asist in determining the appropriate statatistical test.
Is there an estimate of variation within each group of data?	No

USEFUL LINKS FOR COMPLETING THIS FORM

http://www.antibodypedia.com
http://1degreebio.org
http://www.equator-network.org/reporting-guidelines/improving-bioscience-research-repor
http://grants.nih.gov/grants/olaw/olaw.htm
http://www.mrc.ac.uk/Ourresearch/Ethicsresearchguidance/Useofanimals/index.htm
http://ClinicalTrials.gov
http://www.consort-statement.org
http://www.consort-statement.org/checklists/view/32-consort/66-title
http://www.equator-network.org/reporting-guidelines/reporting-recommendations-for-tumo
http://datadryad.org
http://figshare.com
http://www.ncbi.nlm.nih.gov/gap
http://www.ebi.ac.uk/ega
http://biomodels.net/
http://biomodels.net/miriam/
http://jjj.biochem.sun.ac.za
https://osp.od.nih.gov/biosafety-biosecurity-and-emerging-biotechnology/
http://www.selectagents.gov/

Is the variance similar between the groups that are being statistically compared?	Yes

C- Reagents

6. To show that antibodies were profiled for use in the system under study (assay and species), provide a citation, catalog number and/or clone number, supplementary information or reference to an antibody validation profile. e.g., Antibodypedia (see link list at top right), 1DegreeBio (see link list at top right).	Table with antibody catalogue numbers are included
7. Identify the source of cell lines and report if they were recently authenticated (e.g., by STR profiling) and tested for mycoplasma contamination.	Cell lines were regularly tested for mycoplasma. Cell lines from prevously validated stocks were thawed every other month.

* for all hyperlinks, please see the table at the top right of the document

D- Animal Models

8. Report species, strain, gender, age of animals and genetic modification status where applicable. Please detail housing and husbandry conditions and the source of animals.	NA
9. For experiments involving live vertebrates, include a statement of compliance with ethical regulations and identify the committee(s) approving the experiments.	NA
10. We recommend consulting the ARRIVE guidelines (see link list at top right) (PLoS Biol. 8(6), e1000412, 2010) to ensure that other relevant aspects of animal studies are adequately reported. See author guidelines, under 'Reporting Guidelines'. See also: NIH (see link list at top right) and MRC (see link list at top right) recommendations. Please confirm compliance.	NA

E- Human Subjects

11. Identify the committee(s) approving the study protocol.	NA
12. Include a statement confirming that informed consent was obtained from all subjects and that the experiments conformed to the principles set out in the WMA Declaration of Helsinki and the Department of Health and Human Services Belmont Report.	NA
13. For publication of patient photos, include a statement confirming that consent to publish was obtained.	NA
14. Report any restrictions on the availability (and/or on the use) of human data or samples.	NA
15. Report the clinical trial registration number (at ClinicalTrials.gov or equivalent), where applicable.	NA
16. For phase II and III randomized controlled trials, please refer to the CONSORT flow diagram (see link list at top right) and submit the CONSORT checklist (see link list at top right) with your submission. See author guidelines, under 'Reporting Guidelines'. Please confirm you have submitted this list.	NA
17. For tumor marker prognostic studies, we recommend that you follow the REMARK reporting guidelines (see link list at top right). See author guidelines, under 'Reporting Guidelines'. Please confirm you have followed these guidelines.	NA

F- Data Accessibility

18: Provide a "Data Availability" section at the end of the Materials & Methods, listing the accession codes for data generated in this study and deposited in a public database (e.g. RNA-Seq data: Gene Expression Omnibus GSE39462, Proteomics data: PRIDE PXD000208 etc.) Please refer to our author guidelines for 'Data Deposition'.<br><br>Data deposition in a public repository is mandatory for:<br>a. Protein, DNA and RNA sequences<br>b. Macromolecular structures<br>c. Crystallographic data for small molecules<br>d. Functional genomics data<br>e. Proteomics and molecular interactions	Data avaliablity statement included in manuscript. No data needed to be deposited.
19. Deposition is strongly recommended for any datasets that are central and integral to the study; please consider the journal's data policy. If no structured public repository exists for a given data type, we encourage the provision of datasets in the manuscript as a Supplementary Document (see author guidelines under 'Expanded View' or in unstructured repositories such as Dryad (see link list at top right) or Figshare (see link list at top right).	NA
20. Access to human clinical and genomic datasets should be provided with as few restrictions as possible while respecting ethical obligations to the patients and relevant medical and legal issues. If practically possible and compatible with the individual consent agreement used in the study, such data should be deposited in one of the major public access-controlled repositories such as dbGAP (see link list at top right) or EGA (see link list at top right).	NA
21. Computational models that are central and integral to a study should be shared without restrictions and provided in a machine-readable form. The relevant accession numbers or links should be provided. When possible, standardized format (SBML, CellML) should be used instead of scripts (e.g. MATLAB). Authors are strongly encouraged to follow the MIRIAM guidelines (see link list at top right) and deposit their model in a public database such as Biomodels (see link list at top right) or JWS Online (see link list at top right). If computer source code is provided with the paper, it should be deposited in a public repository or included in supplementary information.	NA

G- Dual use research of concern

22. Could your study fall under dual use research restrictions? Please check biosecurity documents (see link list at top right) and list of select agents and toxins (APHIS/CDC) (see link list at top right). According to our biosecurity guidelines, provide a statement only if it could.	NO, no dual use research restrictions
