# Supplementary material for: The Hippo pathway drives the cellular response to hydrostatic pressure
Source: EMBO J. 2022 Jun 15;41(13):e108719. doi: 10.15252/embj.2021108719 (PMC9251841; doi:10.15252/embj.2021108719)

EV1A

293A **PhosTag (YAP)**

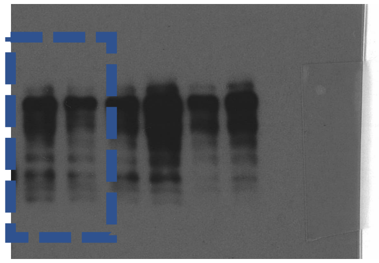

143B **PhosTag (YAP)**

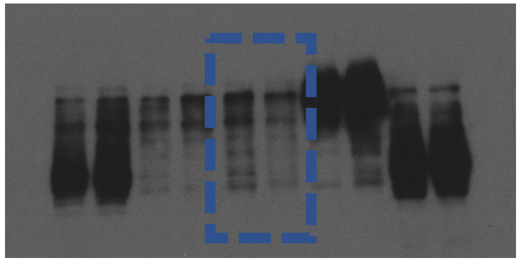

EV1B

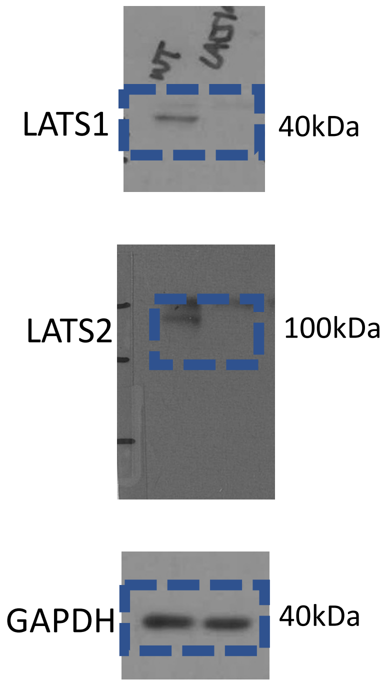

EV1C

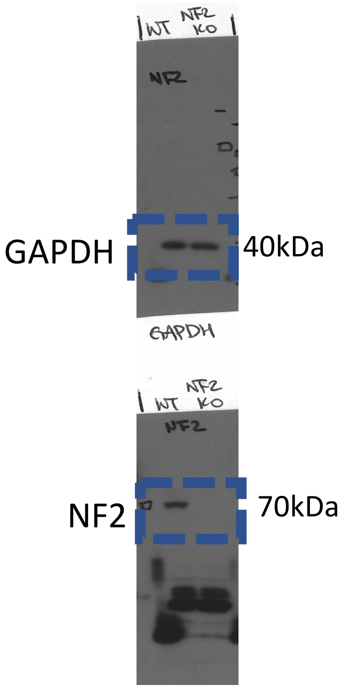

EV1D

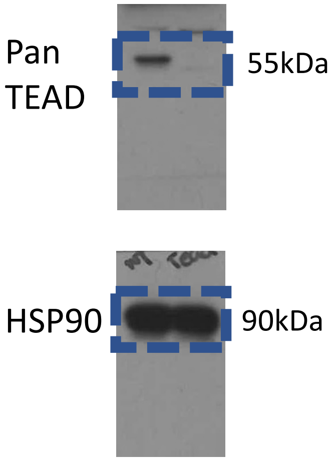

Supplement: Supplementary file 2 — Source Data for Expanded View [file EMBJ-41-e108719-s005.zip › Western_Blots_for_Park_et_al_EV1.zip!/Western_Blots_for_Park_et_al_EV1_part1.pdf]
