# Supplementary material for: The Hippo pathway drives the cellular response to hydrostatic pressure
Source: EMBO J. 2022 Jun 15;41(13):e108719. doi: 10.15252/embj.2021108719 (PMC9251841; doi:10.15252/embj.2021108719)
Supplement: Supplementary file 2 — Source Data for Expanded View [file EMBJ-41-e108719-s005.zip › Western_Blots_for_Park_et_al_EV1.zip!/Western_Blots_for_Park_et_al_EV1_part2.pdf]

EV1E

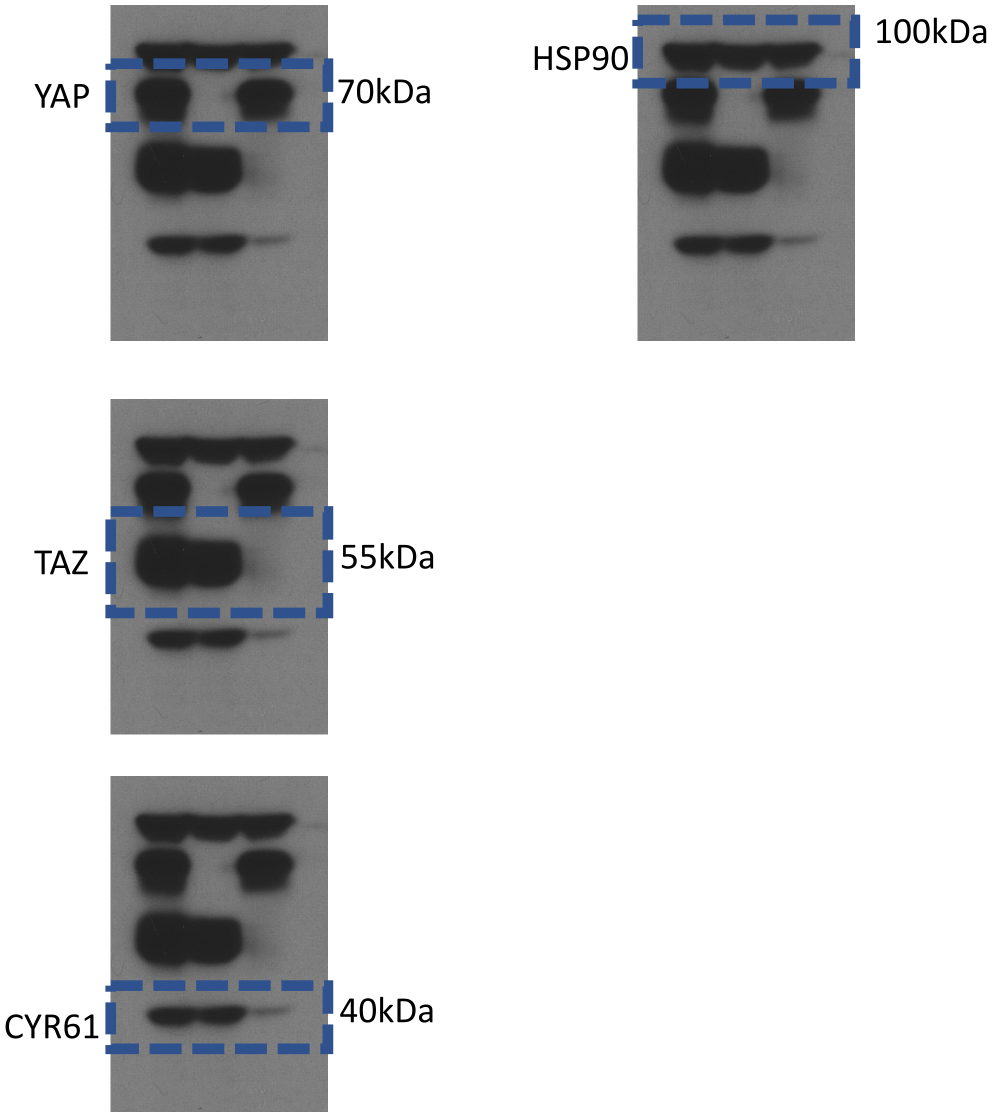
YAP
70kDa
HSP90
100kDa
TAZ
55kDa
CYR61
40kDa

EV1H

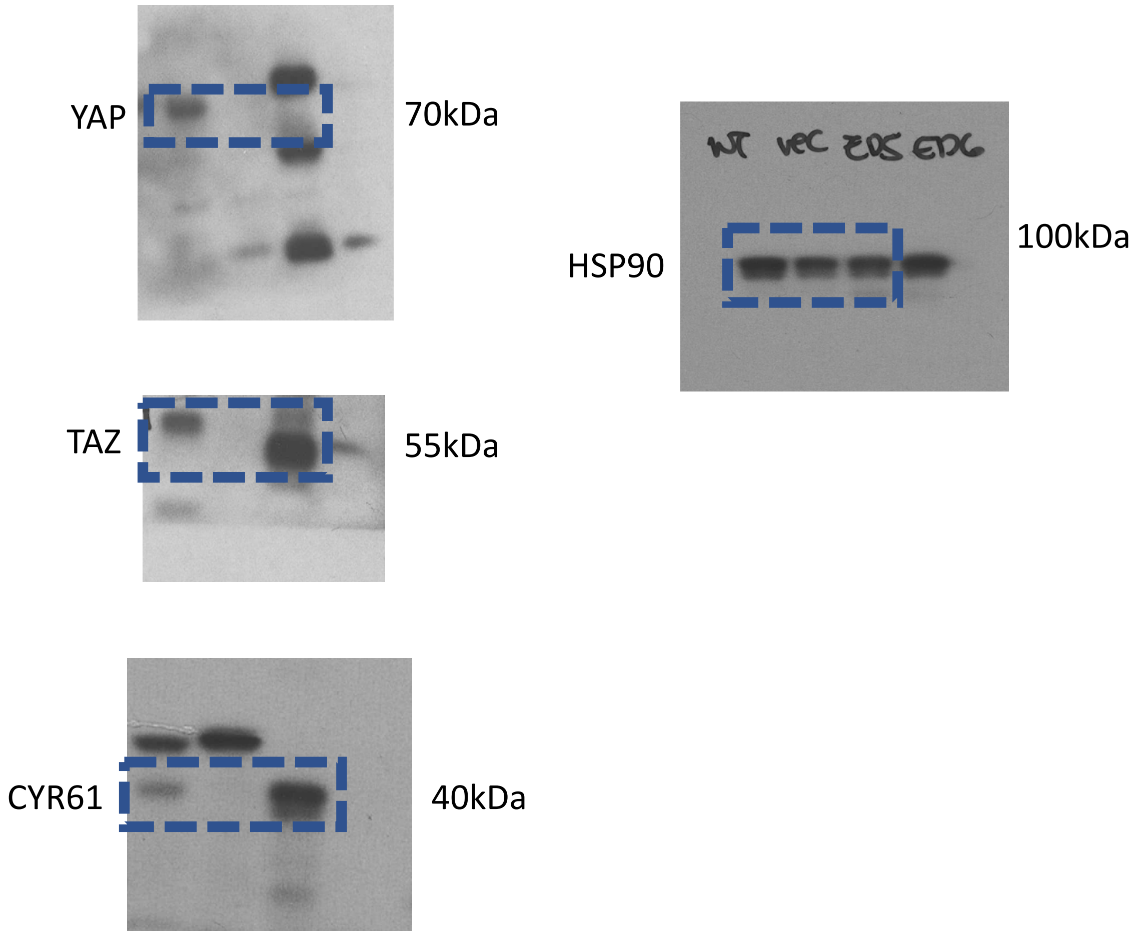
YAP
70kDa
TAZ
55kDa
CYR61
40kDa
HSP90
100kDa
